# Pulmonary Delivery of Aerosolized Chloroquine and Hydroxychloroquine to Treat COVID-19: *In Vitro* Experimentation to Human Dosing Predictions

**DOI:** 10.1208/s12248-021-00666-x

**Published:** 2022-02-07

**Authors:** Aditya R. Kolli, Tanja Zivkovic Semren, David Bovard, Shoaib Majeed, Marco van der Toorn, Sophie Scheuner, Philippe A. Guy, Arkadiusz Kuczaj, Anatoly Mazurov, Stefan Frentzel, Florian Calvino-Martin, Nikolai V. Ivanov, John O’Mullane, Manuel C. Peitsch, Julia Hoeng

**Affiliations:** PMI R&D, Philip Morris Products S.A., Quai Jeanrenaud 5, CH-2000 Neuchâtel, Switzerland

**Keywords:** Inhalation, Chloroquine, Hydroxychloroquine, Aerosol, PBPK, SARS-CoV-2

## Abstract

*In vitro* screening for pharmacological activity of existing drugs showed chloroquine and hydroxychloroquine to be effective against severe acute respiratory syndrome coronavirus 2. Oral administration of these compounds to obtain desired pulmonary exposures resulted in dose-limiting systemic toxicity in humans. However, pulmonary drug delivery enables direct and rapid administration to obtain higher local tissue concentrations in target tissue. In this work, inhalable formulations for thermal aerosolization of chloroquine and hydroxychloroquine were developed, and their physicochemical properties were characterized. Thermal aerosolization of 40 mg/mL chloroquine and 100 mg/mL hydroxychloroquine formulations delivered respirable aerosol particle sizes with 0.15 and 0.33 mg per 55 mL puff, respectively. *In vitro* toxicity was evaluated by exposing primary human bronchial epithelial cells to aerosol generated from Vitrocell. An *in vitro* exposure to 7.24 μg of chloroquine or 7.99 μg hydroxychloroquine showed no significant changes in cilia beating, transepithelial electrical resistance, and cell viability. The pharmacokinetics of inhaled aerosols was predicted by developing a physiologically based pharmacokinetic model that included a detailed species-specific respiratory tract physiology and lysosomal trapping. Based on the model predictions, inhaling emitted doses comprising 1.5 mg of chloroquine or 3.3 mg hydroxychloroquine three times a day may yield therapeutically effective concentrations in the lung. Inhalation of higher doses further increased effective concentrations in the lung while maintaining lower systemic concentrations. Given the theoretically favorable risk/benefit ratio, the clinical significance for pulmonary delivery of aerosolized chloroquine and hydroxychloroquine to treat COVID-19 needs to be established in rigorous safety and efficacy studies.

**Supplementary Information:**

The online version contains supplementary material available at 10.1208/s12248-021-00666-x.

**Figure:**
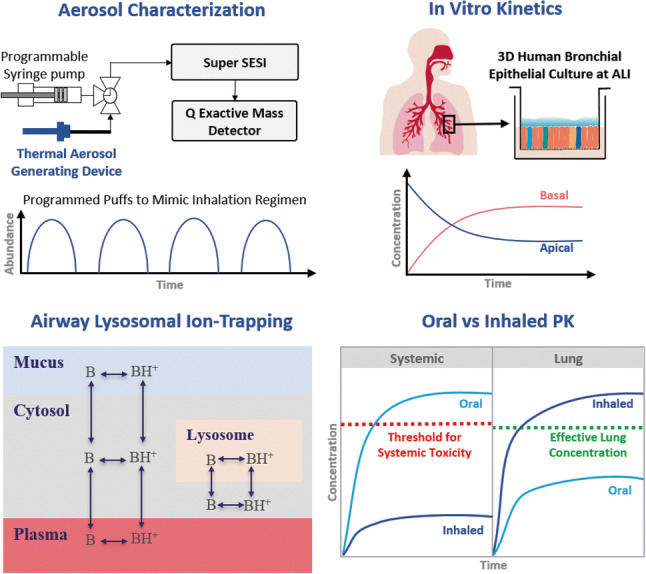
Graphical abstract

## Introduction

Severe acute respiratory syndrome coronavirus 2 (SARS-CoV-2) is a highly virulent strain of human coronavirus causing widespread acute respiratory disease. On 11 March 2020, the World Health Organization declared coronavirus disease of 2019 (COVID-19) a global pandemic. While three vaccines have been developed and received emergency use authorizations in several countries, the repurposing of existing drugs for short-term prophylaxis is potentially an immediate option [1]. An *in vitro* screening of existing drugs on Vero cells infected with SARS-CoV-2 showed chloroquine (CQ) and its analog hydroxychloroquine (HCQ) to be effective at both entry and post-entry stages of SARS-CoV-2 infection [1–3]. CQ and HCQ exhibit a wide spectrum of biological activity and are used in treating malaria, rheumatoid arthritis, and systemic lupus erythematosus [2]. The postulated mechanism of action of these compounds against COVID-19 is through increasing the pH of endosomes, lysosomes, and the cell membrane surface, thereby preventing the fusion of the virus with host cells and subsequent replication [1, 2] or by interfering with the glycosylation of angiotensin-converting enzyme 2 (ACE2) to reduce the binding efficiency between host cells and the spike protein on the surface of the coronavirus [4]. Accumulation of CQ or HCQ in lysosomes could also result in dysfunction of enzymes that enable proteolytic processing and post-translational modification of viral proteins [1, 2].

CQ and HCQ have three basic functional groups with pK_a_ values of 4.0, 8.4, and 10.2 and <4.0, 8.3, and 9.7, two of which are protonated at physiological pH. The unprotonated forms of CQ and HCQ diffuse spontaneously and rapidly across cell membranes and organelles to acidic cytoplasmic vesicles such as endosomes or lysosomes or Golgi vesicles. As the unprotonated forms get protonated and trapped in the acidic compartments, the concentrations of acidic compartments rise significantly [5, 6]. For example, the concentration of CQ in lysosomes is predicted to be approximately 1000-fold higher than in the cytosol [5]. On oral administration, lysosome-rich tissues such as lungs, liver, kidney, and heart accumulate significantly higher concentrations, leading to dose-limiting toxicity [7]. The concentration of a drug in human tissues and cellular lysosomes can be simulated using physiologically based pharmacokinetic (PBPK) models, a technique that integrates physicochemical properties and physiological human parameters to predict absorption, distribution, metabolism, and elimination of compounds. A validated mechanistic PBPK model could serve as a valuable tool for identifying dosing regimens that are safe and effective for the treatment of COVID-19.

Clinical trials involving oral dosing of CQ and HCQ were widely undertaken in various countries, and several organizations have approved their use on a compassionate basis to treat patients [8–11]. Most treatment schedules included a high loading dose and a maintenance dose to obtain efficacious concentrations in the lung. A lower dose of CQ (450 mg b.i.d., for 1 day and 450 mg q.d. for 4 days) resulted in adverse events related to cardiac QT interval prolongation, which only increased in higher dose groups [12]. Similarly, oral dosing of HCQ has been reported with instances of renal, retinal, and cardiotoxicity. Most clinical trials early on during the pandemic included terminally ill patients [13] and were more likely to have patients with preexisting conditions, such as coronary artery disease, congestive heart failure, and a history of arrhythmias. Whether patients with existing cardiovascular disease or cardiovascular injury are more prone to ventricular arrhythmias following CQ treatment is unknown. It has also been reported that high oral doses of CQ (600 mg twice daily for 10 days or a total dose of 12 g) may be associated with significant cardiac risks [12]. Although the outcomes of several such trials for CQ and HCQ have been inconclusive, the numerous reports of adverse events led to withdrawal of oral administration for COVID-19 [8]. A recent multi-center retrospective observational study in the USA indicated improved survival among patients who received HCQ (66% reduction in the hazard ratio) and patients who received HCQ combined with azithromycin than among those who did not receive HCQ and those who received azithromycin alone. Moreover, enhanced survival among patients who received HCQ persisted for 4 weeks from admission [14, 15]. As of 18 January 2021, 220 clinical trials were either recruiting, active, active not recruiting, or enrolled by invitation ongoing worldwide to treat or prevent COVID-19 by CQ or HCQ [www.clinicaltrials.gov]. Workers in healthcare settings such as hospitals, clinics, and long-term care facilities are at a higher risk of exposure to the SARS-CoV-2 virus than the general population. An ongoing randomized clinical trial (ClinicalTrials.gov identifier: NCT04334148) of more than 15,000 healthcare workers in the USA evaluates whether HCQ can prevent COVID-19 infection in healthcare workers [16].

Pulmonary drug delivery enables the direct delivery of compounds to the respiratory tract and could yield high local tissue concentrations rapidly while minimizing systemic exposure [17]. Increased local concentrations could improve the therapeutic index at the target site. Conversely, depending on the compound and the formulation’s physiochemical properties, inhalation also enables rapid systemic delivery of compounds. Hence, it is critical to evaluate aerosol characteristics and optimize inhalation dosing regimens. In this study, we formulated and characterized CQ and HCQ for delivery as potentially therapeutic inhalable aerosols, evaluated the *in vitro* effects of the aerosols on three-dimensional(3D) organotypic human bronchial epithelial cultures (HBEC), simulated kinetics across isolated perfused mice lung (IPML), and developed a translational mechanistic inhalation PBPK model to predict pulmonary and systemic exposures following various inhalation dosing regimens.

## Methods

### Compound Synthesis and Aerosol Formulation

CQ [18] and HCQ [19] were synthesized according to published procedures at WuXi AppTec (Wuhan, China). The synthesized CQ and HCQ had a purity of 98.3% and 99.7%, respectively. Multiple liquid formulations at different concentrations were prepared by dissolving CQ or HCQ in propylene glycol (PG). The solubility of CQ and HCQ in PG was assessed by liquid chromatography high-resolution mass spectrometry (LC-HR-MS).

### Aerosol Generation and Characterization

Aerosol from the liquid formulation was generated by thermal aerosolization [20]. The temperature of the heater was maintained at 200–220°C. The thermal aerosol-generating device caps were filled with either CQ or HCQ liquid formulation. The particle size distribution of the aerosols was measured by connecting the thermal aerosol-generating device to a programmable syringe pump and aerodynamic particle sizer (model 3321, TSI Incorporated, Shoreview, MN, USA) as shown in Figure S1. To reach an operational flowrate of 5 L/min and stay within the limits of detection of the large particle number densities obtained in the experiment, the single programmable syringe pump was connected with a 3302A aerosol diluter (TSI Incorporated, Shoreview, MN, USA) upstream of the aerosol particle sizer by using a 30-cm conductive tube with a 1-cm inner diameter. To avoid the build-up of negative pressure in the connection, a Y-piece open to the surroundings was installed between the syringe pump and aerosol particle sizer. In this configuration, the difference between the volume flow supplied by the syringe pump and the volume flow required by the aerosol particle sizer is compensated by the influx of surrounding air into the system. The samples were diluted 100-fold using the aerosol diluter upstream of the aerosol particle sizer to maintain appropriate flows for the particle size measurements and chemical characterization. The discharging periods from the syringe pump varied between 3 s (average, 1.1 L/min) for the aerosol particle sizer and 8 s (average, 0.41 L/min) for *in vitro* aerosol delivery.

### Analytical Measurements

Thermal aerosol-generating device connected to programmable dual syringe (PDS) pump was attached to a SUPER SESI (Fossil Ion Technology, Malaga, Spain) interfaced with a Q Exactive HF system (Thermo Fisher Scientific, Waltham, MA, USA). The generated aerosol was pushed through a Cambridge filter pad connected to an impinger filled with 5 mL of ethanol to assess the amount of CQ and HCQ transferred from the liquid to the aerosol using LC-HR-MS(Figure S2). Compound extraction from Cambridge filter pads was performed by adding 5 mL of ethanol from the impinger and another 5 mL of fresh ethanol to the filter pad. The two fractions were combined (total volume, 10 mL) for quantification. Chemical analyses for drug solubility and transfer rate assessment were performed by liquid chromatographer equipped with a HILIC BEH amide column (50 × 3 mm; 1.7 μm, Waters, Milford, MA, USA) coupled to a high-resolution accurate mass spectrometer (Vanquish Duo – Q Exactive HF system, LC-HR-MS, Thermo Fisher Scientific, Waltham, MA, USA). The mobile phases were composed of acetonitrile containing 0.1% formic acid and 10 mM ammonium formate. The samples were diluted to fit the calibration curve built from nine calibrant levels (5–100 ng/mL). A volume of 5 μL diluted solution was injected. Mass spectrometry detection was performed using the positive electrospray ionization mode with a mass resolution of 60,000 by scanning full-scan mass at *m/z* 50–350.

### Cell Culture

3D organotypic HBEC grown at air-liquid interface (ALI) were prepared from primary human bronchial epithelial cells (Lonza, Basel, Switzerland) as previously described by Bovard et al. [21]. Briefly, primary normal human bronchial epithelial (NHBE; donor characteristics: 60-year-old, Black male) cells (Lonza, Basel, Switzerland) were first cultured in PneumaCult-Ex Plus™ medium (STEMCELL Technologies, Vancouver, Canada) at 37°C under 5% CO_2_ and 90% relative humidity. Once the cells were approximately 80% confluent, they were detached from the flask by using trypsin–EDTA (ethylenediaminetetraacetic acid; Lonza), and 50,000 cells were seeded on a 6.5-mm diameter Transwell® insert with a 0.4-μm pore size (Corning®, Corning, NY, USA). Both the apical and basal sides of the inserts were filled with PneumaCult-Ex Plus™ medium, and the cells were incubated for 3 days. Subsequently, the cells were air-lifted by removing the apical medium; the basal medium was replaced with PneumaCult™-ALI medium (STEMCELL Technologies), which was renewed every 2 or 3 days. The cultures were considered mature after 4 weeks at the air-liquid interface (ALI) and used for experiments between week 15 and 20.

### Vitrocell Aerosol Exposure

**Figure 1:**
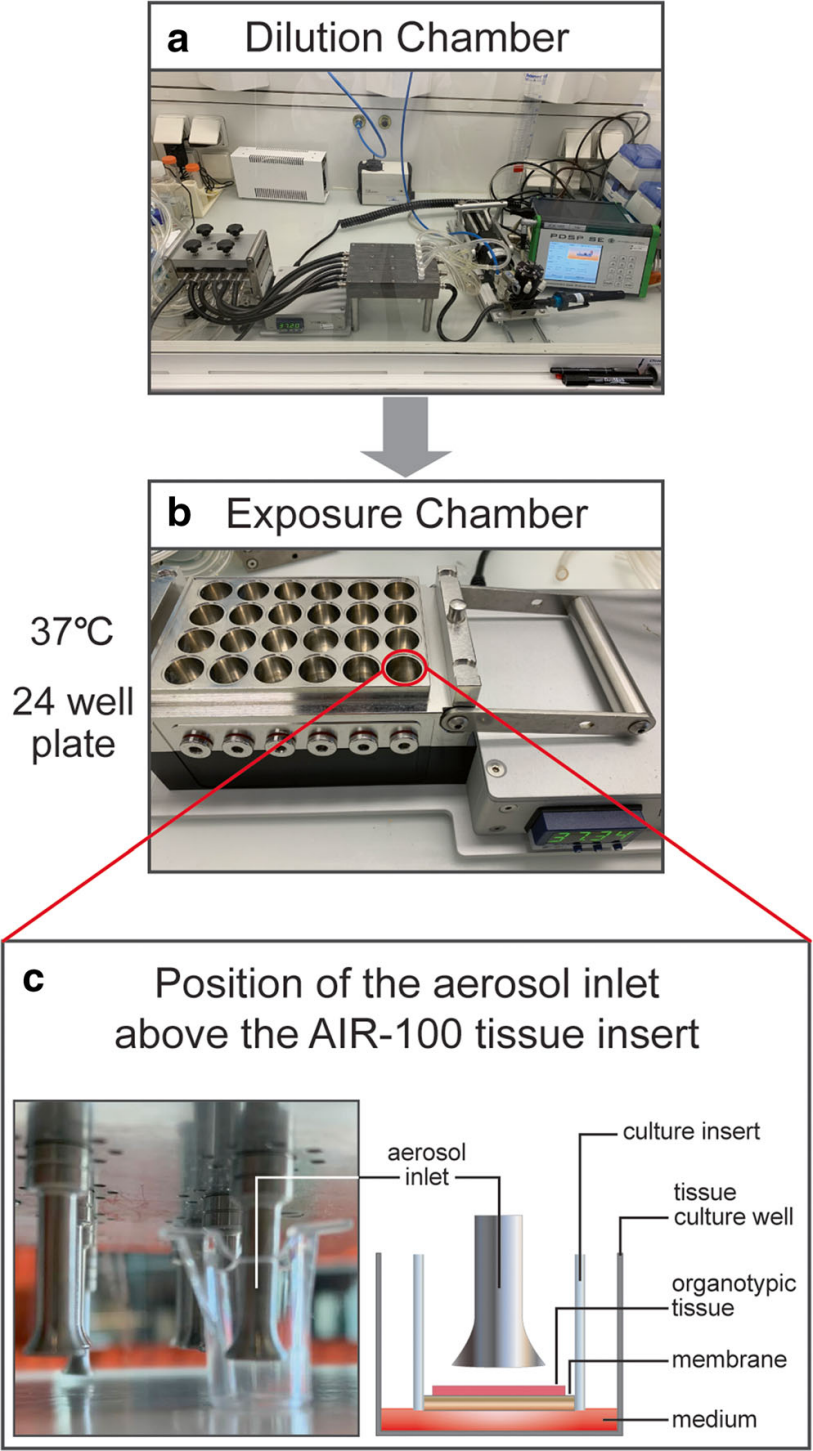
Schematic representation of the *in vitro* aerosol generation and exposure system. The aerosol generated passes through **a** the dilution chamber without any dilution into **b** the exposure chamber with **c**trumpet-like outlets to the cell culture inserts. Each cell culture insert contains three-dimensional organotypic human bronchial airway cultures at the air-liquid interface on a porous membrane and culture medium at the bottom

The Vitrocell 24 exposure system (Vitrocell Systems GmbH, Waldkirch, Germany) and the PDSP were installed inside a biosafety cabinet. The generated aerosol (with a 55 mL puff volume, 3-s puff duration, and 30-s puff interval) from a 25 mg/mL CQ and HCQ liquid formulation was transferred via PDS pump to the exposure top and distributed into the cultivation base module via port ejectors (trumpets) under negative pressure. Organotypic human bronchial epithelial cultures grown at the ALI were placed in the cultivation base module, maintained at 37°C, and exposed to aerosol concentrations on their apical side (Figure 1). The cell cultures were exposed to 25, 50, and 100 puffs of CQ or HCQ aerosol, 100 puffs of synthetic air (85% nitrogen and 15% oxygen; Praxair, Düsseldorf, Germany), and 100 puffs of propylene glycol as a control.

CQ and HCQ deposited in the exposure chamber were trapped using Transwell inserts (Cat. No. 3470, Corning, New York, USA) containing ultra-pure H_2_O. The inserts with 110 μL of ultra-pure H_2_O were located in the base module of the Vitrocell 24 exposure system and exposed together with the 3D organotypic cell cultures in each exposure experiment. Apical and basal compartmental kinetics for HCQ were measured as described in supplementary methods.

### Measurement of Ciliary Beating Frequency

We measured ciliary beating frequency and ciliary beating active area in the cultures using an inverted microscope (Zeiss, Oberkochen, Germany) equipped with a 4× objective and a 37°C chamber and connected to a high-speed camera (Basler AG, Ahrensburg, Germany). Short movies composed of 512 frames recorded at 120 images per second were analyzed using the SAVA software (Ammons Engineering, Clio, MI, USA). Measurements were made pre- and 24-h post-exposure to air, vehicle, or drug-containing aerosol.

### Measurement of Transepithelial Electrical Resistance (TEER)

TEER was measured in the cultures before the exposure and 24-h post-exposure using an EndOhm 6 chamber (WPI, Sarasota, FL, USA) connected to an EVOM epithelial voltohmmeter (WPI) according to the manufacturer’s instructions. The value displayed by the voltohmmeter was multiplied by the surface of the inserts (0.33 cm^2^) to obtain the resistance value in the total area (Ω × cm^2^).

### Cell Viability

We evaluated the viability of the 3D organotypic cultures 24-h post-exposure by measuring adenosine triphosphate (ATP) content using a CellTiter-Glo 3D cell viability kit (Promega, Madison, WI, USA). CellTiter-Glo reagent (150 μL) was added to the apical surface; after 30 min, 50 μL of CellTiter-Glo reagent was transferred from the apical surface of the tissues into an opaque-walled 96-well plate, and luminescence in relative light units was measured using a FLUOstar Omega microplate reader (BMG Labtech, Ortenberg, Germany).

### Measurement of *In Vitro* Transport Kinetics

After exposure to 25, 50, and 100 puffs of Vitrocell-generated CQ and HCQ aerosol, cell culture inserts with 3D organotypic HBEC at the ALI were transferred to a 24 well plate with 750 μL of fresh PneumaCult-ALI medium. From the basolateral compartment, 250 μL of the medium was collected after 1-, 2-, and 24-h post-exposure. At 24-h post-exposure, 200 μL of PneumaCult-ALI medium was added to the apical surface fluid, and the apical volume was collected after 5 min. Aliquots of prepared initial formulation and test samples were stored at −80°C after collection for analysis.

### Modeling Ion-Trapping Kinetics

The diffusive flux of diprotic bases between compartments was calculated based on the model developed by Trapp et al. [5]. Briefly, the drug transport across the compartment was calculated as the sum of the diffusive flux of neutral species calculated by Fick’s first law and ionic species by the Nernst–Planck equation (Eq. 1).
1where *J*_*net*_ is the total net diffusion flux, *P* is the permeability, *C* is concentration (or activity of the compound), and is the electric charge (0 for neutral; +1 and +2 for ionic species), *F* is the Faraday constant, *E* is the membrane potential, *R* is the real gas constant, and *T* is the temperature. The subscripts represent the fractions of neutral (*n*) and ionic (*d*), species present inside (*i*) and outside (*o*) the compartment. The neutral fraction of the drug (*f*_*n*_) of the drug available for diffusion was calculated using Eq. 2.
2

which accounts for the water fraction (*W*), lipid binding (*L*), sorption coefficients (*K*), and the ionic activity coefficients (*γ*) for the compartment [5]. The ionic fraction (*f*_*dz﻿*_) of the drug was calculated using Eq﻿.3.
3

The ratio of ionic to neutral fractions  of the drug in the given charged state was calculated using the Henderson–Hasselbalch equation by accounting for the activity of dissolved molecules (Eqs. 4 and 5):

4 $$ {D}_{d1}=\frac{10^{\left(p{K}_{a1}- pH\right)}}{1+{10}^{\left(p{K}_{a1}- pH\right)}+{10}^{\left(p{K}_{a1}- pH\right)}+{10}^{\left(p{K}_{a2}- pH\right)}} $$

5 $$ {D}_{d2}=\frac{10^{\left(p{K}_{a1}- pH\right)}+{10}^{\left(p{K}_{a2}- pH\right)}}{1+{10}^{\left(p{K}_{a1}- pH\right)}+{10}^{\left(p{K}_{a1}- pH\right)}+{10}^{\left(p{K}_{a2}- pH\right)}} $$

Additionally, the ionic activity coefficient (*γ*) and the sorption coefficients  for neutral and ionic species were determined by the octanol-water partition coefficient  and cytosolic ionic strength (*I*_*o*_) using Eqs. 6–9. The lipophilicity of ionic species was set 6.5 log-units lower per charge to neutral species [5]. The permeability  of a given species is calculated using logarithmic octanol-water partition coefficient (*logKow*) and relative diffusivity factor (*Δs*) capturing the organic drug-specific diffusion coefficient [22] as in Eq. 10.

6 $$ {\gamma}_n={10}^{0.3\ast {I}_o} $$

78910

Earlier studies have indicated that accumulation of diprotic weak bases affects lysosomal *pH* even with lysosomal buffering [23, 24]. The changes in lysosomal *pH* were described using Eq. 11.

11 $$ p{H}_{lys}=p{H}_{lys,t=0}-\frac{C_{lys}}{\beta } $$

where *pH*_*lys*, *t* = 0_ is the initial *pH* of the lysosome, *C*_*lys*_ is the concentration of drug in the lysosome, and *β* is the lysosomal buffering capacity [7, 25]. The model parameters are listed in Table I.

**Table I:** Physicochemical Properties of Compounds and Ion-Trapping Model Parameters

*Physicochemical properties of chloroquine (CQ) and hydroxychloroquine (HCQ)*							
**Compound**	*pK*_*a*1_	*pK*_*a*2_	*logKow*	*f*_*u*_	*BP*	Diffusivity factor, *Δ*s	*DC* (cm^2^/sec) ^a^
CQ	9.4 [59]	8.2 [59]	4.89 [59]	0.6 [59]	8.0 [78]	7.4 ^b^	6.49E-11
HCQ	9.67 [7]	8.27 [7]	3.84 [7]	0.45 [7]	7.2 [7]	6.8 ^b^	6.42E-11
*Ion-trapping model parameters*							
**Parameter**	Diameter		Water	Lipid	Ion strength	Membrane potential	Lysosomal buffering
**Symbol (units)**	*d* (m)		*W* (g/g)	*L* (g/g)	*I*_*o*_ (mol)	*E* (mV)	*β* (mM)
Cell [5]	1E-5		0.95	0.05	0.3	-70	
Lysosome [5]	0.5E-6 [79]		0.95	0.05	0.3	100	46 [25]

^*a*^*Calculated;*^*b*^*optimized to MacIntyre et al.* [79]

### Modeling *In Vitro* HBEC and *Ex Vivo* IPML Kinetics

The *in vitro* HBEC model consisted of apical mucus (*muc*), periciliary layer (*pcl*), cytosol (*cyt*), lysosomal (*lys*), and basal (*bas*) compartments. The lysosomal compartment was nested within the cytosol compartment. The diffusive flux of diprotic bases between the periciliary layer and cytosol, cytosol and lysosome, and cytosol and basal compartments was calculated using Eqs. 1–10 [5]. The model also incorporated active transport of drugs from the cytosol to the periciliary layer via the P-gp efflux transporter and was modeled using the parameters obtained from Price et al. [26]. The differential equations describing the changes in concentrations in compartments representing the human bronchial epithelium at the ALI are mentioned in Eqs. 12–16.

12 $$ \frac{\mathrm{d}}{\mathrm{d}\mathrm{t}}{\mathrm{C}}_{\mathrm{muc}}=\frac{1}{{\mathrm{V}}_{\mathrm{muc}}}\ \left(-\frac{DC\ast {\mathrm{SA}}_{\mathrm{insert}}}{{\mathrm{T}}_{\mathrm{muc}}}\ast \left({\mathrm{C}}_{\mathrm{muc}}-{\mathrm{C}}_{\mathrm{pcl}}\right)\right) $$

13 $$ \frac{\mathrm{d}}{\mathrm{d}\mathrm{t}}{\mathrm{C}}_{\mathrm{pcl}}=\frac{1}{{\mathrm{V}}_{\mathrm{pcl}}}\ \left(\frac{DC\ast S{A}_{insert}}{{\mathrm{T}}_{\mathrm{pcl}}}\left({\mathrm{C}}_{\mathrm{muc}}-{\mathrm{C}}_{\mathrm{pcl}}\right)-S{A}_{insert}\ast \left({\mathrm{J}}_{\mathrm{pcl}-\mathrm{cyt}}\ast {\mathrm{C}}_{\mathrm{pcl}}-{\mathrm{J}}_{\mathrm{cyt}-\mathrm{pcl}}\ast {C}_{\mathrm{cyt}}\right)+\frac{Vma{x}_{pgp}\ast S{A}_{insert}\ast {f}_n\ast {\mathrm{C}}_{\mathrm{cyt}}}{{\mathrm{Km}}_{\mathrm{pgp}}+{\mathrm{f}}_{\mathrm{n}}\ast {\mathrm{C}}_{\mathrm{cyt}}}\ \right) $$

14 $$ \frac{\mathrm{d}}{\mathrm{d}\mathrm{t}}{\mathrm{C}}_{\mathrm{cyt}}=\frac{1}{{\mathrm{V}}_{\mathrm{cyt}}-{V}_{lys}}\left(S{A}_{insert}\ast \left({\mathrm{J}}_{\mathrm{pcl}-\mathrm{cyt}}\ast {\mathrm{C}}_{\mathrm{pcl}}-{\mathrm{J}}_{\mathrm{cyt}-\mathrm{pcl}}\ast {C}_{\mathrm{cyt}}\right)+{\mathrm{SA}}_{\mathrm{insert}}\ast \left({\mathrm{J}}_{\mathrm{bas}-\mathrm{cyt}}\ast {\mathrm{C}}_{\mathrm{bas}}-{\mathrm{J}}_{\mathrm{cyt}-\mathrm{bas}}\ast {\mathrm{C}}_{\mathrm{cyt}}\right)-{\mathrm{SA}}_{\mathrm{lys}}\ast \left({\mathrm{J}}_{\mathrm{cyt}-\mathrm{lys}}\ast {C}_{\mathrm{cyt}}-{\mathrm{J}}_{\mathrm{lys}-\mathrm{cyt}}\ast {C}_{\mathrm{lys}}\right)-\kern0.5em \frac{Vma{x}_{pgp}\ast S{A}_{insert}\ast {\mathrm{f}}_{\mathrm{n}}\ast {\mathrm{C}}_{\mathrm{cyt}}}{{\mathrm{Km}}_{\mathrm{pgp}}+{\mathrm{f}}_{\mathrm{n}}\ast {\mathrm{C}}_{\mathrm{cyt}}}\ \right) $$

15 $$ \frac{\mathrm{d}}{\mathrm{d}\mathrm{t}}{\mathrm{C}}_{\mathrm{lys}}=\frac{1}{{\mathrm{V}}_{\mathrm{lys}}}\ \left(\ {\mathrm{SA}}_{\mathrm{lys}}\ast \left({\mathrm{J}}_{\mathrm{cyt}-\mathrm{lys}}\ast {\mathrm{C}}_{\mathrm{cyt}}-{\mathrm{J}}_{\mathrm{lys}-\mathrm{cyt}}\ast {\mathrm{C}}_{\mathrm{lys}}\right)\ \right) $$

16 $$ \frac{\mathrm{d}}{\mathrm{d}\mathrm{t}}{\mathrm{C}}_{\mathrm{bas}}=\frac{1}{{\mathrm{V}}_{\mathrm{bas}}}\ \left(-{\mathrm{SA}}_{\mathrm{insert}}\ast \left({\mathrm{J}}_{\mathrm{bas}-\mathrm{cyt}}\ast {\mathrm{C}}_{\mathrm{bas}}-{\mathrm{J}}_{\mathrm{cyt}-\mathrm{bas}}\ast {\mathrm{C}}_{\mathrm{cyt}}\right)\right) $$

where *C* is the concentration, *J* is the total permeability of neutral and ionic fractions, SA_insert_ is the surface area of the insert, *T* is thickness, and *V* is the volume of the compartment. The total surface area of lysosomes (*SA*_*lys*_) was derived by calculating the surface area of a single lysosome (assuming it to be spherical with a diameter, *d*) and scaled based on total lysosomal volume. For the P-gp transport kinetics, only the unbound neutral fraction is assumed to be transported and implemented using a in Michaelis–Menten equation. The diffusion coefficient (DC) of the deposited drug across the mucus was calculated using the Hayduk–Laudie equation [27] by incorporating the viscosity of airway mucus and is shown in Eq.17.

17 $$ \mathrm{DC}=\frac{1.36\times {10}^{-4}}{\mu_2^{1.14}\ {V}_b^{0.59}} $$

where *μ*_2_, viscosity of mucus, was 15 × 10^3^ centipoise [28], and the calculated *V*_*b*_, LeBas molar volume, for CQ was 427 cm^3^/mol and HCQ was 434 cm^3^/mol.

A six-compartment*in silico* model representing the pulmonary alveolar region of an *ex vivo* IPML was developed. The transport kinetics had a similar formalism as the *in vitro* model and described the concentration changes in the surfactant (*muc*), cytosol, lysosome, interstitial (*inter*), vascular (*vas*), and reservoir (*res*) compartments. During the flow of perfusate, an instantaneous equilibrium was assumed between the vascular and interstitial compartments for unbound drug concentrations. The equations describing the concentrations changes in compartments representing the *ex vivo* IPML are given in Eqs. 18–23.

18 $$ \frac{\mathrm{d}}{\mathrm{d}\mathrm{t}}{\mathrm{C}}_{\mathrm{muc}}=\frac{1}{{\mathrm{V}}_{\mathrm{muc}}}\ \left({\mathrm{SA}}_{\mathrm{PA}}\ast \left({\mathrm{J}}_{\mathrm{cyt}-\mathrm{muc}}\ast {\mathrm{C}}_{cyt}-\kern0.5em {\mathrm{J}}_{\mathrm{muc}-\mathrm{cyt}}\ast {\mathrm{C}}_{\mathrm{muc}}\right)+\frac{{\mathrm{V}\mathrm{max}}_{\mathrm{pgp}1}\ast {\mathrm{SA}}_{\mathrm{PA}}\ast {\mathrm{f}}_{\mathrm{n}}\ast {\mathrm{C}}_{cyt}}{{\mathrm{Km}}_{\mathrm{pgp}1}+{\mathrm{f}}_{\mathrm{n}}\ast {\mathrm{C}}_{cyt}}+\frac{{\mathrm{V}\mathrm{max}}_{\mathrm{pgp}2}\ast {\mathrm{SA}}_{\mathrm{PA}}\ast {\mathrm{f}}_{\mathrm{n}}\ast {\mathrm{C}}_{cyt}}{{\mathrm{Km}}_{\mathrm{pgp}2}+{\mathrm{f}}_{\mathrm{n}}\ast {\mathrm{C}}_{cyt}}\right) $$

19 $$ \frac{d}{dt}{C}_{cyt}=\frac{1}{V_{cyt}-{V}_{lys}}\left(-\frac{\  Vma{x}_{pgp1}\ast {\mathrm{SA}}_{\mathrm{PA}}\ast {f}_n\ast {C}_{cyt}}{K{m}_{pgp1}+{f}_n\ast {C}_{cyt}}-\frac{Vma{x}_{pgp2}\ast {\mathrm{SA}}_{\mathrm{PA}}\ast {f}_n\ast {C}_{cyt}}{K{m}_{pgp2}+{f}_n\ast {C}_{cyt}}-{\mathrm{SA}}_{\mathrm{PA}}\ast \left({J}_{cyt- muc}\ast {C}_{cyt}-{J}_{muc- cyt}\ast {C}_{muc}\right)-S{A}_{lys}\ast \left({J}_{cyt-l\mathrm{y}s}\ast {C}_{cyt}-{J}_{lys- cyt}\ast {C}_{lys}\right)+S{A}_{PA}\ast \left({J}_{out- cyt}\ast {C}_{inter}-{J}_{cyt- out}\ast {C}_{cyt}\right)\right) $$

20 $$ \frac{d}{dt}{C}_{lys}=\frac{1}{V_{lys}}\ \left(S{A}_{lys}\ast \left({J}_{cyt- lys}\ast {C}_{cyt}-{J}_{lys- cyt}\ast {C}_{lys}\right)\right) $$

21 $$ \frac{d}{dt}{C}_{inter}=\frac{1}{V_{inter}}\left({Q}_{PFR}\ast \left({C}_{vas}-{C}_{\mathrm{i} nter}\right)-S{A}_{PA}\ast \left({J}_{out- cyt}\ast {C}_{inter}-{J}_{cyt- out}\ast {C}_{cyt}\right)\right) $$

22 $$ \frac{d}{dt}{C}_{vas}=\frac{1}{V_{vas}}\ \left({Q}_{PFR}\ast \left({C}_{res}-{C}_{vas}\right)-{Q}_{PFR}\ast \left({C}_{vas}-{C}_{inter}\right)\right) $$

23 $$ \frac{d}{dt}{C}_{res}=\frac{1}{V_{res}}\ \left({Q}_{PFR}\ast \left({C}_{vas}-{C}_{res}\right)\right) $$

where *Q*_PFR_ is the perfusate flow rate, SA_PA_ is the surface area of pulmonary alveolar region in the lung, and Vmax_pgp_ and Km_pgp_ are Michaelis–Menten reaction rate parameters for P-gp transporter for variant-1 and variant-2. The volumes for cytosol (*V*_cyt_), interstitial (*V*_inter_), and vascular (*V*_vas_) compartments were fractionated from the tissue volumes calculated based on the surface area (SA_PA_) and tissue thickness (*T*_tissue_). The *in vitro* HBEC model and *ex vivo* IPML model parameters are listed in Table II.

**Table II:** Model Parameters for *In Vitro* 3D Human Bronchial Epithelial Culture Model and *Ex Vivo* Isolated Perfused Mice Lung Model

**Parameter**	**Symbol**	**Unit**	**Value**	**Parameter**	**Symbol**	**Value**
*In vitro* 3D human bronchial epithelial cell culture model						
Mucus thickness	*T*_*muc*_	cm	3E-4 ^a^	Mucus pH	*pH*_*muc*_	7.2 [37]
Periciliary layer thickness	*T*_*pcl*_	cm	4E-4 ^a^	Periciliary layer pH	*pH*_*pcl*_	7.2 [37]
Cytosol layer thickness	*T*_*cyt*_	cm	40E-4 ^a^	Cytosol pH	*pH*_*Tiss*_	7.2 ^d^
Basal volume	*V*_*bas*_	mL	0.25 ^a^	Basal pH	*pH*_*Basal*_	7.4 ^d^
Lysosomal volume	*V*_*lys*_	%	8 [39] ^b^	Lysosome pH	*pH*_*lys*_	4.7 [79]
Surface area of insert	*SA*_*insert*_	cm^2^	0.33 ^a^			
P-gp kinetics	*Vmax*_*pgp*_	ng/cm^2^/min	4.2E6 [26]			
P-gp kinetics	*Km*_*pgp*_	ng/mL	3.86E3 [26]			
Ex Vivo Isolated Perfused Mice Lung Model						
Surfactant thickness	*T*_*muc*_	cm	1E-5 [80]	Mucus pH	*pH*_*muc*_	7.0 [41]
Cellular layer thickness	*T*_*tissue*_	cm	4E-4 [29]	Cytosol pH	*pH*_*cyt*_	7.0 [42]
Reservoir volume	*V*_*res*_	mL	10 [26]	Lysosome pH	*pH*_*lys*_	5 [5]
Lysosome volume	*V*_*lys*_	%	0.1 [7] ^b^	Interstitial pH	*pH*_*inter*_	7.4 ^d^
Interstitial volume	*V*_*inter*_	%	33.6 [81] ^c^	Reservoir pH	*pH*_*res*_	7.4 ^d^
Vascular volume	*V*_*vas*_	%	18.5 [82] ^c^			
Perfusate flow rate	*Q*_*PFR*_	mL/min	1 [26]			
Surface area of PA lung	*SA*_*PA*_	cm^2^	500 [29]			
P-gp variant 1	*Vmax*_*pgp*1_	ng/cm^2^/min	4E6 [26]			
P-gp variant 1	*Km*_*pgp*1_	ng/mL	2.75E3 [26]			
P-gp variant 2	*Vmax*_*pgp*2_	ng/cm^2^/min	5.44E6 [26]			
P-gp variant 2	*Km*_*pgp*2_	ng/mL	3.38E3 [26]			

^*a*^*Measured;*^*b*^*% cellular volume;*^*c*^*% total tissue volume;*^*d*^*fixed*

### PBPK Model Development

A flow-limited PBPK model of CQ and HCQ, consisting of 16 tissue compartments including the regional respiratory tract, was developed. Each tissue nested a lysosomal compartment as described by Trapp et al. and Collins et al. [5, 7]. A general mass balance equation and the lysosomal kinetics for a single-tissue compartment are described in Eqs. 24 and 25.

24 $$ \frac{d}{dt}{C}_{tissue}=\frac{1}{\left({V}_{tissue}-{V}_{tissue lys}\right)}\left({Q}_{tissue}\ast \left({C}_{arterial}-\frac{C_{tissue}\ast BP}{f_u\ast {K}_{T{p}_u}}\right)\hbox{--} S{A}_{tissue\mathrm{l} ys}\ast \left({J}_{tissue- lys}\ast {C}_{tissue}\kern0.5em \hbox{--} {J}_{lys- tissue}\ast {C}_{tissue lys}\ \right)\right) $$

25 $$ \frac{d}{dt}{C}_{tissue lys}=\frac{1}{V_{tissue lys}}\left(S{A}_{tissue lys}\ast \left({J}_{tissue- lys}\ast {C}_{tissue}\hbox{--} {J}_{lys- tissue}\ast {C}_{tissue lys}\right)\right) $$

where *C*_arterial_ is the arterial blood concentration, *C*_tissue_ is the non-lysosomal tissue concentration, *C*_tissuelys_ is the tissue specific lysosomal concentration; *Q* is the tissue blood flow rate; *V* is volume of compartment, ${K}_{\mathrm{T}{\mathrm{p}}_{\mathrm{u}}}$ is the tissue-plasma partition coefficient, SA_tissuelys_ is the tissue specific surface area of the lysosome, BP is the blood to plasma ratio, and *f*_*u*_ i﻿s the unbound plasma fraction.

The respiratory tract was divided into four regions by anatomical location and function [29]. The model consisted of the upper airways (nose and larynx) denoted as *UA*, conducting airways (airway branching from generations 0–16) denoted as *CA*, transitional airways (airway branching from generations 17–19) denoted as *TA*, and pulmonary airways (airway branching from generations 20–24) denoted as *PA*. Each respiratory tract region was modeled by further division into six compartments representing the mucus, periciliary layer, cytosol, lysosome, interstitial, and vascular space. Because pulmonary airways do not contain mucus or a periciliary layer, a single compartment representing the surfactant layer was included. Additionally, mucociliary clearance (*K*_*mcc*_) from the conductional, transitional, and upper airway regions to the gastrointestinal tract was included [30].

Using the framework above, PBPK models for mice, rats, and humans were developed. The physiological percent tissue volumes (%*V*) and blood flows (%*Q*) shown in Table III were standard values from Brown et al. [31], and the respiratory tract parameters in Table IV were obtained from Sarangapani et al. [29]. The total cardiac output (*Q*_Total_) for mouse was 14 mL/min, rat was 110 mL/min, and human was 5.2E3 mL/min [29]. The percent tissue volume and percent blood flow rate for remaining compartment were determined by summing up the values for all the tissues and subtracting from 100. The physicochemical parameters for CQ and HCQ were obtained from the literature (Table I) and were used to predict the partitioning coefficients of diprotic bases by Rodger’s method [32]. The plasma-tissue partition coefficient for slow compartment was assumed to be like adipose, and remaining compartment were a fixed value. The metabolism and clearance terms were incorporated for capturing the clearance from liver and kidney [7]. Renal clearance from passive absorption is implemented as the product of unbound drug fraction in blood (*f*_ub_) and glomerular filtration rate (*GFR*). Model parameters were either obtained from the literature [7] or fitted to the experimental data (Table V). Both P-gp transporter variants were modeled in rodents, and only one P-gp transporter variant was set to be active in human PBPK model [26]. A full array of representative equations for PBPK model are described in Eqs. 26–72.

**Table III:** List of PBPK Model Parameters for Different Species

**Species**	**Mouse (22 g)**			**Rat (250 g)**			**Human (70 kg)**				**All**
**Compound**			HCQ			CQ			CQ	HCQ	
**Tissue** [31]	Tissue volume (%V)	Blood flow (%Q)	Partition coefficient (${K}_{T{p}_u}$) [32]	Tissue volume (%V)	Blood flow (%Q)	Partition coefficient (${K}_{T{p}_u}$) [32]	Tissue volume (%V)	Blood flow (%Q)	Partition coefficient (${K}_{T{p}_u}$) [32]	Partition coefficient (${K}_{T{p}_u}$) [32]	Lysosomal volume (%*V*_*lys*_) [7]
Brain	1.7	3.3	26	0.6	2	25	2	11.4	23	26	0.05
Heart	0.5	6.6	101	0.3	5.1	94	0.5	4	91	102	0.1
Kidney	1.7	9.1	312	0.7	14.1	296	0.4	17.5	261	312	0.05
Skin	16.5	5.8	83	19	5.8	79	3.7	5.8	69	83	0.1
GI	4.2	13.1	150	2.7	14.2	143	1.7	17.6	126	151	0.1
Spleen	0.35	1	198	0.2	1	188	0.21	0.5	166	198	0.1
Liver	5.5	2 ^a^	283	3.4	2.1 ^a^	269	2.6	4.6 ^a^	237	284	0.2
Muscle	38.4	15.9	95	40.4	27.8	91	40	19.1	81	96	0.1
Slow ^b^	17.7	12.8	25	14.3	13.8	24	35.7	9.4	21	26	0.1
Remaining			10 ^c^			10 ^c^			10 ^c^	10 ^c^	0.1
Arterial	3.4			3.4			3.4				
Venous	4.0			4.0			4.0				

^*a*^*Hepatic artery blood flow;*^*b*^*bone and fat;*^*c*^*fixed*

**Table IV:** PBPK Modeling Parameters for Respiratory Tract

**Parameter**	**Symbol**	**Unit**	**Mouse (22 g)**	**Rat (250 g)**	**Human (70 kg)**
Mucus thickness	*T*_*UAmuc*_	cm	4E-4^[[29^^]^	9E-4^[[29^^]^	8E-4^[[29^^]^
	*T*_*CAmuc*_	cm	4E-4^[[29^^]^	9E-5^[[29^^]^	4E-4^[[29^^]^
	*T*_*TAmuc*_	cm	4E-4^[[29^^]^	9E-5^[[29^^]^	2E-4^[[29^^]^
	*T*_*PAmuc*_	cm	1E-5 ^[[80^^]^	1E-5 ^[[83^^]^	1E-5 ^a^
Periciliary layer thickness	*T*_*UApcl*_	cm	3E-4 ^b^	3E-4 ^b^	7E-4 ^[[84^^]^
	*T*_*CApcl*_	cm	3E-4 ^b^	3E-4 ^b^	7E-4 ^[[84^^]^
	*T*_*TApcl*_	cm	3E-4 ^b^	3E-4 ^b^	7E-4 ^[[84^^]^
Tissue layer thickness	*T*_*UAtiss*_	cm	1.5E-2 ^[[29^^]^	1.5E-2 ^[[29^^]^	1.5E-2 ^[[29^^]^
	*T*_*CAtiss*_	cm	7.5E-3 ^[[29^^]^	7.5E-3 ^[[29^^]^	7.5E-3 ^[[29^^]^
	*T*_*TAtiss*_	cm	3E-3 ^[[29^^]^	3E-3 ^[[29^^]^	3E-3 ^[[29^^]^
	*T*_*PAtiss*_	cm	4E-4 ^[[29^^]^	3E-4 ^[[29^^]^	5E-4 ^[[29^^]^
Surface area	*SA*_*UA*_	cm^2^	2.7 ^[[29^^]^	13.2 ^[[29^^]^	138 ^[[29^^]^
	*SA*_*CA*_	cm^2^	8.87 ^[[29^^]^	48.3 ^[[29^^]^	2E3 ^[[29^^]^
	*SA*_*TA*_	cm^2^	0.48 ^[[29^^]^	5.5 ^[[29^^]^	6.22E3 ^[[29^^]^
	*SA*_*PA*_	cm^2^	500 ^[[29^^]^	3.4E3 ^[[29^^]^	5.4E5 ^[[29^^]^
Mucociliary clearance (*K*_*mcc*_)	*K*_*UAmcc*_	1/min	0.08 ^c^	0.08 ^c^	0.08 ^c^
	*K*_*CAmcc*_	1/min	3.21E-2 ^[[30^^]^	3.21E-2 ^[[30^^]^	3.21E-2 ^[[30^^]^
	*K*_*TAmcc*_	1/min	4.86E-3 ^[[30^^]^	4.86E-3 ^[[30^^]^	4.86E-3 ^[[30^^]^
Blood flow rate	*Q*_*UA*_	%	1 ^[[29^^]^	1 ^[[29^^]^	0.25 ^[[29^^]^
	*Q*_*CA*_	%	0.5 ^[[29^^]^	2.1 ^[[29^^]^	0.75 ^[[29^^]^
	*Q*_*TA*_	%	0.1 ^[[29^^]^	0.15 ^[[29^^]^	0.67 ^[[29^^]^
	*Q*_*PA*_	%	100	100	100
Lysosomal volume	*V*_*UAlys*_	% ^d^	8% ^e^	8% ^e^	8% ^e^
	*V*_*CAlys*_	% ^d^	8% ^e^	8% ^e^	8% ^e^
	*V*_*TAlys*_	% ^d^	8% ^e^	8% ^e^	8% ^e^
	*V*_*PAlys*_	% ^d^	0.1% ^e^	0.1% ^e^	0.1% ^e^
Interstitial volume	*V*_*inter*_	%	33.6 ^[[81^^]^	33.6 ^[[81^^]^	33.6 ^[[81^^]^
Vascular volume	*V*_*vas*_	%	18.5 ^[[82^^]^	18.5 ^[[82^^]^	18.5 ^[[82^^]^

^*a*^*Same as rodent;*^*b*^*fixed;*^*c*^*internal communication;*^*d*^*% cellular volume;*^*e*^*from in vitro and ex vivo modeling*

**Table V:** PBPK Model Parameters for Absorption, Metabolism, and Clearance of CQ and HCQ (%CV)

**Species**			**Mouse (22 g)**	**Rat (250 g)**	**Human (70 kg)**	
**Parameter**	**Symbol**	**Units**	**HCQ**	**CQ**	**CQ**	**HCQ**
i.p absorption rate	*K*_*ip*_	1/min	7E-3 (6.73) ^a^	2.25E-3 (21.4) ^a^		
Oral absorption rate	*K*_*oral*_	1/min			5E-3 (27.4) ^a^	4.8E-3 (4.3) ^a^
Fraction absorbed	*F*_*oral*_				1 ^[[36^^]^	0.75 ^[[7^^]^
Liver clearance	*CL*_*Liv*_	mL/min			11.1 (81.6) ^a^	12.5 (48.6) ^a^
	*Vmax*_*liv*_	ng/min	1.78E4 (2.97) ^a^	8.03E2 (0.17) ^a^		
	*Km*_*liv*_	ng/mL	1.2E5 ^[[7^^]^	1.14E5 ^c^		
Kidney clearance (*CL*_*kid*_)	*GFR*	mL/min	0.24 ^b^	2.5 ^b^	90 ^b^	90 ^b^
	*Vmax*_*kid*_	ng/mL/min	541 ^[[7^^]^	515 ^c^	515 ^c^	541 ^[[7^^]^
	*Km*_*kid*_	ng/mL	3.36E5 ^[[7^^]^	3.2E5 ^c^	3.2E5 ^c^	3.36E5 ^[[7^^]^
P-gp variant 1	*Vmax*_*pgp*1_	ng/cm^2^/min	4.2E6 ^[[26^^]^	4E6 ^[[26^^]^	9.92E5 ^[[26^^]^	4.2E6 ^d^
	*Km*_*pgp*1_	ng/mL	2.89E3 ^[[26^^]^	2.75E3 ^[[26^^]^	3.68E3 ^[[26^^]^	3.86E3 ^d^
P-gp variant 2	*Vmax*_*pgp*2_	ng/cm^2^/min	5.71E6 ^[[26^^]^	5.44E6 ^[[26^^]^		
	*Km*_*pgp*2_	ng/mL	3.86E3 ^[[26^^]^	3.68E3 ^[[26^^]^		

^*a*^*Optimized;*^*b*^*fixed;*^*c*^*same as rodent;*^*d*^*from in vitro and ex vivo modeling*

#### Upper airway

26 $$ \frac{d}{dt}{C}_{UA muc}=\frac{1}{V_{UA muc}}\left(-\frac{DC}{T_{UA muc}}\ast S{A}_{UA}\ast \left({C}_{UA muc}-{C}_{UA pcl}\right)\right)-{K}_{UA mcc}\ast {C}_{UA muc}+\frac{1}{V_{UA muc}}\left({K}_{CAmcc}\ast {C}_{CAmuc}\ast {V}_{CAmuc}\right) $$

27 $$ \frac{d}{dt}{C}_{UA pcl}=\frac{1}{\ {V}_{UA pcl}}\left(\frac{DC}{T_{UA muc}}\ast S{A}_{UA}\ast \left({C}_{UA muc}-{C}_{UA pcl}\right)+S{A}_{UA}\ast \left({J}_{UA cyt- UAmuc}\ast {C}_{UA cyt}-{J}_{UA muc- UAcyt}\ast {C}_{UA pcl}\right)+\frac{Vma{x}_{pgp1}\ast {\mathrm{SA}}_{\mathrm{UA}}\ast {f}_n\ast {\mathrm{f}}_{\mathrm{u}}\ast {C}_{UA cyt}}{K{m}_{pgp1}+{f}_n\ast {\mathrm{f}}_{\mathrm{u}}\ast {C}_{UA cyt}}+\frac{Vma{x}_{pgp2}\ast {\mathrm{SA}}_{\mathrm{UA}}\ast {f}_n\ast {\mathrm{f}}_{\mathrm{u}}\ast {C}_{UA cyt}}{K{m}_{pgp2}+{f}_n\ast {\mathrm{f}}_{\mathrm{u}}\ast {C}_{UA cyt}}\right)-{K}_{UA mcc}\ast {C}_{UA pcl}\kern0.5em +\frac{1}{V_{UA pcl}}\left({K}_{CAmcc}\ast {C}_{CApcl}\ast {V}_{CApcl}\right) $$

28 $$ \frac{d}{dt}{C}_{UA cyt}=\frac{1}{V_{UA cyt}-{V}_{UA lys}}\left(-S{A}_{UA}\ast \left({J}_{cytUA- mucUA}\ast {C}_{UA cyt}-{J}_{UA muc- UAcyt}\ast {C}_{UA pcl}\right)+S{A}_{UA}\ast \left({J}_{UA inter-\mathrm{U} Acyt}\ast {\mathrm{f}}_{\mathrm{u}}\ast {C}_{UA inter}-{J}_{UA cyt- UAinter}\ast {C}_{UA cyt}\right)-S{A}_{UA lys}\ast \left({J}_{UA cyt- UAlys}\ast {C}_{UA cyt}-{J}_{UA lys- UAcyt}\ast {C}_{UA lys}\right)-\frac{Vma{x}_{pgp1}\ast {\mathrm{SA}}_{\mathrm{UA}}\ast {f}_n\ast {\mathrm{f}}_{\mathrm{u}}\ast {C}_{UA cyt}}{K{m}_{pgp1}+{f}_n\ast {\mathrm{f}}_{\mathrm{u}}\ast {C}_{UA cyt}}-\frac{Vma{x}_{pgp2}\ast {\mathrm{SA}}_{\mathrm{UA}}\ast {f}_n\ast {\mathrm{f}}_{\mathrm{u}}\ast {C}_{UA cyt}}{K{m}_{pgp2}+{f}_n\ast {\mathrm{f}}_{\mathrm{u}}\ast {C}_{UA cyt}}\right) $$

29 $$ \frac{d}{dt}{C}_{UA inter}=\frac{1}{V_{UA inter}}\left({Q}_{UA}\ast \left(\frac{C_{UA vas}}{BP}-{C}_{UA inter}\right)-S{A}_{UA}\ast \left({J}_{UA inter- UAcyt}\ast {\mathrm{f}}_{\mathrm{u}}\ast {C}_{UA inter}-{J}_{UA cyt- UAinter}\ast {C}_{UA cyt}\right)\right) $$

30 $$ \frac{d}{dt}{C}_{U\mathrm{A} lys}=\frac{1}{V_{UAlys}}\ast \left(S{A}_{UAlys}\ast \left({J}_{UAcyt- UAlys}\ast {C}_{UAcyt}-{J}_{lysUA- UAcyt}\ast {C}_{UAlys}\right)\right) $$

31 $$ \frac{d}{dt}{C}_{UA vas}=\frac{1}{V_{UA vas}}\left({Q}_{UA}\ast \left({C}_{Arterial}-{C}_{UA vas}\right)-{Q}_{UA}\ast \left(\frac{C_{UA vas}}{BP}-{C}_{UA inter}\right)\right) $$

#### Conductional airway

32 $$ \frac{d}{dt}{C}_{CA muc}=\frac{1}{V_{CA muc}}\left(-\frac{DC}{T_{CA muc}}\ast S{A}_{CA}\ast \left({C}_{CA muc}-{C}_{CA pcl}\right)\right)-{K}_{CA mcc}\ast {C}_{CA muc}+\frac{1}{V_{CA muc us}}\left({K}_{TAmcc}\ast {C}_{TAmuc}\ast {V}_{TAmuc}\right) $$

33 $$ \frac{d}{dt}{C}_{CA pcl}=\frac{1}{\ {V}_{CA pcl}}\left(\frac{DC}{T_{CA muc}}\ast S{A}_{CA}\ast \left({C}_{CA muc}-{C}_{CA pcl}\right)+\mathrm{S}{A}_{CA}\ast \left({J}_{CA cyt- CAmuc}\ast {C}_{CA cyt}-{J}_{CA muc- CAcyt}\ast {C}_{CA pcl}\right)+\frac{Vma{x}_{pgp1}\ast {SA}_{CA}\ast {f}_n\ast {\mathrm{f}}_{\mathrm{u}}\ast {C}_{CA cyt}}{K{m}_{pgp1}+{f}_n\ast {\mathrm{f}}_{\mathrm{u}}\ast {C}_{CA cyt}}+\frac{Vma{x}_{pgp2}\ast {SA}_{CA}\ast {f}_n\ast {\mathrm{f}}_{\mathrm{u}}\ast {C}_{CA cyt}}{K{m}_{pgp2}+{f}_n\ast {\mathrm{f}}_{\mathrm{u}}\ast {C}_{CA cyt}}\right)-{K}_{CA mcc}\ast {C}_{CA pcl}\kern0.5em +\frac{1}{V_{CA pcl}}\left({K}_{TAmcc}\ast {C}_{TApcl}\ast {V}_{TApcl}\right) $$

34 $$ \frac{d}{dt}{C}_{CA cy t}=\frac{1}{V_{CA cy t}-{V}_{CA lys}}\left(-S{A}_{CA}\ast \left({J}_{CA cy t- CAmuc}\ast {C}_{CA cy t}-{J}_{CA muc- CAcyt}\ast {C}_{CA pcl}\right)+S{A}_{CA}\ast \left({J}_{CA inter- CAcyt}\ast {\mathrm{f}}_{\mathrm{u}}\ast {C}_{CA inter}-{J}_{CA cy t- CAinter}\ast {C}_{CA cy t}\right)-S{A}_{CA lys}\ast \left({J}_{CA cy t- CAlys}\ast {C}_{CA cy t}-{J}_{CA lys- CAcyt}\ast {C}_{CA lys}\right)-\frac{Vma{x}_{pgp1}\ast {SA}_{CA}\ast {f}_n\ast {\mathrm{f}}_{\mathrm{u}}\ast {C}_{CA cy t}}{K{m}_{pgp1}+{f}_n\ast {\mathrm{f}}_{\mathrm{u}}\ast {C}_{CA cy t}}-\frac{Vma{x}_{pgp2}\ast {SA}_{CA}\ast {f}_n\ast {\mathrm{f}}_{\mathrm{u}}\ast {C}_{CA cy\mathrm{t}}}{K{m}_{pgp2}+{f}_n\ast {\mathrm{f}}_{\mathrm{u}}\ast {C}_{CA cy t}}\right) $$

35 $$ \frac{d}{dt}{C}_{CA inter}=\frac{1}{V_{CA inter}}\left({Q}_{CA}\ast \left(\frac{C_{CA vas}}{BP}-{C}_{CA inter}\right)-S{A}_{CA}\ast \left({J}_{CA inter- CAcyt}\ast {\mathrm{f}}_{\mathrm{u}}\ast {C}_{CA inter}-{J}_{CA cyt- CAinter}\ast {C}_{CA cyt}\right)\right) $$

36 $$ \frac{d}{dt}{C}_{CAlys}=\frac{1}{V_{CAlys}}\ast \left(S{A}_{CAlys}\ast \left({J}_{CAcyt- CAlys}\ast {C}_{CAcyt}-{J}_{CAlys- CAcyt}\ast {C}_{CAlys}\right)\right) $$

37 $$ \frac{d}{dt}{C}_{CA vas}=\frac{1}{V_{CA vas}}\left({Q}_{CA}\ast \left({C}_{Arterial}-{C}_{CA vas}\right)-{Q}_{CA}\ast \left(\frac{C_{CA vas}}{BP}-{C}_{CA inter}\right)\right) $$

#### Transitional airway

38 $$ \frac{d}{dt}{C}_{TA muc}=\frac{1}{V_{TA muc}}\left(-\frac{DC}{T_{TA muc}}\ast S{A}_{TA}\ast \left({C}_{TA muc}-{C}_{TA pcl}\right)\right)-{K}_{TA mcc}\ast {C}_{TA muc} $$

39 $$ \frac{d}{dt}{C}_{TA pcl}=\frac{1}{\ {V}_{TA pcl}}\left(\frac{DC}{T_{TA muc}}\ast S{A}_{TA}\ast \left({C}_{TA muc}-{C}_{TA pcl}\right)+S{A}_{TA}\ast \left({J}_{TA cyt- TAmuc}\ast {C}_{TA cyt}-{J}_{TA muc- TAcytTA}\ast {C}_{\mathrm{T} Apcl}\right)+\frac{Vma{x}_{pgp1}\ast S{A}_{TA}\ast {f}_n\ast {\mathrm{f}}_{\mathrm{u}}\ast {C}_{TA cyt}}{K{m}_{pgp1}+{f}_n\ast {\mathrm{f}}_{\mathrm{u}}\ast {C}_{TA cyt}}+\frac{Vma{x}_{pgp2}\ast S{A}_{TA}\ast {f}_n\ast {\mathrm{f}}_{\mathrm{u}}\ast {C}_{TA cyt}}{K{m}_{pgp2}+{f}_n\ast {\mathrm{f}}_{\mathrm{u}}\ast {C}_{TA cyt}}\right)-{K}_{TA mcc}\ast {C}_{TA pcl} $$

40 $$ \frac{d}{dt}{C}_{TA cyt}=\frac{1}{V_{TA cyt}-{V}_{TA lys}}\left(-S{A}_{TA}\ast \left({J}_{TA cyt- TAmuc}\ast {C}_{TA cyt}-{J}_{TA muc- TAcyt}\ast {C}_{TA pcl}\right)+S{A}_{TA}\ast \left({J}_{TA inter- TAcyt}\ast {\mathrm{f}}_{\mathrm{u}}\ast {C}_{TA inter}-{J}_{TA cyt- TAinter}\ast {C}_{TA cyt}\right)-S{A}_{TA lys}\ast \left({J}_{TA cyt- TAlys}\ast {C}_{TA cyt}-{J}_{TA lys- TAcyt}\ast {C}_{T\mathrm{A} lys}\right)-\frac{Vma{x}_{pgp1}\ast S{A}_{TA}\ast {f}_n\ast {\mathrm{f}}_{\mathrm{u}}\ast {C}_{TA cyt}}{K{m}_{pgp1}+{f}_n\ast {\mathrm{f}}_{\mathrm{u}}\ast {C}_{TA cyt}}-\frac{Vma{x}_{pgp2}\ast S{A}_{TA}\ast {f}_n\ast {\mathrm{f}}_{\mathrm{u}}\ast {C}_{TA cyt}}{K{m}_{pgp2}+{f}_n\ast {\mathrm{f}}_{\mathrm{u}}\ast {C}_{TA cyt}}\right) $$

41 $$ \frac{d}{dt}{C}_{TA inter}=\frac{1}{V_{TA inter}}\left({Q}_{TA}\ast \left(\frac{C_{TA vas}}{BP}-{C}_{TA inter}\right)-S{\mathrm{A}}_{TA}\ast \left({J}_{TA inter- TAcyt}\ast {\mathrm{f}}_{\mathrm{u}}\ast {C}_{TA inter}-{J}_{TA cyt- TAinter}\ast {C}_{TA cyt}\right)\right) $$

42 $$ \frac{d}{dt}{C}_{TAlys}=\frac{1}{V_{TAlys}}\ast \left(S{A}_{TAlys}\ast \left({J}_{TAcyt- TAlys}\ast {C}_{TAcyt}-{J}_{TAlys- TAcyt}\ast {C}_{TAlys}\right)\right) $$

43 $$ \frac{d}{dt}{C}_{TA vas}=\frac{1}{V_{TA\mathrm{v} as}}\left({Q}_{TA}\ast \left({C}_{Arterial}-{C}_{TA vas}\right)-{Q}_{TA}\ast \left(\frac{C_{TA vas}}{BP}-{C}_{TA inter}\right)\right) $$

#### Pulmonary alveolar region

44 $$ \frac{d}{dt}{C}_{PA muc}=\frac{1}{V_{PA muc us}}\left(S{A}_{PA}\ast \left({J}_{PA cyt- PAmuc}\ast {C}_{PA cyt}-{J}_{PA muc- PAcyt}\ast {C}_{PA muc}\right)+\frac{Vma{x}_{pgp1}\ast S{A}_{PA}\ast {f}_n\ast {\mathrm{f}}_{\mathrm{u}}\ast {C}_{PA cyt}}{K{m}_{pgp1}+{f}_n\ast {\mathrm{f}}_{\mathrm{u}}\ast {C}_{PA cyt}}+\frac{Vma{x}_{pgp2}\ast S{A}_{PA}\ast {f}_n\ast {\mathrm{f}}_{\mathrm{u}}\ast {C}_{PA cyt}}{K{m}_{pgp2}+{f}_n\ast {\mathrm{f}}_{\mathrm{u}}\ast {C}_{PA cyt}}\right) $$

45 $$ \frac{d}{dt}{C}_{PA cyt}=\frac{1}{V_{PA cyt}-{V}_{PA lys}}\left(\operatorname{}S{A}_{PA}\ast \left({J}_{PA cyt- PAmuc}\ast {C}_{PA cyt}-{J}_{PA muc- PAcyt}\ast {C}_{PA muc}\right)+S{A}_{PA}\ast \left({J}_{PA inter- PAcyt}\ast {\mathrm{f}}_{\mathrm{u}}\ast {C}_{PA inter}-{J}_{PA cyt- PAinter}\ast {C}_{PA cyt}\right)-S{A}_{PA lys}\ast \left({J}_{PA cyt- PAlys}\ast {C}_{PA cyt}-{J}_{PA lys- PAcyt}\ast {C}_{PA lys}\right)-\frac{Vma{x}_{pgp1}\ast S{A}_{PA}\ast {f}_n\ast {\mathrm{f}}_{\mathrm{u}}\ast {C}_{PA cyt}}{K{m}_{pgp1}+{f}_n\ast {\mathrm{f}}_{\mathrm{u}}\ast {C}_{PA cyt}}-\frac{Vma{x}_{pgp2}\ast S{A}_{PA}\ast {f}_n\ast {\mathrm{f}}_{\mathrm{u}}\ast {C}_{PA cyt}}{K{m}_{pgp2}+{f}_n\ast {\mathrm{f}}_{\mathrm{u}}\ast {C}_{PA cyt}}\right) $$

46 $$ \frac{d}{dt}{C}_{PA inter}=\frac{1}{V_{PA inter}}\left({Q}_{Total}\ast \left(\frac{C_{PA vas}}{BP}-{C}_{PA inter}\right)-S{A}_{PA}\ast \left({J}_{PA inter- PAcyt}\ast {\mathrm{f}}_{\mathrm{u}}\ast {C}_{PA inter}-{J}_{PA cyt- PAinter}\ast {C}_{PA cyt}\right)\right) $$

47 $$ \frac{d}{dt}{C}_{PAlys}=\frac{1}{V_{PAlys}}\ast \left(S{A}_{PAlys}\ast \left({J}_{PAcyt- PAlys}\ast {C}_{PAcyt}-{J}_{PAlys- PAcyt}\ast {C}_{PAlys}\right)\right) $$

48 $$ \frac{d}{dt}{C}_{PAvas}=\frac{1}{V_{PAvas}}\left({Q}_{Total}\ast \left({C}_{Venous}-{C}_{PAvas}\right)-{Q}_{Total}\ast \left(\frac{C_{PAvas}}{BP}-{C}_{PAinter}\right)\right) $$

#### Arterial

49 $$ \frac{d}{dt}{C}_{Arterial}=\frac{1}{V_{Arterial}}\ast \left({Q}_{Total}\ast \left({C}_{PAvas}-{C}_{Arterial}\right)- GFR\ast {f}_{ub}\ast {C}_{Arterial}\right) $$

#### Venous

50 $$ \frac{d}{dt}{C}_{Venous}=\frac{1}{V_{Venous}}\ast \left({Q}_{Brain}\ast \frac{BP\ast {C}_{Brain}}{fu\ast {K}_{T{p}_u Brain}}+{Q}_{Heart}\ast \frac{BP\ast {C}_{Heart}}{fu\ast {K}_{T{p}_u Heart}}+{Q}_{Kidney}\ast \frac{BP\ast {C}_{Kidney}}{fu\ast {K}_{T{p}_u kidney}}+\left({Q}_{Liver}+{Q}_{GI}+{Q}_{Spleen}\right)\ast \frac{BP\ast {C}_{Liver}}{fu\ast {K}_{T{p}_u liver}}+{Q}_{Muscle}\ast \frac{BP\ast {C}_{Muscle}}{fu\ast {K}_{T{p}_u Muscle}}+{Q}_{Skin}\ast \frac{BP\ast {C}_{Skin}}{fu\ast {K}_{T{p}_u Skin}}+{Q}_{Slow}\ast \frac{BP\ast {C}_{Slow}}{fu\ast {K}_{T{p}_u Slow}}+{Q}_{Remaining}\ast \frac{BP\ast {C}_{Remaining}}{fu\ast {K}_{T{p}_u Remaining}}+{Q}_{UA}\ast {C}_{UA vas}+{Q}_{CA}\ast {C}_{CA vas}+{Q}_{TA}\ast {C}_{TA vas}-{Q}_{Total}\ast {C}_{Venous}\right) $$

#### Brain

51 $$ \frac{d}{dt}{C}_{Brain}=\frac{1}{V_{Brain}-{V}_{Brain lys}}\ast \left({Q}_{Brain}\ast \left({C}_{Arterial}-\frac{BP\ast {C}_{Brain}}{fu\ast {K}_{T{p}_u Brain}}\right)-S{A}_{Brain lys}\ast \left({J}_{Brain- lys}\ast {C}_{Brain}-{J}_{lys- Brain}\ast {C}_{Brain lys}\right)\right) $$

52 $$ \frac{d}{dt}{C}_{Brain lys}=\frac{1}{V_{Brain lys}}\ast \left(S{A}_{Brain lys}\ast \left({J}_{Brain- lys}\ast {C}_{Brain}-{J}_{lys- Brain}\ast {C}_{Brain lys}\right)\right) $$

#### Heart

53 $$ \frac{d}{dt}{C}_{Heart}=\frac{1}{V_{Heart}-{V}_{Heart lys}}\ast \left({Q}_{Heart}\ast \left({C}_{Arterial}-\frac{BP\ast {C}_{Heart}}{fu\ast {K}_{T{p}_u Heart}}\right)-S{A}_{Heart lys}\ast \left({J}_{Heart- lys}\ast {C}_{Heart}-{J}_{lys- Heart}\ast {C}_{Heart lys}\right)\right) $$

54 $$ \frac{d}{dt}{C}_{Heart lys}=\frac{1}{V_{Heart lys}}\ast \left(S{A}_{Heart lys}\ast \left({J}_{Heart- lys}\ast {C}_{Heart}-{J}_{lys- Heart}\ast {C}_{Heart lys}\right)\right) $$

#### Kidney

55 $$ \frac{d}{dt}{C}_{Kidney}=\frac{1}{V_{Kidney}-{V}_{Kidney lys}}\ast \left({Q}_{Kidney}\ast \left({C}_{Arterial}-\frac{BP\ast {C}_{Kidney}}{fu\ast {K}_{T{p}_u kidney}}\right)-S{A}_{Kidney lys}\ast \left({J}_{Kidney- lys}\ast {C}_{Kidney}-{J}_{lys- Kidney}\ast {C}_{Kidney lys}\right)-\frac{\left({V}_{Kidney}-{V}_{Kidney lys}\right)\ast Vma{x}_{kid}\ast fu\ast {C}_{Kidney}}{K{m}_{kid}+ fu\ast {C}_{Kidney}}\right) $$

56 $$ \frac{d}{dt}{C}_{Kidney lys}=\frac{1}{V_{Kidney lys}}\ast {SA}_{Kidney lys}\ast \left({J}_{Kidney- lys}\ast {C}_{Kidney}-{\mathrm{J}}_{lys- Kidney}\ast {C}_{Kidney lys}\right) $$

#### Liver (Eq. 56 for humans)

57 $$ \frac{d}{dt}{C}_{Liv er}=\frac{1}{V_{Liv er}-{V}_{Liv er lys}}\ast \left({Q}_{Liv er}\ast {C}_{Arterial}+{Q}_{GI}\ast \frac{BP\ast {C}_{GI}}{fu\ast {K}_{T{p}_u GI}}+{Q}_{Spleen}\ast \frac{BP\ast {C}_{Spleen}}{fu\ast {K}_{T{p}_u spleen}}-\left({\mathrm{Q}}_{Liv er}+{Q}_{GI}+{Q}_{Spleen}\right)\ast \frac{BP\ast {C}_{Liv er}}{fu\ast {K}_{T{p}_u liver}}-S{A}_{Liv er lys}\ast \left({J}_{liver- lys}\ast {C}_{Liv er}-{J}_{lys- Liver}\ast {C}_{Liv er lys}\right)-C{L}_{Liv}\ast fu\ast {C}_{Liv er}\right) $$

#### Liver (Eq. 58 for rodents)

58 $$ \frac{d}{dt}{C}_{Liver}=\frac{1}{V_{Liver}-{V}_{Liver lys}}\ast \left({Q}_{Liver}\ast {C}_{Arterial}+{Q}_{GI}\ast \frac{BP\ast {C}_{GI}}{fu\ast {K}_{T{p}_u GI}}+{Q}_{Spleen}\ast \frac{BP\ast {C}_{Spleen}}{fu\ast {K}_{T{p}_u spleen}}-\left({Q}_{Liver}+{Q}_{GI}+{Q}_{Spleen}\right)\ast \frac{BP\ast {C}_{Liver}}{fu\ast {K}_{T{p}_u liver}}-S{A}_{Liver lys}\ast \left({J}_{liver- lys}\ast {C}_{Liver}-{J}_{lys- Liver}\ast {C}_{Liver lys}\right)-\frac{\left({V}_{Liver}-{V}_{Liver lys}\right)\ast {\mathrm{Vmax}}_{\mathrm{liv}}\ast {\mathrm{f}}_{\mathrm{u}}\ast {\mathrm{C}}_{\mathrm{Liver}}}{{\mathrm{Km}}_{\mathrm{liv}}+{\mathrm{f}}_{\mathrm{u}}\ast {\mathrm{C}}_{\mathrm{Liver}}}\right) $$

59 $$ \frac{d}{dt}{C}_{Liver lys}=\frac{1}{V_{Liver lys}}\ast {SA}_{Liver lys}\ast \left({J}_{Liver- lys}\ast {C}_{Liver}-{J}_{lys- Liv\mathrm{e}r}\ast {C}_{Liver lys}\right) $$

#### Gastrointestinal (GI)

60 $$ \frac{d}{dt} GUT=- KA\ast GUT+{K}_{UAmcc}\ast {C}_{UAmuc}\ast {V}_{UAmuc}+{K}_{UAmcc}\ast {C}_{UApcl}\ast {V}_{UApcl} $$

61 $$ \frac{d}{dt}{C}_{GI}=\frac{1}{V_{GI}-{V}_{GI lys}}\ast \left({Q}_{GI}\ast \left({C}_{Arterial}-\frac{BP\ast {C}_{GI}}{fu\ast {K}_{T{p}_u GI}}\right)-S{A}_{GI\mathrm{l} ys}\ast \left({J}_{GI- lys}\ast {C}_{GI}-{J}_{lys- GI}\ast {C}_{GI lys}\right)+ KA\ast GUT\right) $$

62 $$ \frac{d}{dt}{C}_{GI lys}=\frac{1}{V_{GI lys}}\ast \left(S{A}_{GI lys}\ast \left({J}_{GI- lys}\ast {C}_{GI}-{J}_{lys- GI}\ast {C}_{GI lys}\right)\right) $$

#### Spleen

63 $$ \frac{d}{dt}{C}_{Spleen}=\frac{1}{V_{Spleen}-{V}_{Spleen lys}}\ast \left({Q}_{Spleen}\ast \left({C}_{Arterial}-\frac{BP\ast {C}_{Spleen}}{fu\ast {K}_{T{p}_u Spleen}}\right)-S{A}_{Spleen lys}\ast \left({J}_{Spleen- lys}\ast {C}_{Spleen}-{J}_{lys- Spleen}\ast {C}_{Spleen lys}\right)\right) $$

64 $$ \frac{d}{dt}{C}_{Spleen lys}=\frac{1}{V_{Spleen lys}}\ast \left(S{A}_{Spleen lys}\ast \left({J}_{Spleen- lys}\ast {C}_{Spleen}-{J}_{lys- Spleen}\ast {C}_{Spleen lys}\right)\right) $$

#### Muscle

65 $$ \frac{d}{dt}{C}_{Muscle}=\frac{1}{V_{Muscle}-{V}_{Muscle lys}}\ast \left({Q}_{Muscle}\ast \left({C}_{Arterial}-\frac{BP\ast {C}_{Muscle}}{fu\ast {K}_{T{p}_u Muscle}}\right)-S{A}_{Muscle lys}\ast \left({J}_{Muscle- lys}\ast {C}_{Muscle}-{J}_{lys- Muscle}\ast {C}_{Muscle lys}\right)\right) $$

66 $$ \frac{d}{dt}{C}_{Muscle lys}=\frac{1}{V_{Muscle lys}}\ast \left(S{A}_{Muscle lys}\ast \left({J}_{Muscle- lys}\ast {C}_{Muscle}-{J}_{lys- Muscle}\ast {C}_{Muscle lys}\right)\right) $$

#### Skin

67 $$ \frac{d}{dt}{C}_{S\mathrm{k} in}=\frac{1}{V_{Skin}-{V}_{Skin lys}}\ast \left({Q}_{Skin}\ast \left({C}_{Arterial}-\frac{BP\ast {C}_{Skin}}{fu\ast {K}_{T{p}_u Skin}}\right)-S{A}_{Skin lys}\ast \left({J}_{Skin- lys}\ast {C}_{Skin}-{J}_{lys- Skin}\ast {C}_{Skin lys}\right)\right) $$

68 $$ \frac{d}{dt}{C}_{Skin lys}=\frac{1}{V_{Skin lys}}\ast \left(S{A}_{Skin lys}\ast \left({J}_{S\mathrm{k} in- lys}\ast {C}_{Skin}-{J}_{lys- Skin}\ast {C}_{Skin lys}\right)\right) $$

#### Slow (bone and fat)

69 $$ \frac{d}{dt}{C}_{Slow}=\frac{1}{V_{Slow}-{V}_{Slow}}\ast \left({Q}_{Slow}\ast \left({C}_{Arterial}-\frac{BP\ast {C}_{Slow}}{fu\ast {K}_{T{p}_u Slow}}\right)-S{A}_{Slow lys}\ast \left({J}_{Slow- lys}\ast {C}_{Slow}-{J}_{lys- Sl\mathrm{o}w}\ast {C}_{Slow lys}\right)\right) $$

70 $$ \frac{d}{dt}{C}_{Slow lys}=\frac{1}{V_{Slow lys}}\ast \left(S{A}_{Slow lys}\ast \left({J}_{Slow- lys}\ast {C}_{Slow}-{J}_{lys- Slow}\ast {C}_{Slow lys}\right)\right) $$

#### Remaining

71 $$ \frac{d}{dt}{C}_{Remaining}=\frac{1}{V_{Remaining}-{V}_{Remaining}}\ast \left({Q}_{Remaining}\ast \left({C}_{Arterial}-\frac{BP\ast {C}_{Remaining}}{fu\ast {K}_{T{p}_u Remaining}}\right)-S{A}_{Remaining lys}\ast \left({J}_{Remaining- lys}\ast {C}_{Remaining}-{J}_{lys- Remaining}\ast {C}_{Remaining lys}\right)\right) $$

72 $$ \frac{d}{dt}{C}_{Remaining lys}=\frac{1}{V_{Remaining lys}}\ast \left(S{A}_{Remaining lys}\ast \left({J}_{Remaining- lys}\ast {C}_{Remaining}-{J}_{lys- Remaining}\ast {C}_{Remaining lys}\right)\right) $$

The PBPK model was constructed and simulated in R version 3.5.1 using packages such as *mrgsolve* [33] for describing the PBPK framework, *GenSA* [34] for model optimization to minimize residual sum of squares for plasma or blood concentrations, and *ggplot2* [35] for generating plots. The blood, plasma, and tissue time concentrations from the literature were graphed using WebPlotDigitizer [36]. The model code is provided in supplemental material.

## Results

### Formulation and Characterization of CQ and HCQ Aerosols

**Figure 2:**
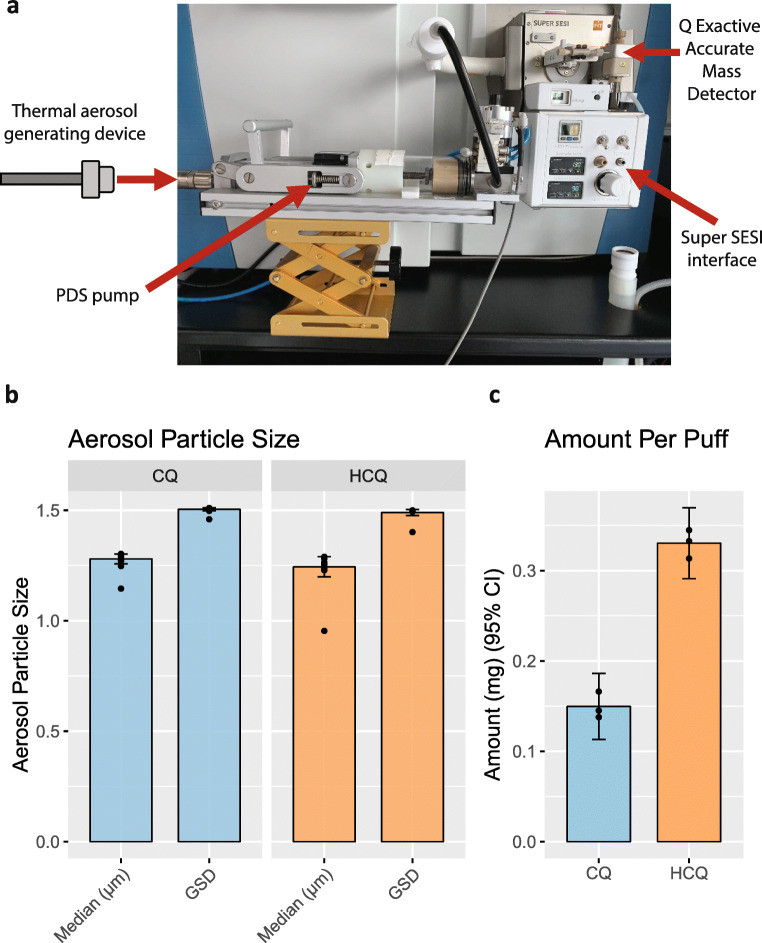
Characterization of chloroquine (CQ) and hydroxychloroquine (HCQ) aerosols produced by thermal aerosolization. **a** Instrumental setup for measuring the aerosolization of chloroquine and hydroxychloroquine by using a thermal aerosol-generating device coupled to a programmable dual syringe (PDS) pump and connected to SUPER SESI platform interfaced with a Q Exactive HF high-resolution accurate mass spectrometer. **b** Particle size measurements (*N* = 30). **c** Amount of CQ and HCQ transferred per 55 mL puff volume from the device (*N* = 3). Data are presented as mean (bars) of technical replicates (dots) and 95% confidence intervals (error bar). SESI, secondary electrospray ionization; GSD, geometric standard deviation

Aerosol formulations containing concentrations up to 40 mg/mL of CQ and 100 mg/mL of HCQ were prepared using propylene glycol as solvent. The solubility of CQ and HCQ in propylene glycol was assessed by LC-HR-MS using an external calibration curve at concentrations shown in Table SI. The accuracy values were high and determined by comparing experimentally measured concentrations against theoretical concentrations for solubility. Thermal aerosol-generating device filled with formulations containing 40 mg/mL CQ or 100 mg/mL HCQ in propylene glycol requires activation by the user puff followed by inhalation. We used a human-relevant regimen of 55 mL delivered in a 3-s puff duration and 30-s intervals using a PDS pump to generate aerosol for inhalation (Figure 2a and Figure S2). Aerosol particles had a median aerodynamic diameter of 1.3 μm and a geometric standard deviation of 1.5 (Figure 2b). We assessed the mass transfer to measure the amount of CQ and HCQ in the aerosols emitted from the device. A total of 30 puffs from 40 μg/mL CQ and 100 μg/mL HCQ liquid formulation using were collected on filter pads, and the amount per puff was 0.15 mg (9.84 %CV) for CQ and 0.33 mg (4.78 %CV) for HCQ (Figure 2c). The device holds 2 mL of formulated liquid delivering 400 puffs leading to an estimated theoretical transfer efficiency of 78% for CQ and 100% for HCQ.

### *In Vitro* Toxicity Assessment of CQ and HCQ Aerosols with 3D Organotypic HBEC

**Figure 3:**
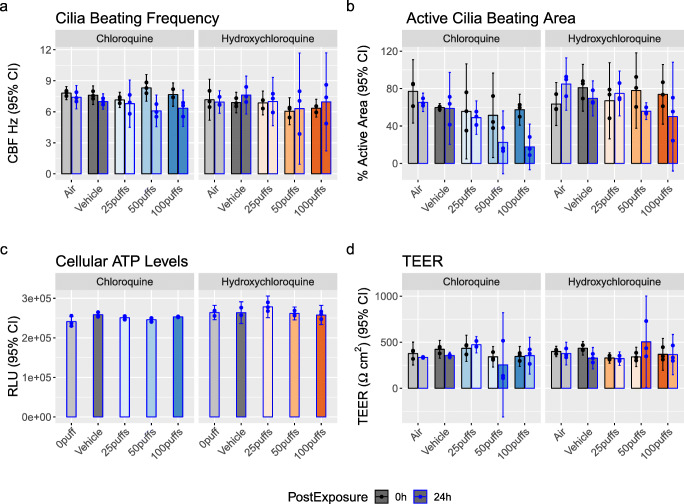
Effects of exposure on **a** ciliary beating frequency (CBF), **b** active ciliary beating area, **c** cellular ATP levels, and **d** transepithelial electrical resistance (TEER) in three-dimensional organotypic human bronchial airway cells. Data shown before (black bars and dots) and after 24-h (blue bars and dots) exposure to various concentrations of chloroquine and hydroxychloroquine. Data are presented as mean (bars) of three technical replicates (dots) and 95% confidence intervals (error bars). RLU, relative luminescence units

To evaluate the safety of CQ and HCQ aerosols in human airways, we exposed 3D organotypic HBEC at the ALI to 25, 50, and 100 puffs of aerosol containing CQ or HCQ. The deposited doses are provided in Table SII. The 3D organotypic HBEC were evaluated at 0 h (pre-exposure) and 24-h post-exposure by measuring ciliary beating frequency, active ciliary area, and TEER. The ciliary beating frequency of unexposed tissues ranged from 6 to 8 Hz for air and vehicle controls. Twenty-four-hourpost-exposure to CQ or HCQ, the ciliary beating frequency remained in this range independent of the number of puffs (Figure 3a). The active ciliary area corresponds to the percentage of the tissue surface where ciliary beating was detected and showed no significant effect for 25 puffs of CQ and HCQ. Twenty-four-hourpost-exposure to CQ, we observed a concentration-dependent effect with a 57% decrease in active ciliary area after exposure to 50 puffs and a 70% decrease after exposure to 100 puffs from pre-exposure values (Figure 3b). HCQ exposure did not cause significant changes in active ciliary area (Figure 3b).

Cell viability was then assessed by measuring the ATP content of the tissues 24-h post-exposure to CQ or HCQ aerosol. For both the drugs and all concentrations tested, ATP tissue content was similar to the ATP content measured in tissues exposed to air or to the vehicle (Figure 3c). TEER was measured to evaluate the human bronchial epithelium tightness; electrical resistance ranged from 350 to 500 Ω × cm^2^ before and after exposure under all conditions tested (Figure 3d). An exposure to 50 puffs of CQ yielded very low TEER values for two replicates 24-h post-exposure and could be an outlier as the third replicate had a TEER value similar to the value obtained before exposure. Moreover, an exposure to 100 puffs of CQ and HCQ aerosols did not lower the TEER value and exhibited no overall effect.

### Modeling *In Vitro* 3D Organotypic HBEC and IPML Kinetics

**Figure 4:**
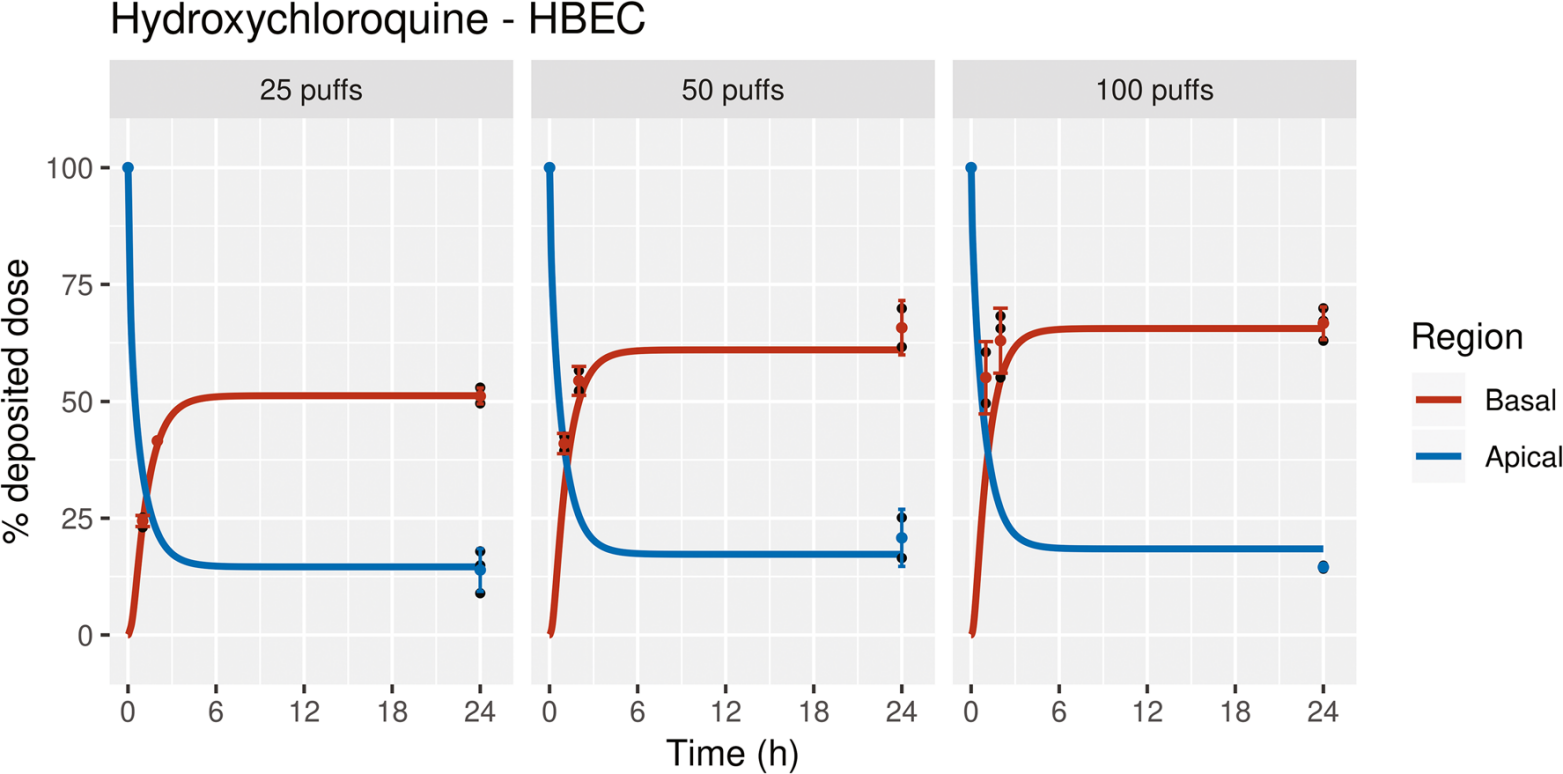
Simulated transport kinetics of hydroxychloroquine transport in human bronchial epithelial cells (HBEC) at the air-liquid interface exposed to 25, 50, or 100 puffs of hydroxychloroquine aerosol. The amount of deposited HCQ for 25 puffs was 7.99 μg, 50 puffs was 15.9 μg, and 100 puffs was 28.3 μg. Black dots denote individual experimental values, colored dots denote the experimental mean for three technical replicates, error bars represent standard deviation, and lines represent model prediction

The transport kinetics of HCQ across 3D organotypic HBEC were measured at different time intervals. A 24-h measurement of apical and basal concentrations was included to mimic earlier *in vitro* toxicity measurements. The amount of aerosolized HCQ deposited in cell-free controls was 7.99 μg for 25 puffs, 15.9 μg for 50 puffs, and 28.3 μg for 100 puffs (Table SII). An *in vitro* kinetic model was developed using parameters shown in Tables I and II. As the *in vitro* airway surface liquid in human bronchial epithelial cells was slightly acidic, a pH of 7.2 was set to the apical mucus and periciliary layer compartments and cytosolic pH as 7.2 [37, 38]. The intracellular acidic compartments such as lysosomes in human bronchial epithelial cells were set to be 8% of total cellular volume as reported by Ufuk et al. [39]. To evaluate *in vitro* model structure, a model-driven hypothesis testing was performed as described in supplementary information. As described by Weiss et al. [40], we included P-gp efflux transporter to simulate HCQ kinetics. Price et al. have also evaluated and reported the P-gp transporter kinetics for CQ in an *ex vivo* IPML [26]. The *in vitro* HBEC model predictions of apical and basal concentrations were improved upon including P-gp efflux transporter kinetics using a Michaelis–Menten formalism (Figure 4). The apically deposited HCQ reached equilibrium across different compartments, and after a 24-h post-exposure, 51.3%, 60.9%, and 65.6% of the deposited dose were transferred to the basal compartment, while 14.6%, 17.3%, and 18.4% of the deposited dose remained in the apical compartment for 25, 50, and 100 puffs, respectively (Figure 4). The difference in fractions of drug in the apical and basal compartments is attributable to pH-dependent lysosomal trapping of HCQ.

**Figure 5:**
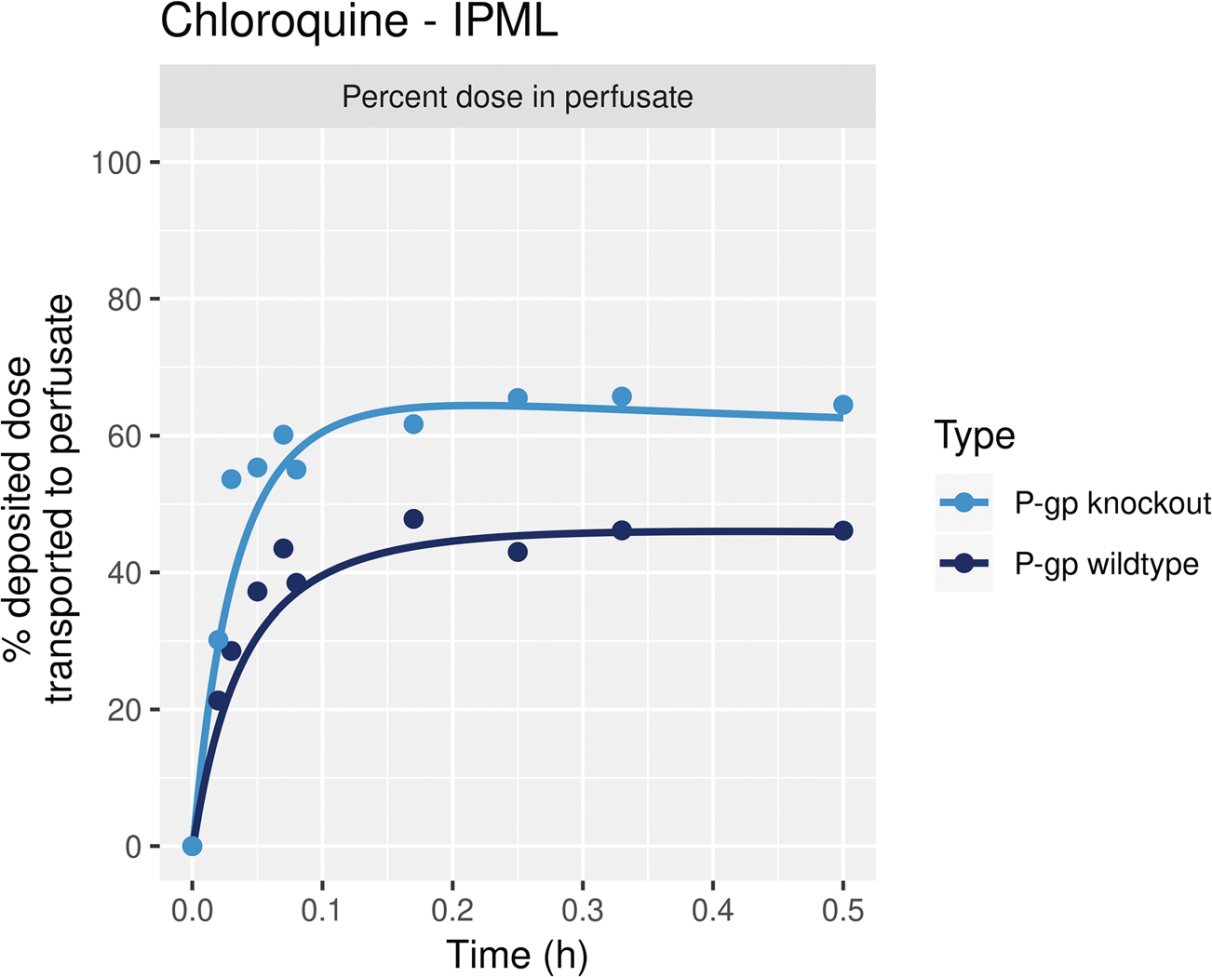
Percent deposited dose of chloroquine transported across pulmonary airway to perfusate in P-gp wild-type transporter expressed isolated perfused mouse lung (IPML) and P-gp knockout IPML. Dots denote the experimental data from Price et al. [26], and lines represent model prediction

The experimental data and parameters used to model the transport kinetics of aerosolized CQ in *ex vivo* IPML were obtained from Price et al. [26]. CQ transport kinetics were measured by Price et al. in P-gp wild-type IPML and P-gp knockout IPML. The P-gp knockout IPML experimental model was developed by disrupting the mdr gene using a neomycin resistance cassette [26]. Price et al. reported that aerosol-deposited fraction in the IMPL was 80% of the delivered amount. In IPML kinetic model, the physiologically relevant pH of airway surface liquid and cytosol was set at 7.0 [41, 42]. The IPML kinetic model was simulated with and without the P-gp efflux transporter kinetics using the reported values [26]. In P-gp knockout IPML, 62.6% of the deposited concentration was transported across the pulmonary barrier in 30 min (Figure 5). In contrast, in a P-gp wild-type IPML, the percentage of drug in the perfusate media was 16.69% lower than that of the P-gp knockout IPML indicating a significant accumulation in airway surface fluid.

### PBPK Model Qualification

**Figure 6:**
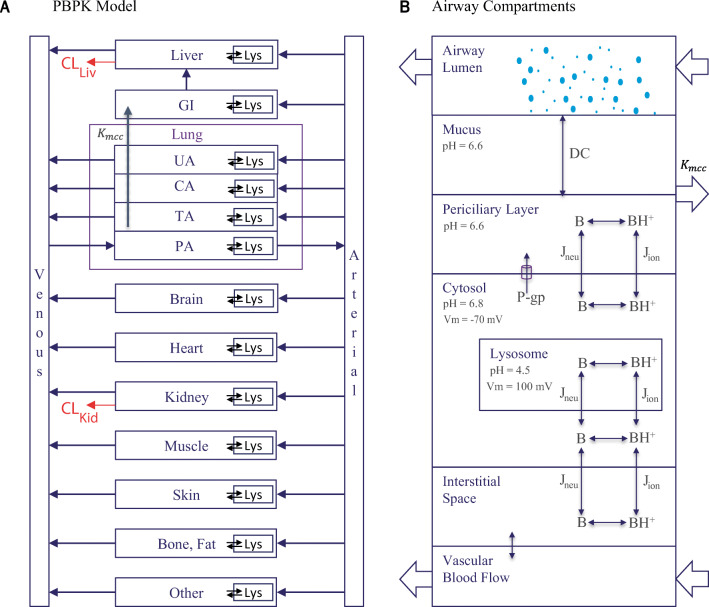
Schematic of **a** inhalation physiologically based pharmacokinetic (PBPK) model for chloroquine and hydroxychloroquine with a **b** detailed airway tract model. GI, gastrointestinal tract; Lys, lysosomes, *K*_*mcc*_, rate of mucociliary clearance; UA, upper airway; CA, conducting airway; TA, transitional airway; PA, pulmonary airway; CL_Liv_, clearance from liver; CL_Kid_, clearance from kidney

**Figure 7:**
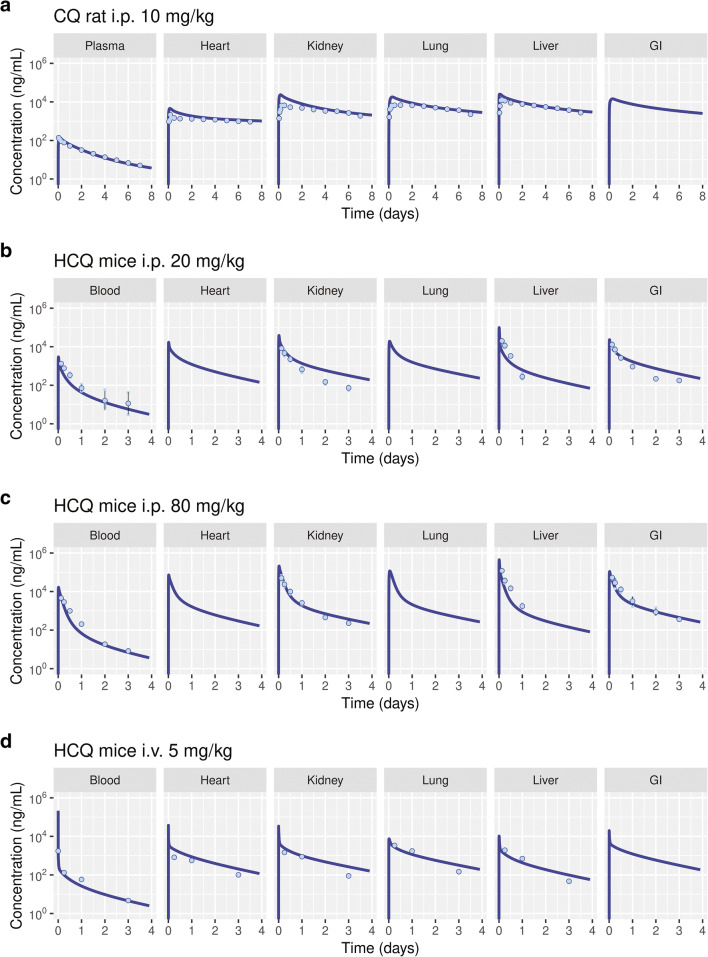
Physiologically based pharmacokinetic model-predicted concentration-time profiles in plasma, blood, heart, kidney, lung, liver, and gastrointestinal tract (GI). Chloroquine (CQ) pharmacokinetics was simulated in rats (**a**), and hydroxychloroquine (HCQ) pharmacokinetics was simulated in mice (**b**–**d**). The experimental data (dots) were obtained from Adelusi et al.[45], Collins et al. [7], and Chhonker et al. [76]

**Figure 8:**
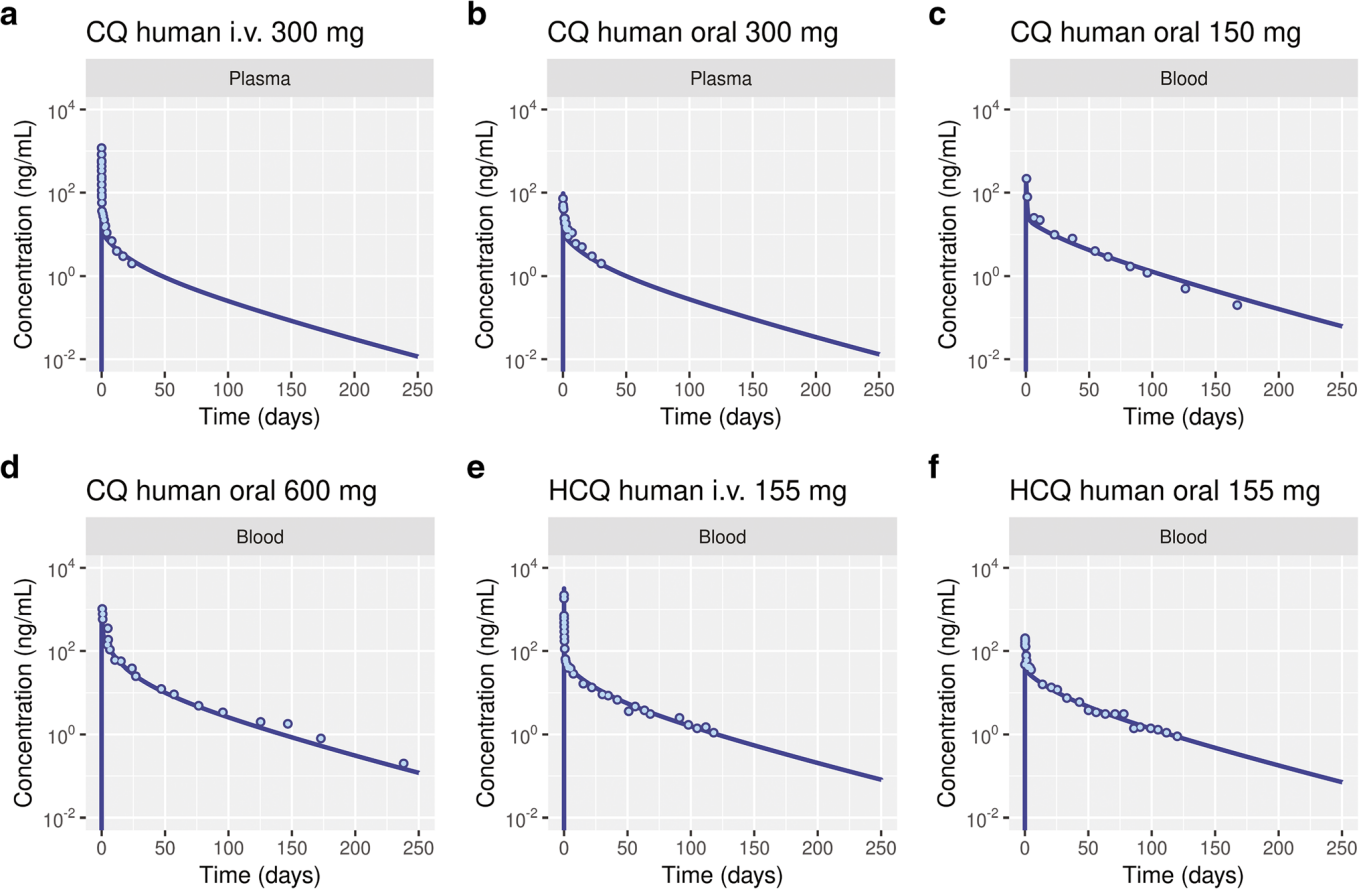
Physiologically based pharmacokinetic model-predicted concentration-time profiles in human plasma (**a** and **b**) and blood (**c** and **d**) following chloroquine (CQ) or hydroxychloroquine (HCQ, **e** and **f**) administration. The experimental data (dots) were obtained from Gustafsson et al. [46], Walker et al. [47], Frisk-Holmberg et al. [77], Tett 1988 et al. [49], and Tett 1989 et al. [50]

The inhalation PBPK model (Figure 6a) includes a mechanistic model to describe the transport kinetics across the airway epithelium (Figure 6b) and predict the pharmacokinetics. The PBPK model was adapted to species-specific physiology including airway pH [7, 29, 31, 43, 44], and model qualification was performed using rodent and human PK data obtained from literature [7, 45–47]. Upon intraperitoneal (i.p) administration of 10 mg/kg CQ to rats, the plasma *C*_max_ was 0.14 μg/mL, and the terminal elimination half-life was 75.4 h, while the lung tissue exposures were higher with a *C*_max_ of 18.1 μg/mL and a half-life of 143 h due to lysosomal trapping (Figure 7a). In mice, i.p. administration of 20 mg/kg of HCQ had a terminal elimination blood half-life of 25.6 h and a *C*_max_ of 3.09 μg/mL, while the lung tissue half-life was 31.5 h, and *C*_max_ was 20.5 μg/mL (Figure 7b–d). The differences in lung tissue elimination half-life between the two drugs are significant not only because of their physicochemical properties but also because of the physiological differences in the lungs across species. The human PBPK model was adapted to human physiology by setting the airway surface fluid or mucus and intracellular and interstitial pH to 6.6, 6.8, and 7.34, respectively [43, 44]. Because the human intralysosomal pH for lung tissue was not available, a pH value of 4.5 was used, on the basis of measurements performed in baboons [48]. The PBPK models were simulated to fit plasma CQ and blood HCQ concentrations to intravenous and oral dosing data obtained from Gustafsson et al. [46], Tett 1988 et al. [49], and Tett 1989 et al. [50] (Figure 8 a, b, e, and f). Further CQ PBPK model qualification was performed by simulating long-term PK (blood concentrations) measured by Frisk-Holmberg et al [51] (Figure 8 c and d). The parameters for liver clearance and oral absorption were optimized (Table V). A large coefficient of variation (%CV) was obtained for few model parameters due to possible variations in early experimental pharmacokinetic data points obtained while graphing figures from literature. The terminal elimination half-life for CQ in plasma is 787 h and that for HCQ in blood is 983 h.

### Inhalation of CQ and HCQ Aerosols Achieves Efficacious Lung Concentrations

**Figure 9:**
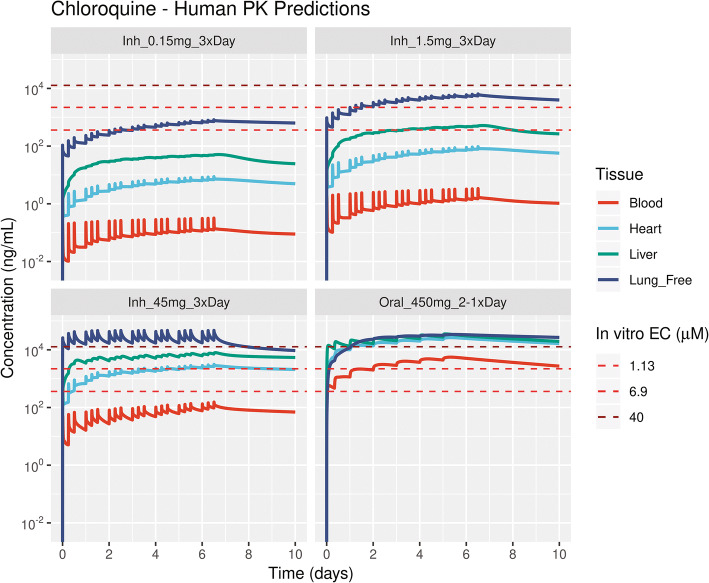
Physiologically based pharmacokinetic model-predicted inhalation and oral dosing regimens for chloroquine. The dashed lines represent the *in vitro* to effective concentration 1.13 μM (362 ng/mL), 6.9 μM (2.2E3 ng/mL) values from Wang et al. [1], and 40 μM (12.8E3 ng/mL). Dosing regimens: “Inh_0.30mg_3×Day” is inhaling 0.3 mg t.i.d.; “Inh_1.5mg_3×Day” is inhaling 1.5 mg t.i.d.; “Inh_45mg_3xDay” is inhaling 45 mg t.i.d.; “Oral_450 mg_2-1×Day” is oral administration of 450 mg b.i.d. on day 1 followed by 450 mg q.d. from day 2 to day 5 was tested in clinic [12]. Lung_Free, ﻿total unbound lung concentration

**Figure 10:**
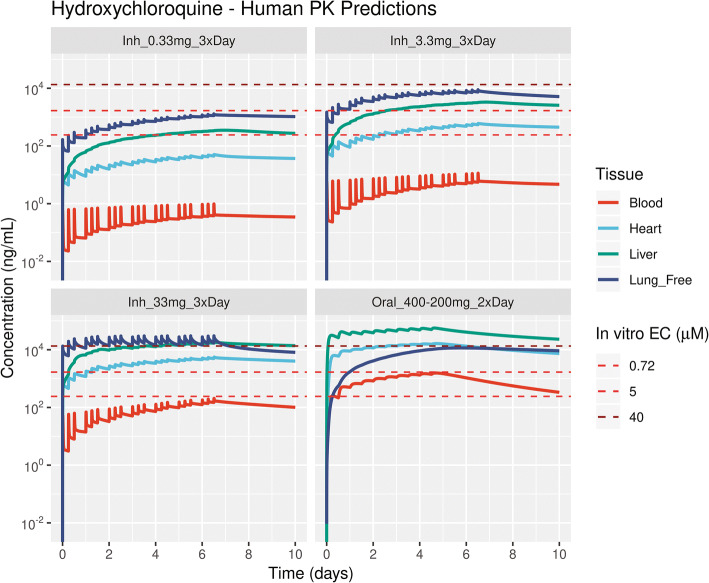
Physiologically based pharmacokinetic model-predicted inhalation and oral dosing regimens for hydroxychloroquine. The dashed lines represent the *in vitro* to effective concentration 0.72 μM (242 ng/mL), 5 μM (1.68E3 ng/mL) from Yao et al. [3], and 40 μM (13.4E3 ng/mL) from de Reus et al.[54]. Dosing regimens: “Inh_0.33mg_3×Day” is inhaling 0.33 mg t.i.d.; “Inh_3.3mg_3×Day” is inhaling 3.3 mg t.i.d.; “Inh_33 mg_3×Day” is inhaling 33 mg t.i.d.; “Oral_400–200 mg_2×Day” is oral administration of 400 mg b.i.d. on day 1 followed by 200 mg b.i.d. from day 2 to day 5 [ClinicalTrials.gov identifier: NCT04318444]. Lung_Free, ﻿total unbound lung concentration

The human PBPK model was used to simulate the concentration time profiles of oral dosing regimens for CQ and HCQ used for treating COVID-19. The model-predicted PK profiles for clinically administered oral dosing of CQ with a dosing regimen of 450 mg b.i.d. on day 1 and 450 mg q.d. from day 2 to day 5 [12] and hydroxychloroquine sulfate with an oral dosing regimen of 400 mg b.i.d. on day 1 and 200 mg b.i.d. on days 2–5 [ClinicalTrials.gov identifier: NCT04318444] are shown in in Figure 9 and Figure 10, respectively. Oral dosing regimens of CQ were recommended to maintain the plasma concentrations below 800 ng/mL(Figure S3). The total lung unbound concentrations for oral dosing were above the *in vitro* effective concentrations reported by Wang et al. [1] and Yao et al. [3]. However, these oral dosing regimens increased the accumulation of drug in tissues such as heart, liver, and kidney, thereby limiting the delivery of higher doses or prolonged use to further increase lung concentrations (Figures S3 and S4). To overcome this, we simulated the pharmacokinetics of inhaled CQ and HCQ in humans. For an inhaled aerosol, the aerosol deposition calculations using multiple-path particle dosimetry (MPPD) model were performed for an oral inhalation scenario by selecting human Weibel lung model, upper respiratory tract volume of 55 mL, and upright body orientation [52]. The MPPD model predicted a 28.9% deposition and a 71.1% exhaled fraction per puff based on the measured aerosol physicochemical properties. The regional deposition fractions per puff were 1.17% in the upper airways, 3.05% in the conducting airways, 5.08% in the transitional airways, and 19.6% in the pulmonary airways [52]. A puffing pattern of a 3-s inhalation-exhalation with a 30-s inter-puff interval was used in the simulation. Multiple inhalation dosing regimens with an inhaled dose of 0.15 mg per puff CQ and 0.33 mg per puff HCQ with multiple puffs per session per day were simulated to predict the inhalation pharmacokinetics. Since a wide range of *in vitro* effective concentrations have been reported in Vero cells, Calu-3, and primary bronchial epithelial cells at different multiplicity of infection [3, 53, 54], we selected multiple inhalation regimens to obtain the unbound lung trough concentrations above the different *in vitro* effective concentrations (Table SIII). Inhaling a daily low emitted dose comprising 0.15 mg CQ (Inh_0.15mg_3×Day) or 0.33 mg HCQ (Inh_0.33mg_3×Day) three times a day could enable the total unbound lung concentrations to reach lower bound *in vitro* EC_50_ values (Figure 9 and Figure 10). Alternately, the unbound lung concentrations can reach *in vitro* effective concentrations of 6.9 and 5 μM with 1.5 mg CQ (Inh_1.5mg_3×Day) or 3.3 mg HCQ (Inh_3.3mg_3×Day) of emitted dose taken three times a day, respectively (Figure 9 and Figure 10). Inhaled CQ and HCQ accumulated in the airway lysosomal compartments and led to a rise in overall lung concentrations (Figures S5 and S6). The systemic concentrations of CQ and HCQ including blood, liver, kidney, and heart were significantly lower for pulmonary delivery than for oral administration (Figures 9, 10, S3, and S4). Given the lower systemic concentrations and widened therapeutic index, higher doses of CQ and HCQ could be inhaled to achieve increased effective concentrations in the lung. An emitted dose greater than 45 mg of CQ or 33 mg of HCQ inhaled three times a day may obtain unbound lung trough concentrations greater than 40 μM. Because the pharmacokinetic driver for the efficacy of CQ and HCQ in the lung tissue to treat COVID-19 is not clear, we included the concentration vs. time profiles of drug in lung compartments such as pulmonary alveolar surfactant, cytosol, lysosomes, and interstitial fluid in Figures S5 and S6. A higher amount of inhaled aerosol could be delivered to obtain desired concentrations in lung compartment of interest.

During early stages of SARS-CoV-2, increased drug deposition in upper airway could be beneficial as higher viral loads were reported in upper airway region [55]. Pulmonary delivery of inhaled aerosols could be optimized by varying aerosol particle sizes to increase regional deposition of an inhaled aerosol [56, 57]. Hence, we performed a simulation to predict PK for inhaled monodisperse and polydisperse aerosols (Table SIV, Figures S7 and S8). An increase in mass median aerodynamic diameter (MMAD) had led to a rise in systemic concentrations due to higher deposition.

## Discussion

Several inhalation-based therapies have been developed to treat a wide range of respiratory diseases. However, the study of therapeutic drugs to treat COVID-19 is focused on oral administration aiming to achieve the desired pulmonary concentrations. In this study, we found that CQ and HCQ aerosols generated by thermal aerosolization had inhalable particle sizes and did not affect functional activity in human bronchial epithelial cell cultures. A quantitative translation of *in vitro* and *in vivo* exposures using a mechanistic model enabled the prediction of human PK. Our results indicate that inhaling aerosolized CQ and HCQ could yield total lung concentrations reaching *in vitro* effective concentrations while minimizing systemic exposures.

Thermal aerosolization involves generation of aerosol particles by evaporating the liquid formulation and subsequently cooling it to nucleate and condense from super-saturated vapors. Such an approach under controlled thermal conditions allows the generation of micrometer and even sub-micrometer aerosol particles that are easily inhalable and reach the deep lungs [58]. The thermal aerosolization device maintains a heating temperature in the range of 200–220°C, which is suitable for evaporation of aerosol formers such as propylene glycol. In addition, the use of the metal mesh heater to evaporate the liquid prevents potential decomposition and release of unwanted chemicals from the device. During our investigation, we observed no distinct retention time of extracted samples for 40 mg/mL CQ or 100 mg/mL HCQ, indicating no decomposition (i.e., no water loss; Figures S9 and S10). In contrast, non-thermal liquid aerosolization (e.g., nebulization) is free from the potential products of thermal decomposition but typically results in larger particle sizes at moderate pressure levels, impacting the delivery to the deep lungs. The PK of such inhaled aerosols delivered from multiple nebulizers with different particle size ranges and inhalation maneuvers can be simulated using the inhalation PBPK model to obtain a greater understanding for respiratory drug delivery (Table SIV, Figures S7 and S8).

CQ and HCQ are known to accumulate in the acidic regions and undergo ion-trapping [6]. The physiological pH of the airway surface fluid and cells lining the respiratory tract was measured as acidic, favoring accumulation of CQ and HCQ [37]. However, these pH values differ across species and rodents have lower acidic levels than humans [42, 43]. These differences will lead to species-dependent differential pulmonary exposures, and a direct extrapolation from rodents to humans may result in differing exposure predictions. The pharmacokinetics and ion-trapping of aerosolized CQ and HCQ could be influenced by pathophysiological changes during a disease. During respiratory illness, the airway surface liquid is more acidic (pH around 5) due to a decrease in buffering capacity [44], which may further increase the retention of CQ and HCQ [38]. This increased retention from pulmonary delivery can be more effective than oral dosing, as the postulated mechanism of viral uptake from the respiratory tract can be obstructed by altering the pH of the airway surface liquid and lysosomes, thereby inhibiting the endosomal uptake mechanism and intracellular lysosomal release for viral replication. An increased concentration of CQ or HCQ in airway surface liquid can also interfere with the glycosylation of ACE2 and thus reduce its binding efficiency with host cells and the spike protein on the surface of the coronavirus [4]. Depending upon the activity of ion transporters to maintain ion homeostasis [44] and pH differences in respiratory tract across population, pulmonary delivery of very high doses of chloroquine or hydroxychloroquine could also facilitate rapid transfer across the airway by lowering acidic pH and remains to be evaluated. Recent publications predicted pulmonary concentrations using the lung-to-plasma partition coefficient obtained from rat data [3, 59], but the lysosomal ion-trapping mechanisms of these drugs generate a non-linear equilibrium across airway epithelia and plasma as the acidic pH increases [7, 49, 60, 61]. Ruiz et al. have measured HCQ concentrations in epithelial lining fluid for orally administered HCQ indicating an apical transport [62]. Based on Weiss et al. and our *in vitro* modeling outcomes, we hypothesize transport of HCQ to apical surface via P-gp efflux transporter [40], and since a similar transporter expressed on lysosomes, further studies need to be performed for evaluating the percent contribution of P-gp-driven CQ or HCQ lysosomal influx. Also, the cell type specific lysosomal volumes could lead to differential intracellular distribution of drugs and needs to be studied [7]. In our study, the HBEC and IPML models were qualified without the lysosomal P-gp transporter and were further not included in the inhalation PBPK model. Since transporter activity could influence lung exposures, experimental studies need to also account for any diseased induced changes in P-gp functional activity and pH changes. Although Collins et al. developed the PBPK model in context of cancer-autophagy and Liu et al. developed PBPK model by including lysosomal kinetics, the pulmonary concentrations for COVID-19 treatment are more likely to be over predicted due to minimal airway model description [7, 60]. Our mechanistic inhalation PBPK model accounts for airway regional differences, P-gp active transporter, and lysosomal uptake kinetics along with species-specific physiology to predict the tissue distribution of CQ and HCQ, rendering model predictions more physiologically relevant. The limitations for the current PBPK model include assuming the lysosomal volume and pH to be same for all the cells in each tissue. Further model development with cell-type specific lysosomal volumes [63] and endo-lysosomal pH (between 4.5 and 6.8) [64] could deliver insights into intra-tissue distribution of drugs.

Clinical efficacy of orally administered CQ and HCQ still remains inconclusive as unbound lung interstitial concentrations (Figures S5 and S6) were below the *in vitro* extracellular EC_50_ values [65]. Few clinical studies have reported improved patient outcomes for orally administered HCQ as lower bounds of effective concentrations were probably achieved [15, 66]. *In vitro* efficacy of CQ and HCQ was initially evaluated in Vero cells, and multiple studies reported EC_50_’s ranges between 0.72 and 22.3 μM based on the multiplicity of infection [1–3, 65]. Hoffman et al. reported EC_50_’s of 100 μM for CQ and HCQ in Calu-3, an immortalized human airway epithelial cell line infected with SARS-CoV-2 virus [53]. The difference in EC_50_’s may be related to the mechanisms behind host entry into the cell. Based on the expression of receptors and cell type, coronavirus may enter via the endocytic pathway in Vero cells or via plasma membrane fusion in Calu-3 cells [67–69]. While cell lines could serve as a cellular model to understand the fractional contribution of each mechanism for obtaining efficacy and related effective concentrations, evaluating CQ and HCQ *in vitro* efficacy in primary human respiratory epithelium cultures may be more clinically relevant. An exposure of 10 μM HCQ to SARS-CoV-2 infected primary human 3D alveolar organoid cultures resulted in a 2.4-log reduction in viral N gene expression [70]. A recent study by de Reus et al. reported a significant reduction in SARS-CoV-2 viral load in infected primary human epithelial cells exposed to 40 μM HCQ [54]. Preliminary *in vitro* studies have shown that the pharmacokinetics driver for efficacy could be time above EC_50_ [3] and can be achieved by inhaling low doses with a t.i.d regimen. Low inhaled doses caused the total unbound lung concentrations to reach high bound *in vitro* effective concentrations (Figures 9 and 10).

The 3D organotypic human bronchial airway cultures maintained at the ALI represents one of the most physiological *in vitro* lung models [71]. Ciliary beating, mucus secretion, and the formation of a tight epithelium are some of the key functionalities in the human lungs reproduced by this *in vitro* organotypic culture system. CQ and HCQ did not impair the functioning of ciliated cells suggesting no changes in viscosity of the airway surface fluid, the number of cilia, and their length—three factors known to affect ciliary beating frequency [72]. For deposited doses over 7.24 μg CQ and 7.99 μg HCQ, a concentration-dependent decrease in the active ciliary beating area was observed in tissues exposed to CQ and to a lesser extent in those exposed to HCQ. While an increase or decrease in mucociliary transport has been demonstrated for other drugs [73], the mechanism leading to a reduction in active ciliary beating without affecting the ciliary beating frequency remains to be determined. Finally, the measurements of TEER and ATP following exposure showed that tissue integrity and viability were not affected by the treatment, as the values recorded were similar for drug- and air-exposed tissues. No cytotoxicity was observed for a deposited dose of 7.24 μg CQ and 7.99 μg HCQ on human bronchial tissue with a surface area of 0.33 cm^2^. An extrapolation to determine tolerable human deposited dose for pulmonary toxicity based on the surface area of the human lung (6.1 × 10^5^ cm^2^) was calculated as 14.6 g for a 24-h exposure [74]. A clinical trial involving inhaled HCQ sulfate of doses up to 50 mg/day for 7 days showed that these doses were safe and in agreement with our *in vitro* findings [75].

Unlike oral dosing, pulmonary drug delivery is complex, especially with the variation in inhalation maneuvers that can lead to varying doses. Estimating the deposited dose in the respiratory tract for an inhaled evolving aerosol is challenging [57]. As an example, the multiple-path particle dosimetry model-predicted deposited fractions are based on the assumption that the aerosol is non-evolving [52]. Given the variability and lack of human data, future clinical studies should measure airway surface liquid and whole blood concentrations in human participants. Because low doses of inhaled CQ and HCQ may deliver the desired pulmonary concentrations with minimum systemic exposure, there is scope for prophylactic use against COVID-19 in healthy subjects in communities with high infection rates. Further, pulmonary delivery of CQ and HCQ aerosols enables dose titration for an individualized treatment, and hence early-stage infected subjects can receive a higher dose. However, the effective concentrations for CQ and HCQ remains unclear as it varies with multiplicity of infection and cell type [1, 3, 53]. Hence, clinical studies need to be performed to evaluate the efficacy of aerosolized CQ and HCQ in the treatment of COVID-19. The development and clinical evaluation of inhaled therapeutics becomes more important as the SARS-CoV-2 is mutating into more contagious variants.

## Conclusions

We formulated CQ and HCQ aerosols with respirable particles for use in the treatment of COVID-19. An *in vitro* assessment in human bronchial epithelial cells showed no adverse effects on cell viability, TEER, or ciliary beating. Modeling CQ and HCQ exposures *in vitro* and in an IPML model enabled the validation of transport kinetics across the airway epithelial barrier. The PBPK model predicted that inhaling an emitted dose of 1.5 mg CQ or 3.3 mg HCQ three times a day may yield in vitro reported efficacious concentrations in the lung while minimizing systemic exposure. We hope that inhalation-based delivery of drugs using an optimized dosing schedule would accelerate the treatment of COVID-19-infected patients.

## Supplementary information

ESM 1 (PDF 2.01 mb)

## Glossary

BP: blood to plasma ratio
CA: conductional airway
CQ: chloroquine
DC: diffusion coefficient
*f*_u_: unbound protein fraction
*f*_ub_: unbound fraction in blood
GFR: glomerular filtration rate
GI: gastrointestinal tract
HBEC: human bronchial epithelial cultures
HCQ: hydroxychloroquine
IPML: isolated perfused mice lung
Km: Michaelis–Menten half-maximal rate constant
*logKow*: logarithmic octanol-water partition coefficient
PA: pulmonary alveolar region
PBPK: physiologically based pharmacokinetic
P-gp: P-glycoprotein
SA: surface area
TA: transitional airway
*T*: thickness
UA: upper airway
Vmax: Michaelis–Menten maximal reaction rate
