# Supplementary material for: Pulmonary Delivery of Aerosolized Chloroquine and Hydroxychloroquine to Treat COVID-19: In Vitro Experimentation to Human Dosing Predictions
Source: AAPS J. 2022 Feb 7;24(1):33. doi: 10.1208/s12248-021-00666-x (PMC8821864; doi:10.1208/s12248-021-00666-x)
Supplement: Supplementary file 1 — (PDF 2.01 mb) [file 12248_2021_666_MOESM1_ESM.pdf]

Supplementary Information

Pulmonary Delivery of Aerosolized Chloroquine and Hydroxychloroquine to Treat COVID-19: In Vitro Experimentation to Human Dosing Predictions

Authors:

Aditya R. Kolli, Tanja Zivkovic Semren, David Bovard, Shoaib Majeed, Marco van der Toorn, Sophie Scheuner, Philippe Guy, Arkadiusz Kuczaj, Anatoly Mazurov, Stefan Frentzel, Florian Calvino-Martin, Nikolai Ivanov, John O'Mullane, Manuel C. Peitsch, Julia Hoeng*

Affiliation:

PMI R&D, Philip Morris Products S.A., Quai Jeanrenaud 5, CH-2000 Neuchâtel, Switzerland

***Corresponding author:**

Julia Hoeng

PMI R&D, Philip Morris Products S.A.

Quai Jeanrenaud 5, CH-2000 Neuchâtel, Switzerland

Phone: +41 (58) 242 2892

Email: Julia.Hoeng@pmi.com

TABLE OF CONTENTS

SUPPLEMENTARY METHODS **3**

SUPPLEMENTARY TABLES **4**

SUPPLEMENTARY FIGURES **7**

SUPPELEMENTARY MODEL CODE **18**

SUPPLEMENTARY METHODS

Model-driven evaluation for HCQ transport across HBEC

To develop and qualify in vitro model for HCQ kinetics across a 3D organotypic HBEC, we performed model-driven evaluation to determine the model structure. Ruiz et al. have measured the HCQ concentrations in human epithelial lining fluid (ELF) for orally administered HCQ [62], which clearly indicates the transport of HCQ across the airway epithelium. To date no experimental measurements were performed to evaluate transporter activity of HCQ across airway epithelium. We first developed a basic model framework using experimental parameters such as tissue thickness, volumes, surface area of inert containing organotypic HBEC etc.

The transport kinetics of HCQ in HBEC were simulated using apparent permeability measured in Caco2 cells (Figure S11A). The apparent permeability (Papp = 0.13e-12 cm/s) of Caco2 was too low leading to minimal transport across HBEC [61]. Using a higher apparent permeability (Papp = 0.13e-7 cm/s), the model was able to predict initial basal concentrations but lead to an underprediction of apical concentrations (Figure S11B). Because the transport kinetics of apical→basal and basal→apical transport could be different, we simulated the model by assuming different permeabilities from the apical to basal (Papp = 0.13e-7 cm/s) and basal to apical (Papp = 0.13e-6 cm/s) compartments, as shown in Figure S11C. A higher basal → apical permeability was able to predict the apical concentrations but not the initial basal concentrations. Further optimizations could not predict HCQ in vitro kinetics in HBEC. We then simulated the model using the model framework from Trapp et al. and without the P–gp transporter (Figure S11D). The transport of HCQ across the HBEC was rapid, and less than 0.01% of HCQ remained in the apical layer. As CQ was transported by P–gp in the IPML ex vivo model [26] and Weiss et al. have shown HCQ to be a substrate for the P–gp transporter [40], we incorporated the P–gp transporter and simulated the model to predict HCQ kinetics (Figure 4).

SUPPLEMENTARY TABLES

Table SI: Solubility of chloroquine and hydroxychloroquine in propylene glycol evaluated at different concentrations. Measurements were performed by LC-HR-MS.

Drug	Concentration (mg/mL)	Accuracy (%)
Chloroquine	1.0	96
	5.4	84
	10.1	94
	20.2	80
	40.7	81
Hydroxychloroquine	1.3	78
	10.2	103
	20.9	104
	40.6	92
	100	96

Table SII: Aerosol deposition in cell-free Vitrocell inserts.

Number of puffs	Chloroquine		Hydroxychloroquine	
	Mean (µg) ± SD	Deposition (%)	Mean (µg) ± SD	Deposition (%)
25	7.24 ± 0.84	0.23	7.99 ± NA	0.27
50	13.2 ± 1.4	0.21	15.9 ± NA	0.27
100	12.9 ± 1.6	0.10	28.3 ± 1.8	0.24

SD, standard deviation.

Table SIII: PBPK model-predicted trough concentrations of CQ and HCQ in blood, heart and liver, and unbound trough concentrations of CQ and HCQ in lung interstitial space and total lungs. CQ in vitro EC_{50} and EC_{90} values are 362 ng/mL and 2.2E3 ng/mL [1]. HCQ in vitro EC_{50} and EC_{90} values are 242 ng/mL and 1.68E3 ng/mL [3]. Lung_Intersitital_Free, total unbound concentrations in lung interstitial space; Lung_Free, total unbound concentrations in the lungs.

**Drug**	**Route**	**Trough Concentration (ng/mL)**			
		**Blood**	**Heart**	**Lung_Free**	**Lung_Interstitial_Free**
CQ	Inh_0.15mg_3xDay	0.1	6.3	644	0.02
	Inh_1.5mg_3xDay	1.4	71.5	5E3	0.4
	Inh_45mg_3xDay	77.2	2.3E3	17.4E3	8.8
	Oral_450mg_2-1xDay	5.1E3	24.7E3	33.3E3	185
HCQ	Inh_0.33mg_3xDay	0.3	39.1	1E3	0.02
	Inh_3.3mg_3xDay	4.9	481	6.9E3	0.7
	Inh_33mg_3xDay	126	4.5E3	14.5E3	6.4
	Oral_400-200mg_2xDay	1.1E3	13.2E3	11.5E3	18.7

Table SIV: MPPD-predicted aerosol deposition for oronasal inhalation of generic monodispersed and polydispersed aerosol particles generated from nebulizers. MMAD, mass median aerodynamic diameter; GSD, geometric standard deviation; MPPD, Multiple-path particle dosimetry model; TB, Tracheobronchial; PA, Pulmonary alveolar.

**Type of aerosol**	**Aerosol particle sizes**		**MPPD-predicted aerosol deposition fractions**		
	**MMAD (μm)**	**GSD**	**Head region**	**TA region**	**PA region**
Monodispersed	1	1.5	0.10	0.07	0.14
	2	1.5	0.24	0.09	0.23
	3	1.5	0.36	0.12	0.25
	4	1.5	0.46	0.15	0.22
Polydispersed	2	1	0.22	0.09	0.25
	2	1.5	0.24	0.09	0.23
	2	2	0.26	0.10	0.20
	2	2.5	0.29	0.10	0.18

SUPPLEMENTARY FIGURES

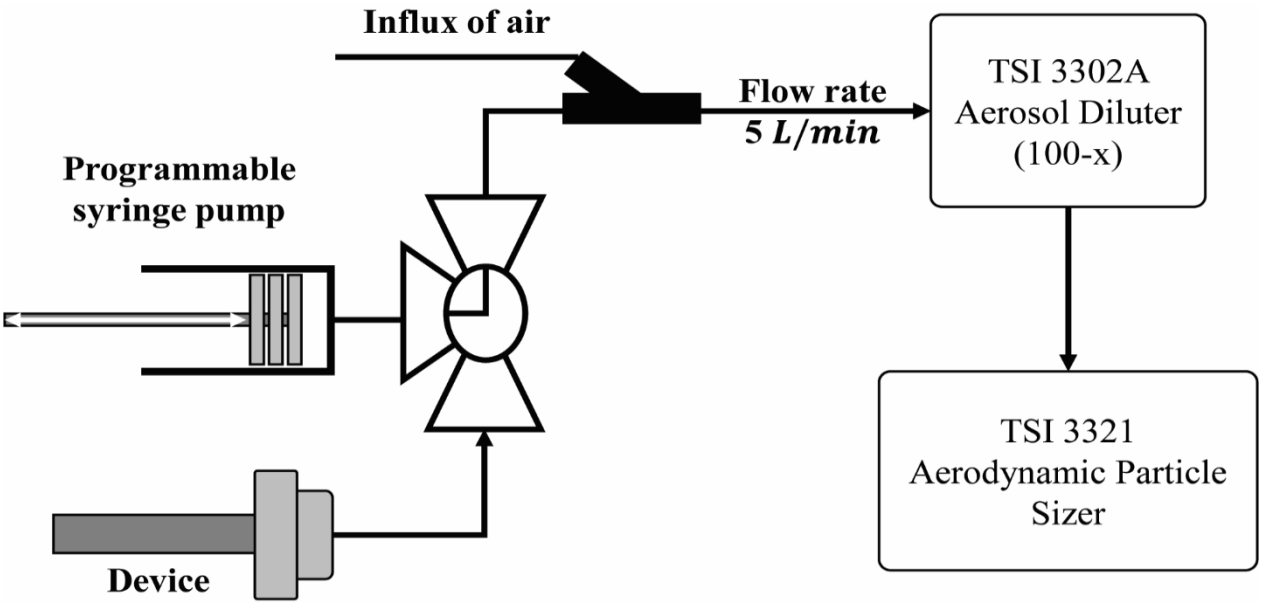

Figure S1: Schematic layout of the thermal aerosolization device connected to an aerodynamic particle sizer for measuring aerosol particle diameter.

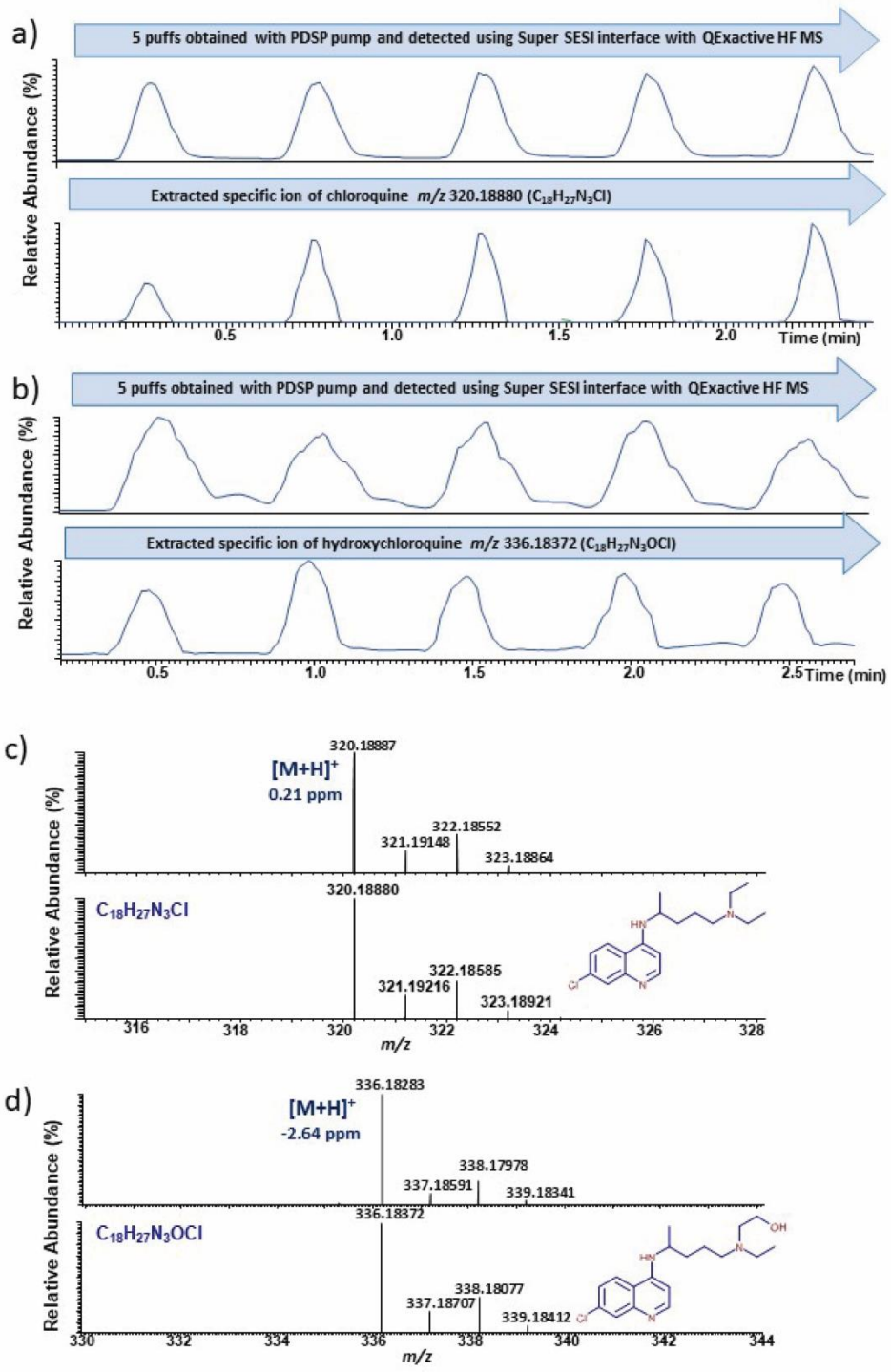

72

73 **Figure S2**: Total ion current and corresponding extracted protonated species obtained from several puffs
74 of CQ (a) and HCQ (b) aerosol. The associated full-scan positive electrospray mass spectra and theoretical
75 expected mass spectra for CQ (c) and HCQ (d).

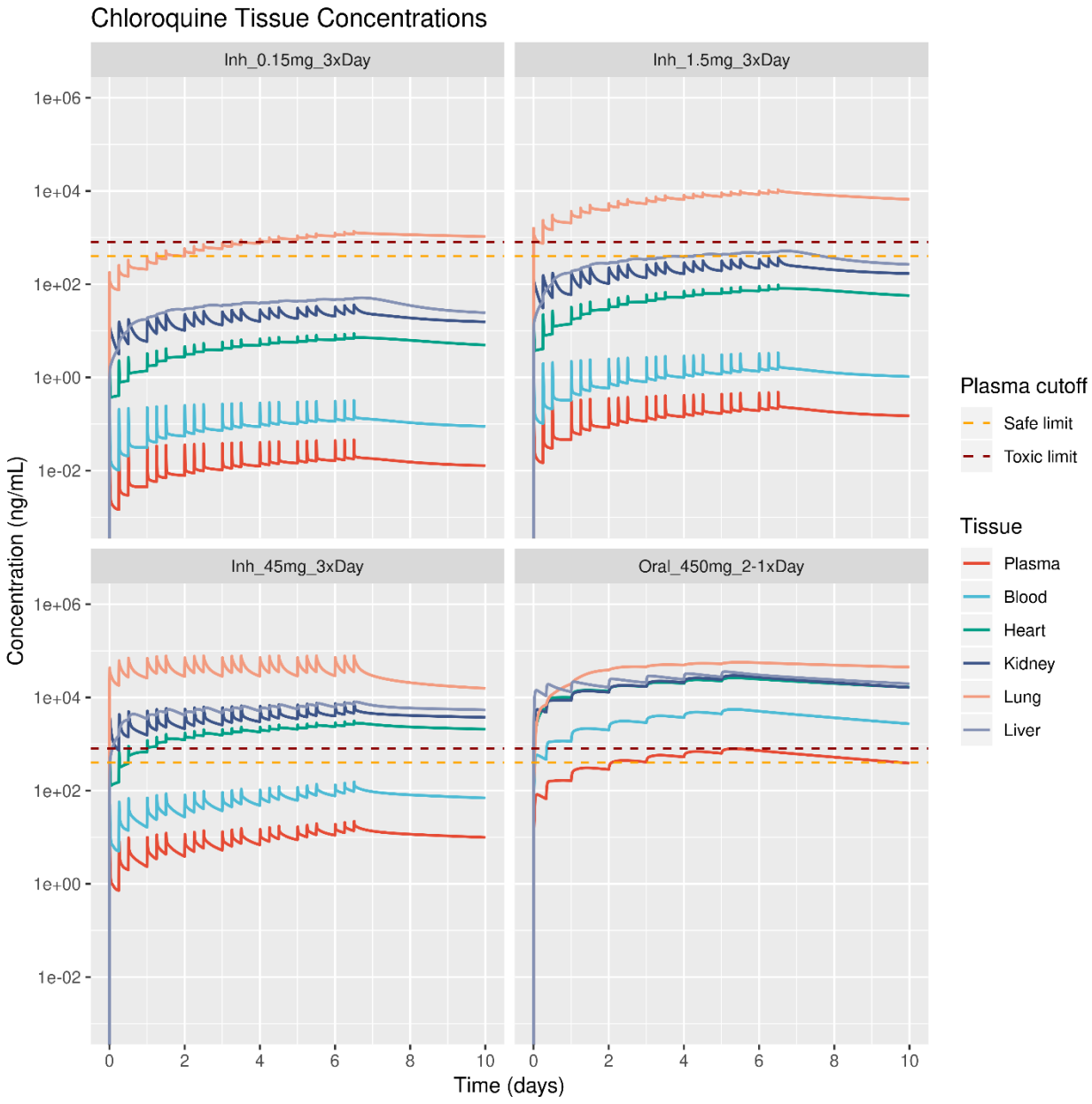

Figure S3: Simulated human chloroquine concentrations in tissues under multiple dosing regimens. The safe limit (orange dashed line) of 400 ng/mL is the plasma concentration below which no toxicity was reported [51, 59]. The toxic limit (red dashed line) of 800 ng/mL is the plasma concentration at which 80% subjects reported adverse effects [59].

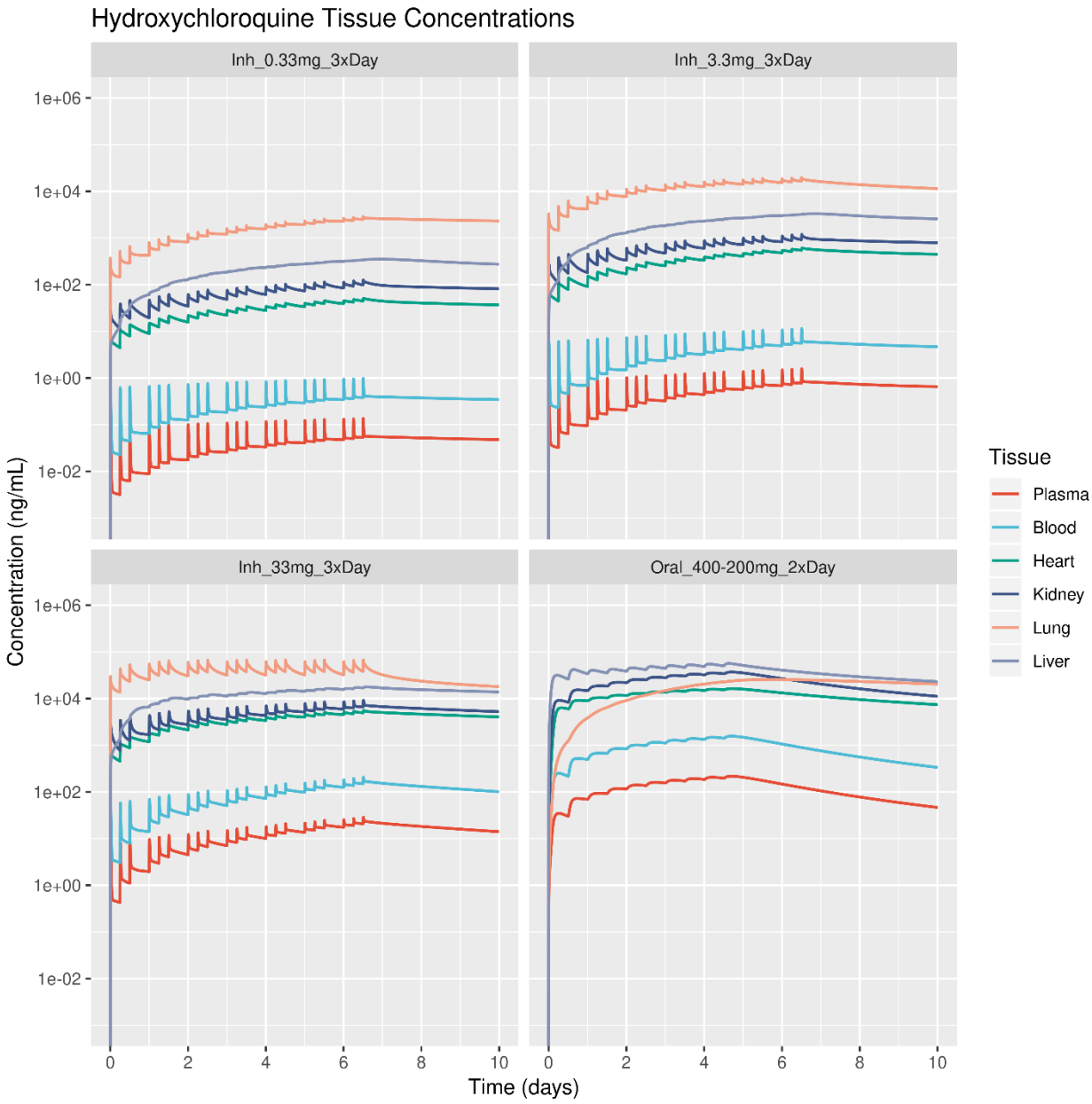

81

82 **Figure S4**: Simulated human hydroxychloroquine concentrations in tissues under multiple dosing

83 regimens.

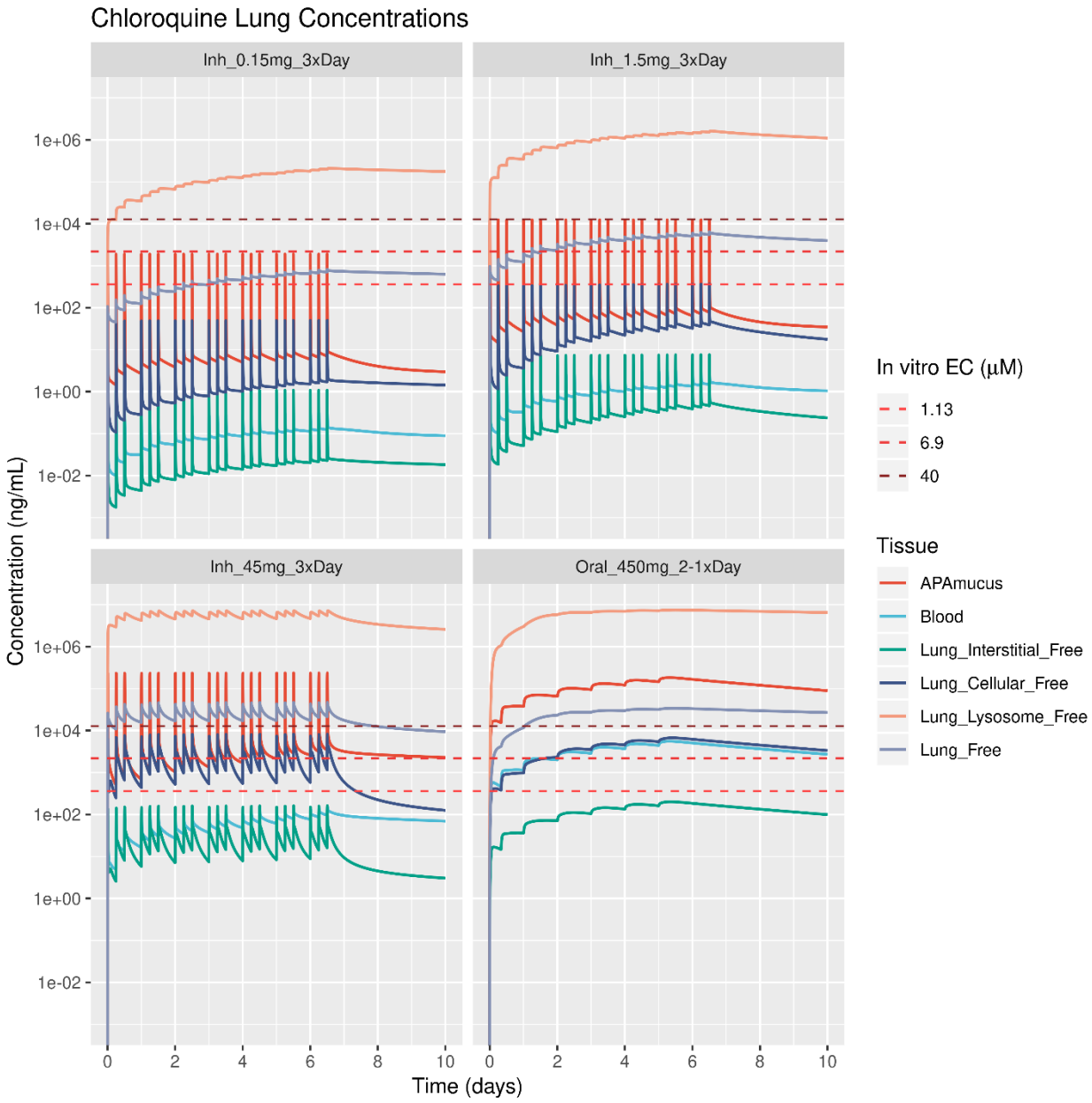

Figure S5: Simulated chloroquine concentrations in different compartments representing the human lungs. The lung compartments are shown in Figure 5B. The dashed lines represent the in vitro effective concentrations 1.13 μM (362 ng/mL) and 6.9 μM (2.2E3 ng/mL) from Wang et al.[1] and 40 μM (12.8E3 ng/mL). APAmucus, pulmonary alveolar surfactant; Lung_Cellular_Free, unbound non-lysosomal cellular concetrations; EC, effective concentration.

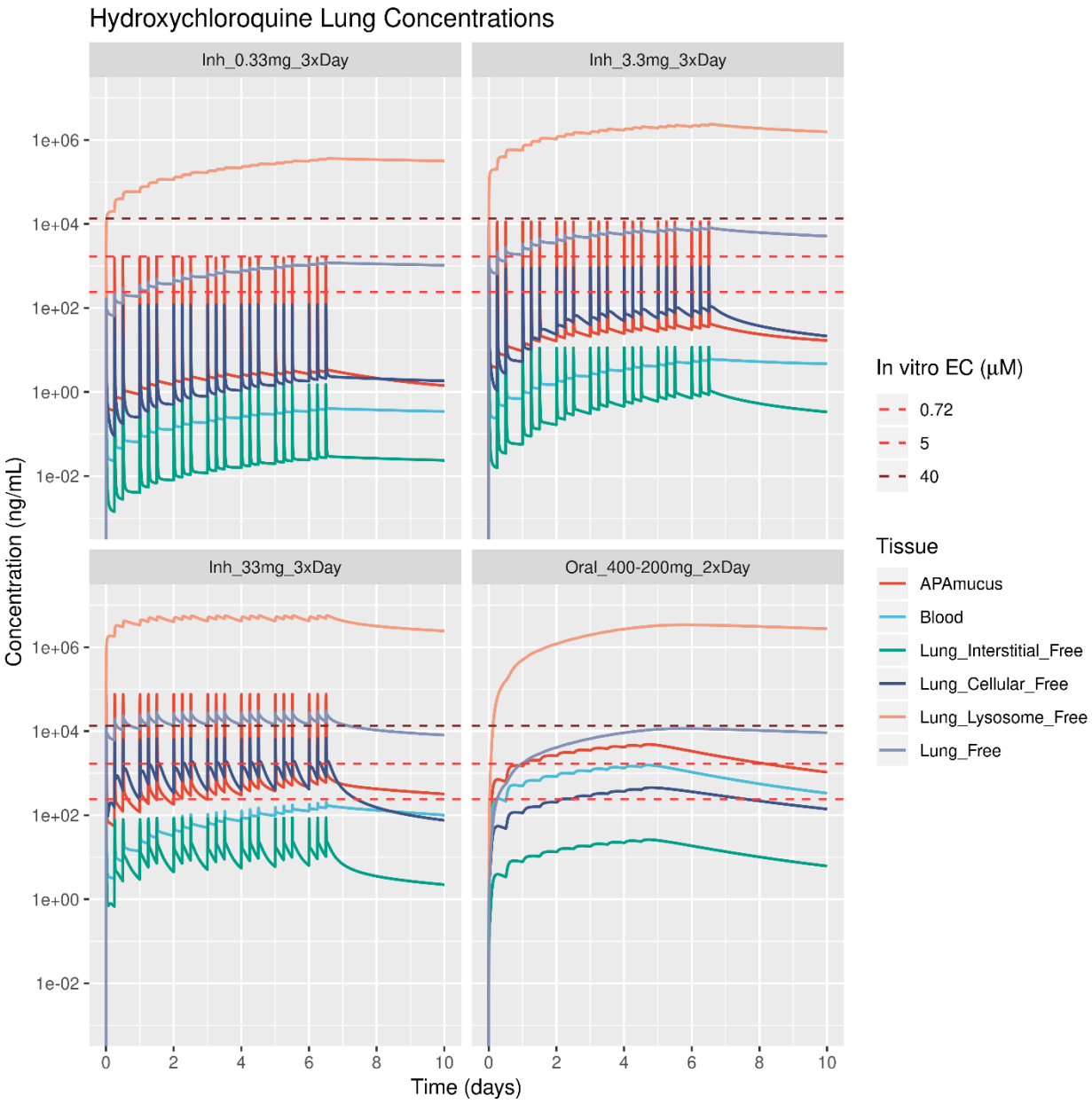

Figure S6: Simulated hydroxychloroquine concentrations in different compartments representing the human lungs. The lung compartments are shown in Figure 5B. The dashed lines represent the in vitro effective concentrations 0.72 µM (242 ng/mL) and 5 µM (1.68E3 ng/mL) from Yao et al.[3] and 40 µM (13.4E3 ng/mL) from de Reus et al.[54]. APAmucus, pulmonary alveolar surfactant; Lung_Cellular_Free, unbound non-lysosomal cellular concetrations; EC, effective concentration.

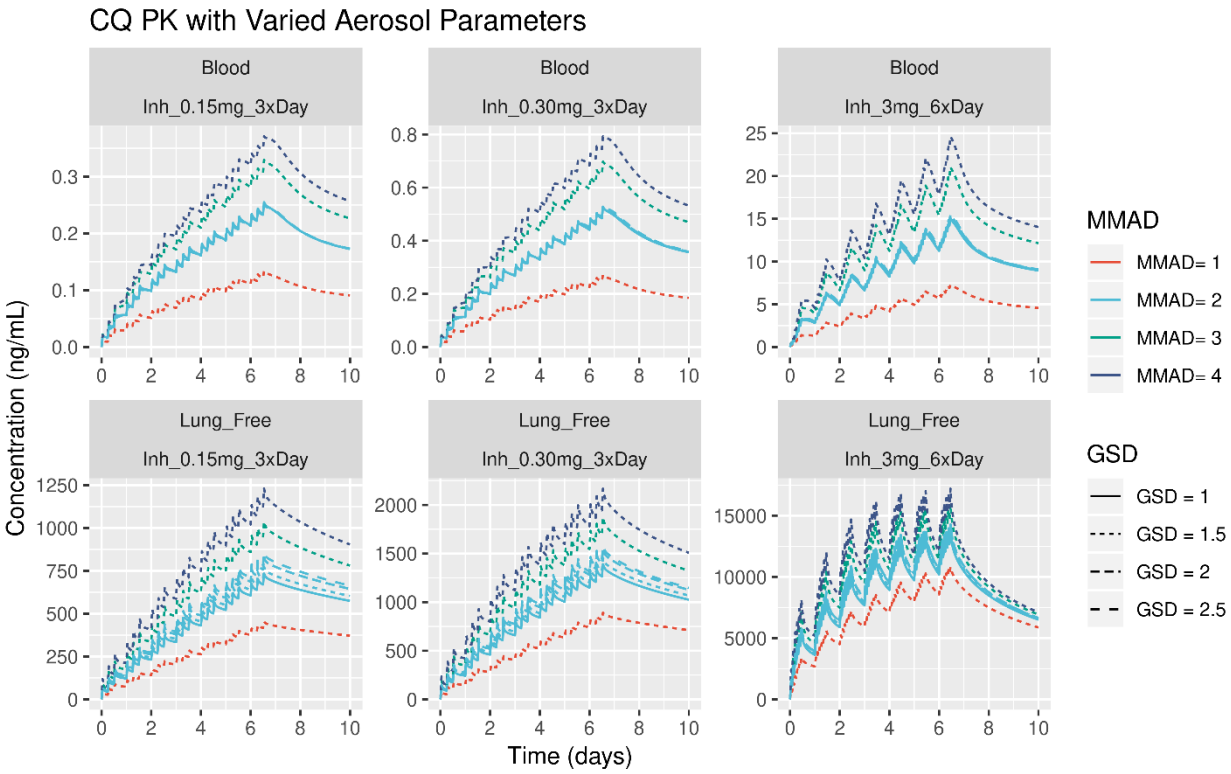

Figure S7: Model-predicted chloroquine concentrations in blood and total unbound lung concentrations (Lung_Free) of monodisperse and polydisperse aerosols with different aerosol particle sizes. MMAD, mass median aerodynamic diameter; GSD, geometric standard deviation.

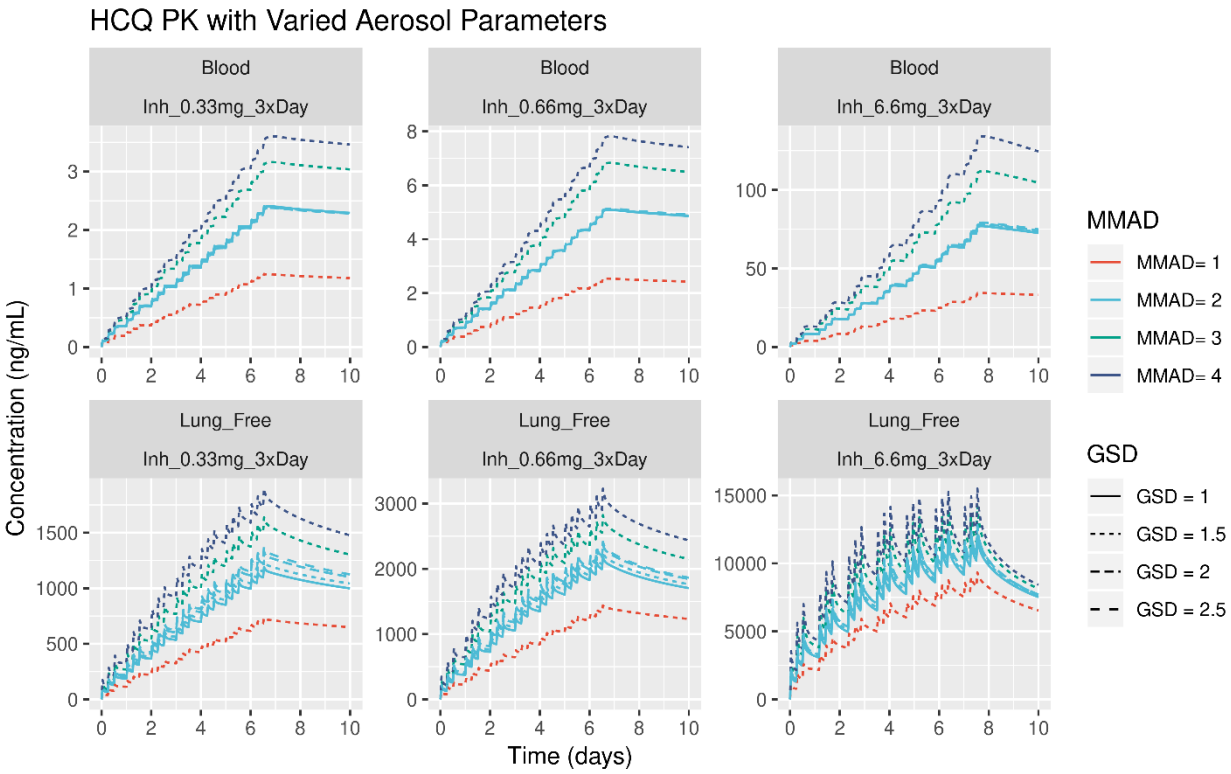

101

102 **Figure S8**: Model-predicted hydroxychloroquine concentrations in blood and total unbound lung

103 concentrations (Lung_Free) of monodisperse and polydisperse aerosols with different aerosol particle

104 sizes. MMAD, mass median aerodynamic diameter; GSD, geometric standard deviation.

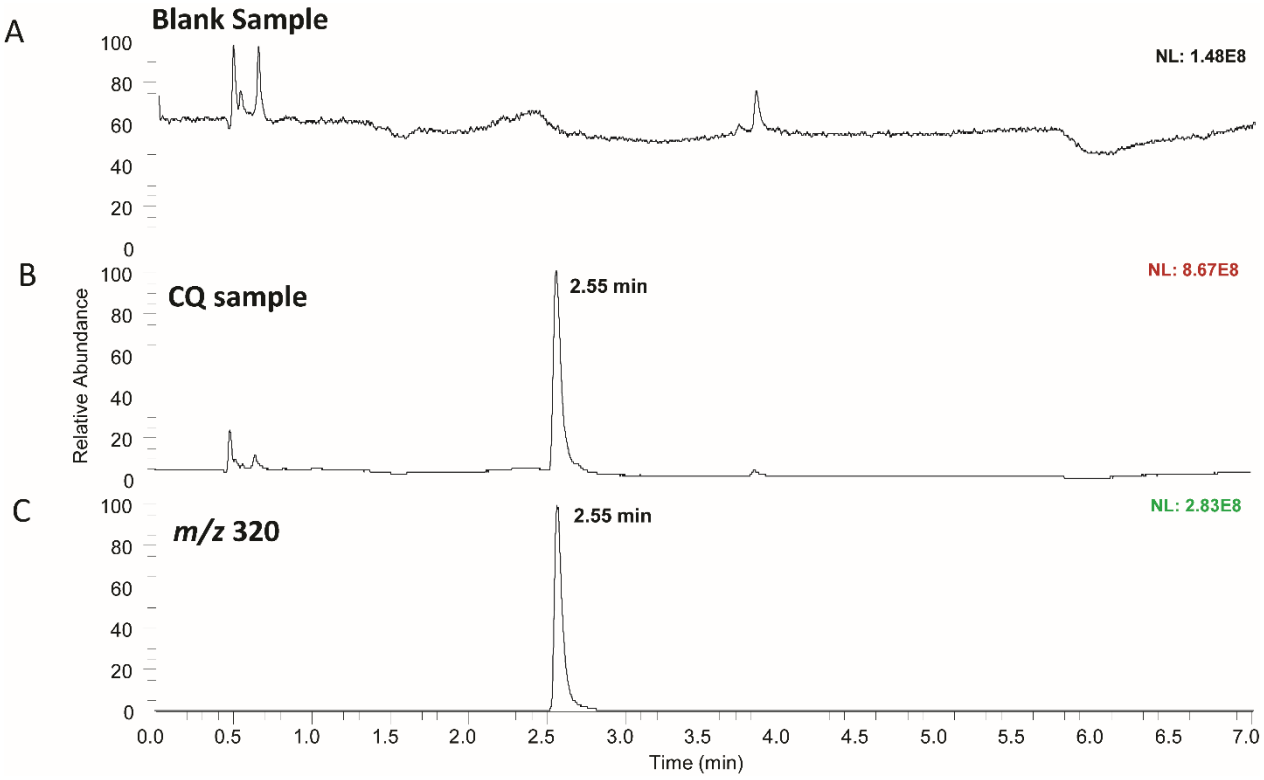

Figure S9: LC–HR-MS analyses of blank sample and an aerosol sample of CQ trapped in Cambridge filter pads. (A) Blank sample showing peaks originating from the column (background contamination); (B) total ion current of the CQ sample, showing a clear peak at retention time of 2.55 min; (C) accurate mass extraction of chloroquine (*m/z* 320) from the CQ sample (5 ppm mass tolerance). NL, noise level

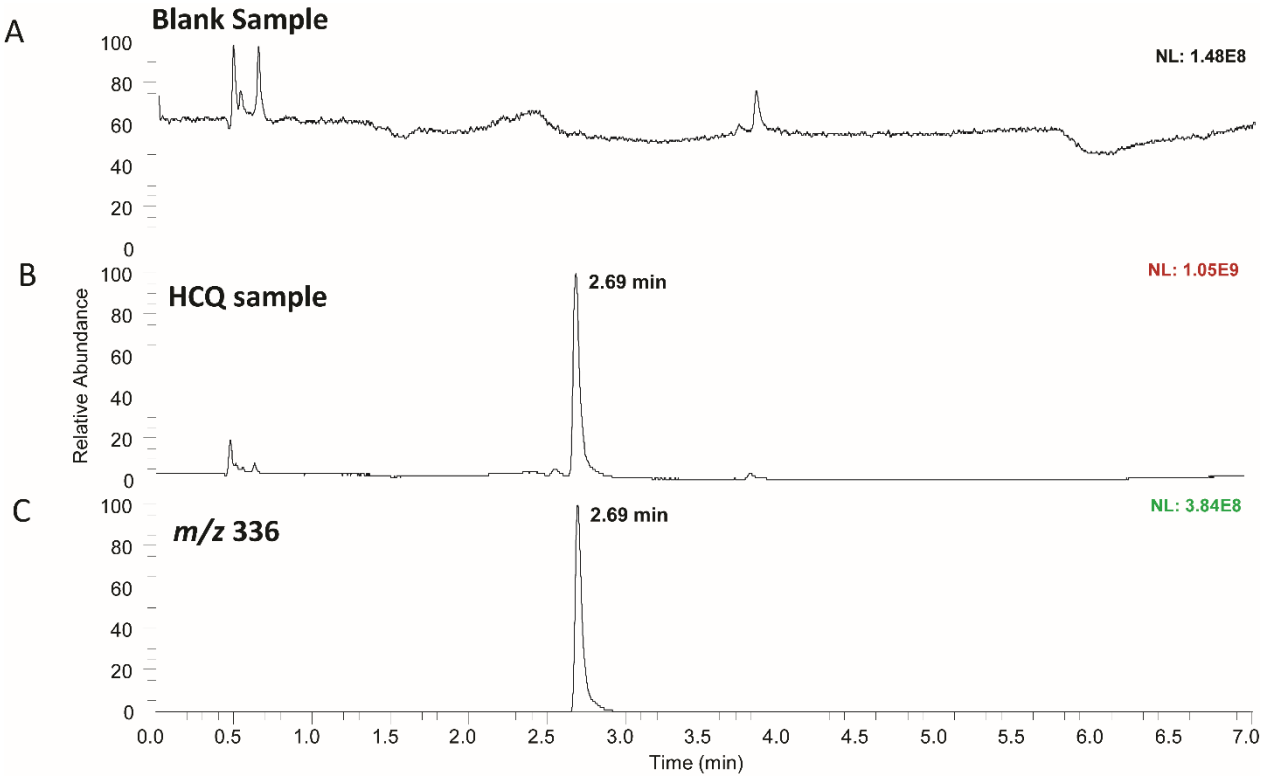

Figure S10: LC–HR-MS analyses of blank sample and an aerosol sample of HCQ trapped in Cambridge filter pads. (A) Blank sample showing peaks originating from the column (background contamination); (B) total ion current of the HCQ sample, showing a clear peak at retention time of 2.69 min; (C) accurate mass extraction of hydroxychloroquine (*m/z* 336) from the HCQ sample (5 ppm mass tolerance). NL, noise level

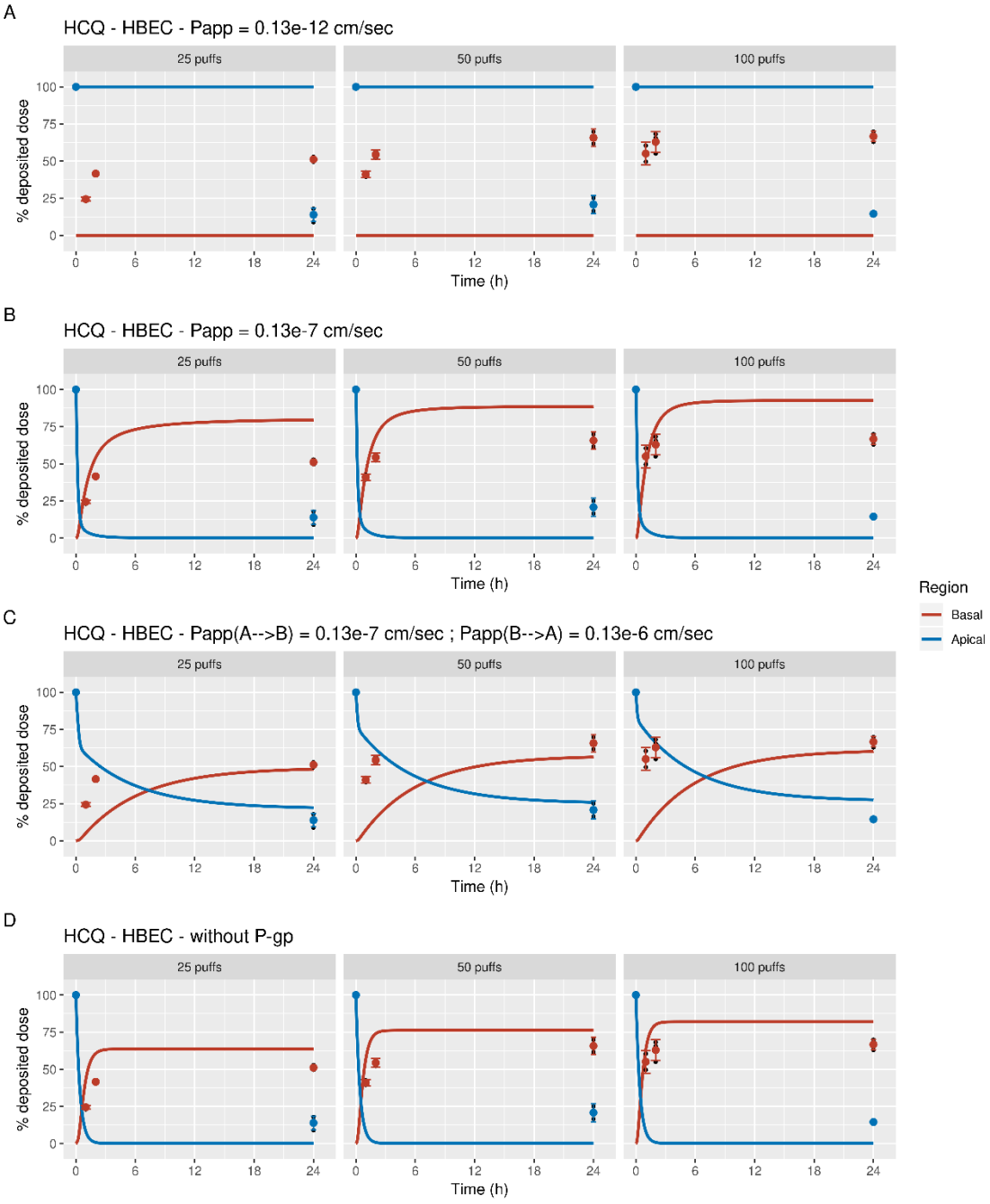

Figure S11: Model-driven evaluation of the in vitro kinetic model for HCQ in HBEC without P–gp apical efflux transporter. The deposited amount of HCQ for 25 puffs was 7.99 µg, 50 puffs was 15.9 µg, and 100 puffs was 28.3 µg. Simulated using Caco2 permeability (A), with a higher permeability (B), and with different apical and basal permeabilities (C). Diffusive flux-based model without the P–gp transporter (D).

SUPPELEMENTARY MODEL CODE

```
#=====================================================================#
                                # Inhalation PBPK Model #
#=====================================================================#

code <- '

$PROB
Author          : ARK
Affiliation     : PMI R&D, Switzerland
Date            : April 2020
Compound        : CQ and HCQ
Description     : Inhalation PBPK model

$SET
delta= 0, atol=0, rtol=0, maxsteps=0,

$PARAM @annotated
// Compound Properties
BP              : 0 : blood plasma ratio
Fu              : 0 : protein unbound fraction
MW              : 0 : molecular weight
logKow          : 0 :  logP
pKa1            : 0 : pKa1
valency1        : 0 : valency
pKa2            : 0 : pKa2
valency2        : 0 : valency
Faraday         : 0 : Faraday
R               : 0 : Real Gas Constant
Temp            : 0 : Temperature
deltaS          : 0 : Diffusivity factor

// Metabolsim and clearance parameters //
KA              : 0 : absorption rate for oral dose
Fabs            : 0 : absorbed fractions for oral
DC              : 0 : diffusion coefficient
Vmaxrenal       : 0 : Kidney clearance
Kmrenal         : 0 : Kidney clearance
GFR             : 0 : Kidney clearance
CL_Liv          : 0 : Clearance liver
Vmaxliver       : 0 : Liver clearance
Kmliver         : 0 : Liver clearance
Vmaxpgp         : 0 : Pgp transporter
Kmpgp           : 0 : Pgp transporter
Vmaxpgp1        : 0 : 0 for humans
Kmpgp1          : 0 :

// Physiological parameters
BW              : 0 : body weight
```

```
170     Qc              : 0 : total cardiac output
171     Cyt_Ionstrength: 0 : cytosol ion strength
172     Cyt_E           : 0 : cytosol membrane potential
173     Lys_Ionstrength: 0 : lysosome ion strength
174     Lys_E           : 0 : lys membrane potential
175     Cyt_pHoutside  : 0 : extracellular pH
176     Cyt_pHinside    : 0 : intracellular pH
177     Lys_pHinside    : 0 : lysosomal pH
178     Mucus_pHoutside : 0 : Mucus pH
179     Inter_pHoutside : 0 : lung interstial pH
180     Cyt_pHinside_AUATissue : 0 : lung cellular pH
181     Cyt_pHinside_ACATissue : 0 : same as UA
182     Cyt_pHinside_ATATissue : 0 : same as UA
183     Cyt_pHinside_APATissue : 0 : same as UA
184     Lys_pHinside_lung : 0 : lung lysosomal pH
185     Cyt_lipid       : 0 : cytosolic lipid
186     Lys_lipid       : 0 : lysosomal lipid
187     Cyt_water       : 0 : cytosol water
188     Lys_water       : 0 : lysosomal water
189     Cyt_Diameter    : 0 : cytosol diameter
190     Lys_Diameter    : 0 : lysosmal diameter
191     Lys_buff        : 0 : lys buffer capacity
192
193     // Percent Blood Flows (%CO)
194     Qbrain_perc     : 0 :
195     Qheart_perc     : 0 :
196     Qkid_perc       : 0 :
197     Qskin_perc      : 0 :
198     Qgi_perc        : 0 :
199     Qspleen_perc    : 0 :
200     Qliver_perc     : 0 :
201     Qairway_perc    : 0 :
202     Qmuscle_perc    : 0 :
203     Qarterial_perc  : 0 :
204     Qvenous_perc    : 0 :
205     Qslow_perc      : 0 : fat + bone
206     Qua_perc        : 0 :
207     Qca_perc        : 0 :
208     Qta_perc        : 0 :
209
210     // Percent Tissue Volumes
211     Vbrain_perc     : 0 :
212     Vheart_perc     : 0 :
213     Vkid_perc       : 0 :
214     Vskin_perc      : 0 :
215     Vgi_perc        : 0 :
216     Vspleen_perc    : 0 :
217     Vliver_perc     : 0 :
218     Vairway_perc    : 0 :
219     Vmuscle_perc    : 0 :
220     Varterial_perc  : 0 :
```

```
221     Vvenous_perc   : 0 :
222     Vslow_perc     : 0 : fat + bone
223     Vairway_fVas   : 0 : airway vascular percent
224     Vairway_fEW    : 0 : airway interstitial percent
225
226     // Percent Lysosomal volume
227     Vbrainlys_perc          : 0 :
228     Vheartlys_perc          : 0 :
229     Vkidlys_perc            : 0 :
230     Vgilys_perc             : 0 :
231     Vspleenlys_perc         : 0 :
232     Vliverlys_perc          : 0 :
233     VPAairwaylys_perc       : 0 :  PA
234     Vairwaylys_perc         : 0 :  UA, CA, TA
235     Vmusclelys_perc         : 0 :
236     Vskinlys_perc           : 0 :
237     Vslowlys_perc           : 0 :
238     Votherlys_perc          : 0 :
239
240     // Partition Coefficients
241     PNbrain                 : 0 :
242     PNheart                 : 0 :
243     PNkid                   : 0 :
244     PNskin                  : 0 :
245     PNgut                   : 0 :
246     PNspleen                : 0 :
247     PNliv                   : 0 :
248     PNlung                  : 0 :
249     PNmuscle                : 0 :
250     PNslow                  : 0 :
251     PNother                 : 0 :
252
253     //Respiratory tract parameters
254     tdur            : 0 : iv infusion time
255     puffdur         : 0 : puff duration
256     KmucUA          : 0 :  MCC UA
257     KmucCA          : 0 : MCC CA
258     KmucTA          : 0 : MCC TA
259     SA_UA           : 0 : surface area UA
260     SA_CA           : 0 : surface area CA
261     SA_TA           : 0 : surface area TA
262     SA_PA           : 0 : surface area PA
263     TUA_mucus       : 0 : Thickness mucus
264     TCA_mucus       : 0 :
265     TTA_mucus       : 0 :
266     TPA_mucus       : 0 :
267     TUA_pcl         : 0 : thickness periciliary layer
268     TCA_pcl         : 0 :
269     TTA_pcl         : 0 :
270     TUA_tiss        : 0 : thickness cellular layer
271     TCA_tiss        : 0 :
```

```
TTA_tiss        : 0 :
TPA_tiss        : 0 :

$MAIN
D_Avenous = tdur;
D_AUAmucus = puffdur;
D_ACAmucus = puffdur;
D_ATAmucus = puffdur;
D_APAmucus = puffdur;
F_GUT = Fabs;
double BP_Fu = BP/Fu;

//Volume of mucus
double VUA_mucus = TUA_mucus * SA_UA;
double VCA_mucus = TCA_mucus * SA_CA;
double VTA_mucus = TTA_mucus * SA_TA;
double VPA_mucus = TPA_mucus * SA_PA;

//Volume of periciliary layer
double VUA_asl = TUA_pcl * SA_UA;
double VCA_asl = TCA_pcl * SA_CA;
double VTA_asl = TTA_pcl * SA_TA;
double VPA_asl = 0; //no pcl in PA region so 0

//Volume of tissue or cellular layer
double VUAtiss = TUA_tiss  * SA_UA;
double VCAtiss = TCA_tiss * SA_CA;
double VTAtiss = TTA_tiss * SA_TA;
double VPAtiss = TPA_tiss * SA_PA;
double totalLung = VUA_mucus + VCA_mucus + VTA_mucus + VPA_mucus + VUA_asl + VCA_asl +
VTA_asl + VPA_asl + VUAtiss + VCAtiss + VTAtiss + VPAtiss;

//Volume of Vascular space
double VUAtiss_Vas = Vairway_fVas * VUAtiss/100;
double VCAtiss_Vas = Vairway_fVas * VCAtiss/100;
double VTAtiss_Vas = Vairway_fVas * VTAtiss/100;
double VPAtiss_Vas = Vairway_fVas * VPAtiss/100;

//Volume of interstitial space
double VUAtiss_EW = Vairway_fEW * VUAtiss/100;
double VCAtiss_EW = Vairway_fEW * VCAtiss/100;
double VTAtiss_EW = Vairway_fEW * VTAtiss/100;
double VPAtiss_EW = Vairway_fEW * VPAtiss/100;

// Blood flow rates
double Qua = Qua_perc*Qc/100;
double Qca = Qca_perc*Qc/100;
double Qta = Qta_perc*Qc/100;
double Qbrain = Qbrain_perc*Qc/100;
double Qheart = Qheart_perc*Qc/100;
double Qkid = Qkid_perc*Qc/100;
```

```
323     double Qgi = Qgi_perc*Qc/100;
324     double Qspleen = Qspleen_perc*Qc/100;
325     double Qliver = Qliver_perc*Qc/100;
326     double Qairway = Qairway_perc*Qc/100;
327     double Qmuscle = Qmuscle_perc * Qc/100;
328     double Qskin = Qskin_perc*Qc/100;
329     double Qslow = Qslow_perc*Qc/100;
330     double Qother = (100 - (Qua_perc + Qca_perc + Qta_perc + Qbrain_perc + Qheart_perc + Qkid_perc +
331     Qskin_perc+Qgi_perc+Qspleen_perc+Qliver_perc + Qmuscle_perc + Qslow_perc)) * Qc/100;
332
333     // Volume of tissue
334     double Vbrain = Vbrain_perc*BW/100;
335     double Vheart = Vheart_perc*BW/100;
336     double Vkid = Vkid_perc*BW/100;
337     double Vgi = Vgi_perc*BW/100;
338     double Vspleen = Vspleen_perc*BW/100;
339     double Vliver = Vliver_perc*BW/100;
340     double Vairway = Vairway_perc*BW/100;
341     double Vmuscle = Vmuscle_perc*BW/100;
342     double Vskin = Vskin_perc*BW/100;
343     double Vslow    = Vslow_perc*BW/100;
344     double Varterial= Varterial_perc*BW/100;
345     double Vvenous = Vvenous_perc*BW/100;
346     double Vother   = - totalLung + (100 - (Vbrain_perc  + Vheart_perc  + Vkid_perc  + Vgi_perc  +
347     Vspleen_perc + Vliver_perc  + Vmuscle_perc  + Vskin_perc  + Vslow_perc  + Varterial_perc  +
348     Vvenous_perc ))*BW/100 ;
349
350     // Volume of lysosome
351     double Vbrainlys = Vbrain*Vbrainlys_perc/100;
352     double Vheartlys = Vheart*Vheartlys_perc/100;
353     double Vkidlys  = Vkid*Vkidlys_perc/100;
354     double Vgilys = Vgi*Vgilys_perc/100;
355     double Vspleenlys= Vspleen*Vspleenlys_perc/100;
356     double Vliverlys = Vliver* Vliverlys_perc/100;
357     double VPAairwaylys = Vairway*VPAairwaylys_perc/100;
358     double Vairwaylys = Vairway*Vairwaylys_perc/100;
359     double Vmusclelys = Vmuscle*Vmusclelys_perc/100;
360     double Vskinlys = Vskin*Vskinlys_perc/100;
361     double Vslowlys = Vslow*Vslowlys_perc/100;
362     double Votherlys = Vother*Votherlys_perc/100;
363     double VUAtisslys = VUAtiss*Vairwaylys_perc/100;
364     double VCAtisslys = VUAtiss*Vairwaylys_perc/100;
365     double VTAtisslys = VUAtiss*Vairwaylys_perc/100;
366     double VPAtisslys = VUAtiss*VPAairwaylys_perc/100;
367
368     // volume and surface area
369     double Vlysosome = (4/3) * 3.14 * pow(Lys_Diameter/2, 3);
370     double SAlysbrain = Vbrain * Vbrainlys * (4)*3.14 * pow(Lys_Diameter/2, 2) / Vlysosome;
371     double SAlysheart = Vheart * Vheartlys * (4)*3.14 * pow(Lys_Diameter/2, 2) / Vlysosome;
372     double SAlyskid = Vkid* Vkidlys * (4)*3.14 * pow(Lys_Diameter/2, 2) / Vlysosome;
373     double SAlysgi = Vgi* Vgilys * (4)*3.14 * pow(Lys_Diameter/2, 2) / Vlysosome;
```

```
374 double SAlysspleen= Vspleen* Vspleenlys * (4)*3.14 * pow(Lys_Diameter/2, 2) / Vlysosome;
375 double SAlysliver = Vliver * Vliverlys * (4)*3.14 * pow(Lys_Diameter/2, 2) / Vlysosome;
376 double SAlysairway = Vairway* Vairwaylys * (4)*3.14 * pow(Lys_Diameter/2, 2) / Vlysosome;
377 double SAlysmuscle= Vmuscle* Vmusclelys * (4)*3.14 * pow(Lys_Diameter/2, 2) / Vlysosome;
378 double SAlysskin = Vskin* Vskinlys* (4)*3.14 * pow(Lys_Diameter/2, 2) / Vlysosome;
379 double SAlysslow = Vslow* Vslowlys* (4)*3.14 * pow(Lys_Diameter/2, 2) / Vlysosome;
380 double SAlysother = Vother * Votherlys* (4)*3.14 * pow(Lys_Diameter/2, 2) / Vlysosome;
381 double SAlysUAtiss = VUAtiss * Vairwaylys * (4)*3.14 * pow(Lys_Diameter/2, 2) / Vlysosome;
382 double SAlysCAtiss = VCAtiss * Vairwaylys * (4)*3.14 * pow(Lys_Diameter/2, 2) / Vlysosome;
383 double SAlysTAtiss = VTAtiss * Vairwaylys * (4)*3.14 * pow(Lys_Diameter/2, 2) / Vlysosome;
384 double SAlysPAtiss = VPAtiss * VPAairwaylys * (4)*3.14 * pow(Lys_Diameter/2, 2) / Vlysosome;
385
386 $INIT @annotated
387 Aarterial       : 0 :
388 Avenous        : 0 :
389 Abrain         : 0 :
390 Agi            : 0 :
391 Aspleen        : 0 :
392 Aheart         : 0 :
393 Akid           : 0 :
394 Aliver         : 0 :
395 Amuscle        : 0 :
396 Askin          : 0 :
397 Aslow          : 0 :
398 Aother         : 0 :
399 Abrainlys      : 0 :
400 Agilys         : 0 :
401 Aspleenlys     : 0 :
402 Aheartlys      : 0 :
403 Akidlys        : 0 :
404 Aliverlys      : 0 :
405 Amusclelys     : 0 :
406 Askinlys       : 0 :
407 Aslowlys       : 0 :
408 Aotherlys      : 0 :
409 AUAmucus       : 0 : mucus levels in UA
410 ACAmucus       : 0 :
411 ATAmucus       : 0 :
412 APAmucus       : 0 :
413 AUAasl         : 0 : periciliary layer upper lung
414 ACAasl         : 0 :
415 ATAasl         : 0 :
416 AUATissuelys   : 0 : lysosomal upper airway
417 AUATissue      : 0 :
418 ACATissuelys   : 0 :
419 ACATissue      : 0 : cellular upper airway
420 ATATissuelys   : 0 :
421 ATATissue      : 0 :
422 APATissuelys   : 0 :
423 APATissue      : 0 :
424 AUATissueinter : 0 : interstitial UA
```

```
AUATissuevascular : 0 : vascular UA
ACATissueinter : 0 :
ACATissuevascular : 0 :
ATATissueinter          : 0 :
ATATissuevascular       : 0 :
APATissueinter          : 0 :
APATissuevascular       : 0 :
GUT                     : 0 :

$ODE
// Lysosomal buffering
double Lys_pHinside_Abrainlys   = Lys_pHinside + (Abrainlys/Lys_buff);
double Lys_pHinside_Agilys = Lys_pHinside + (Agilys/Lys_buff);
double Lys_pHinside_Aspleenlys = Lys_pHinside + (Aspleenlys/Lys_buff);
double Lys_pHinside_Akidlys = Lys_pHinside + (Akidlys/Lys_buff);
double Lys_pHinside_Aliverlys = Lys_pHinside + (Aliverlys/Lys_buff);
double Lys_pHinside_AUATissuelys = Lys_pHinside_lung + (AUATissuelys/Lys_buff);
double Lys_pHinside_ACATissuelys = Lys_pHinside_lung + (ACATissuelys/Lys_buff);
double Lys_pHinside_ATATissuelys = Lys_pHinside_lung + (ATATissuelys/Lys_buff);
double Lys_pHinside_APATissuelys = Lys_pHinside_lung + (APATissuelys/Lys_buff);
double Lys_pHinside_Aheartlys = Lys_pHinside + (Aheartlys/Lys_buff);
double Lys_pHinside_Askinlys = Lys_pHinside + (Askinlys/Lys_buff);
double Lys_pHinside_Amusclelys = Lys_pHinside + (Amusclelys/Lys_buff);
double Lys_pHinside_Aslowlys    = Lys_pHinside + (Aslowlys/Lys_buff);
double Lys_pHinside_Aotherlys = Lys_pHinside + (Aotherlys/Lys_buff);
double Kow  = pow(10, logKow);
double logKowdiss = logKow - (fabs(valency1)) * 6.5;
double Kowdiss    = pow(10, logKowdiss);
double logKowdiss2= logKow-(fabs(valency2)) *6.5;
double Kowdiss2   = pow(10, logKowdiss2);
double Pn  = pow(10, logKow - deltaS);
double Pd1 = pow(10, logKowdiss - deltaS);
double Pd2 = pow(10, logKowdiss2 - deltaS);
// fractions outside
double fnout  = 1/(1+ pow(10, pKa1-Cyt_pHoutside) + pow(10, (pKa1-Cyt_pHoutside)+(pKa2-
Cyt_pHoutside))) ;
double fd1out = fnout * pow(10, (pKa1-Cyt_pHoutside)+(pKa2-Cyt_pHoutside));
double fd2out = fnout * pow(10, pKa1-Cyt_pHoutside);
double D1out = fd1out/fnout;
double D2out = fd2out/fnout;
double gamman  = pow(10, 0.3*Cyt_Ionstrength);
double gammad1 = pow(10, (-0.5*valency1*valency1*(sqrt(Cyt_Ionstrength)/(1+sqrt(Cyt_Ionstrength))-
0.3*Cyt_Ionstrength)));
double gammad2 = pow(10, (-0.5*valency2*valency2*(sqrt(Cyt_Ionstrength)/(1+sqrt(Cyt_Ionstrength))-
0.3*Cyt_Ionstrength)));
// Fractions lung interstitial
double fninter  = 1/(1+ pow(10, pKa1-Inter_pHoutside) + pow(10, (pKa1-Inter_pHoutside)+(pKa2-
Inter_pHoutside))) ; //activity of neutral molecule
double fd1inter = fninter * pow(10, (pKa1-Inter_pHoutside)+(pKa2-Inter_pHoutside));
double fd2inter = fninter * pow(10, pKa1-Inter_pHoutside);
double D1inter = fd1inter/fninter;
```

```
476     double D2inter = fd2inter/fninter;
477     // fractions in mucus
478     double fnmucus  = 1/(1+ pow(10, pKa1-Mucus_pHoutside) + pow(10, (pKa1-Mucus_pHoutside)+(pKa2-
479     Mucus_pHoutside))) ; //activity of neutral molecule
480     double fd1mucus = fnmucus * pow(10, (pKa1-Mucus_pHoutside)+(pKa2-Mucus_pHoutside));
481     double fd2mucus = fnmucus * pow(10, pKa1-Mucus_pHoutside);
482     double D1mucus = fd1mucus/fnmucus;
483     double D2mucus = fd2mucus/fnmucus;
484     // Brain
485     double Kncyt_brain  = 1.22 * Cyt_lipid * Kow;
486     double Kd1cyt_brain = 1.22 * Cyt_lipid * Kowdiss;
487     double Kd2cyt_brain = 1.22 * Cyt_lipid * Kowdiss2;
488     double ancyt_brain  = 1/(1+ pow(10, pKa1-Cyt_pHinside) + pow(10, (pKa1-Cyt_pHinside)+(pKa2-
489     Cyt_pHinside)));
490     double ad2cyt_brain = ancyt_brain * pow(10, (pKa1-Cyt_pHinside)+(pKa2-Cyt_pHinside));
491     double ad1cyt_brain = ancyt_brain * pow(10, pKa1-Cyt_pHinside);
492     double D1cyt_brain = ad1cyt_brain/ancyt_brain;
493     double D2cyt_brain = ad2cyt_brain/ancyt_brain;
494     double fncyt_brain  = 1/(Cyt_water/gamman + Kncyt_brain/gamman + D2cyt_brain*Cyt_water/gammad2
495     + D2cyt_brain*Kd2cyt_brain/gammad2 + D1cyt_brain*Cyt_water/gammad1 +
496     D1cyt_brain*Kd1cyt_brain/gammad1);
497     double fd1cyt_brain =  fncyt_brain * D1cyt_brain;
498     double fd2cyt_brain =  fncyt_brain * D2cyt_brain;
499     double Nd1cyt_brain = valency1*(Cyt_E)*Faraday/(R*Temp);
500     double Nd2cyt_brain = valency2*(Cyt_E)*Faraday/(R*Temp);
501     double Joutcytbrain = fnout*Pn + fd2out*Pd2* Nd2cyt_brain/(exp(Nd2cyt_brain)-1) + fd1out*
502     Pd1*Nd1cyt_brain/(exp (Nd1cyt_brain)-1);
503     double Jcytoutbrain = fncyt_brain*Pn +
504     fd1cyt_brain*Pd1*exp(Nd1cyt_brain)*Nd1cyt_brain/(exp(Nd1cyt_brain)-1) + fd2cyt_brain*Pd2*
505     exp(Nd2cyt_brain)*Nd2cyt_brain/(exp(Nd2cyt_brain)-1);
506     double Knlys_brain  = 1.22 * Lys_lipid * Kow;
507     double Kd1lys_brain = 1.22 * Lys_lipid * Kowdiss;
508     double Kd2lys_brain = 1.22 * Lys_lipid * Kowdiss2;
509     double anlys_brain  = 1/(1+ pow(10, pKa1-Lys_pHinside_Abrainlys) + pow(10, (pKa1-
510     Lys_pHinside_Abrainlys)+(pKa2-Lys_pHinside_Abrainlys)));
511     double ad2lys_brain = anlys_brain * pow(10, (pKa1-Lys_pHinside_Abrainlys)+(pKa2-
512     Lys_pHinside_Abrainlys));
513     double ad1lys_brain = anlys_brain * pow(10, pKa1-Lys_pHinside_Abrainlys);
514     double D1lys_brain = ad1lys_brain/anlys_brain;
515     double D2lys_brain = ad2lys_brain/anlys_brain;
516     double fnlys_brain  = 1/(Lys_water/gamman + Knlys_brain/gamman + D2lys_brain*Lys_water/gammad2
517     + D2lys_brain*Kd2lys_brain/gammad2 + D1lys_brain*Lys_water/gammad1 +
518     D1lys_brain*Kd1lys_brain/gammad1);
519     double fd1lys_brain =  fnlys_brain * D1lys_brain;
520     double fd2lys_brain =  fnlys_brain * D2lys_brain;
521     double Nd1lys_brain = valency1*Lys_E*Faraday/(R*Temp);
522     double Nd2lys_brain = valency2*Lys_E*Faraday/(R*Temp);
523     double Jbrainlys = fncyt_brain*Pn + fd2cyt_brain*Pd2*Nd2lys_brain/(exp(Nd2lys_brain)-1) +
524     fd1cyt_brain*Pd1*Nd1lys_brain/(exp(Nd1lys_brain)-1);
```

```
525     double Jlysbrain = fnlys_brain*Pn +
526     fd1lys_brain*Pd1*exp(Nd1lys_brain)*Nd1lys_brain/(exp(Nd1lys_brain)-1) +
527     fd2lys_brain*Pd2*exp(Nd2lys_brain)*Nd2lys_brain/(exp(Nd2lys_brain)-1);
528     // Heart
529     double Kncyt_heart  = 1.22 * Cyt_lipid * Kow;
530     double Kd1cyt_heart = 1.22 * Cyt_lipid * Kowdiss;
531     double Kd2cyt_heart = 1.22 * Cyt_lipid * Kowdiss2;
532     double ancyt_heart  = 1/(1+ pow(10, pKa1-Cyt_pHinside) + pow(10, (pKa1-Cyt_pHinside)+(pKa2-
533     Cyt_pHinside)));
534     double ad2cyt_heart = ancyt_heart * pow(10, (pKa1-Cyt_pHinside)+(pKa2-Cyt_pHinside));
535     double ad1cyt_heart = ancyt_heart * pow(10, pKa1-Cyt_pHinside);
536     double D1cyt_heart  = ad1cyt_heart/ancyt_heart;
537     double D2cyt_heart  = ad2cyt_heart/ancyt_heart;
538     double fncyt_heart  = 1/(Cyt_water/gamman + Kncyt_heart/gamman + D2cyt_heart*Cyt_water/gammad2
539     + D2cyt_heart*Kd2cyt_heart/gammad2 + D1cyt_heart*Cyt_water/gammad1 +
540     D1cyt_heart*Kd1cyt_heart/gammad1);
541     double fd1cyt_heart = fncyt_heart * D1cyt_heart;
542     double fd2cyt_heart = fncyt_heart * D2cyt_heart;
543     double Nd1cyt_heart = valency1*(Cyt_E)*Faraday/(R*Temp);
544     double Nd2cyt_heart = valency2*(Cyt_E)*Faraday/(R*Temp);
545     double Joutcytheart = fnout*Pn + fd2out*Pd2*Nd2cyt_heart/(exp(Nd2cyt_heart)-1) +
546     fd1out*Pd1*Nd1cyt_heart/(exp(Nd1cyt_heart)-1);
547     double Jcytoutheart = fncyt_heart*Pn +
548     fd1cyt_heart*Pd1*exp(Nd1cyt_heart)*Nd1cyt_heart/(exp(Nd1cyt_heart)-1) +
549     fd2cyt_heart*Pd2*exp(Nd2cyt_heart)*Nd2cyt_heart/(exp(Nd2cyt_heart)-1);
550     double Knlys_heart  = 1.22 * (Lys_lipid ) * (Kow);
551     double Kd1lys_heart = 1.22 * (Lys_lipid ) * (Kowdiss);
552     double Kd2lys_heart = 1.22 * (Lys_lipid ) * (Kowdiss2);
553     double anlys_heart  = 1/(1+ pow(10, pKa1-Lys_pHinside_Aheartlys) + pow(10, (pKa1-
554     Lys_pHinside_Aheartlys)+(pKa2-Lys_pHinside_Aheartlys)));
555     double ad2lys_heart = anlys_heart * pow(10, (pKa1-Lys_pHinside_Aheartlys)+(pKa2-
556     Lys_pHinside_Aheartlys));
557     double ad1lys_heart = anlys_heart * pow(10, pKa1-Lys_pHinside_Aheartlys);
558     double D1lys_heart  = ad1lys_heart/anlys_heart;
559     double D2lys_heart  = ad2lys_heart/anlys_heart;
560     double fnlys_heart  = 1/((Lys_water)/gamman + Knlys_heart/gamman +
561     D2lys_heart*(Lys_water)/gammad2 + D2lys_heart*Kd2lys_heart/gammad2 +
562     D1lys_heart*(Lys_water)/gammad1 + D1lys_heart*Kd1lys_heart/gammad1);
563     double fd1lys_heart =  fnlys_heart * D1lys_heart;
564     double fd2lys_heart =  fnlys_heart * D2lys_heart;
565     double Nd1lys_heart = valency1*(Lys_E )*Faraday/(R*Temp);
566     double Nd2lys_heart = valency2*(Lys_E )*Faraday/(R*Temp);
567     double Jheartlys = fncyt_heart*Pn + fd2cyt_heart*Pd2*Nd2lys_heart/(exp(Nd2lys_heart)-1) +
568     fd1cyt_heart*Pd1*Nd1lys_heart/(exp(Nd1lys_heart)-1);
569     double Jlysheart = fnlys_heart*Pn +
570     fd1lys_heart*Pd1*exp(Nd1lys_heart)*Nd1lys_heart/(exp(Nd1lys_heart)-1) +
571     fd2lys_heart*Pd2*exp(Nd2lys_heart)*Nd2lys_heart/(exp(Nd2lys_heart)-1);
572     // Kidney
573     double Kncyt_kid  = 1.22 * Cyt_lipid * Kow;
574     double Kd1cyt_kid = 1.22 * Cyt_lipid * Kowdiss;
575     double Kd2cyt_kid = 1.22 * Cyt_lipid * Kowdiss2;
```

```
576     double ancyt_kid  = 1/(1+ pow(10, pKa1-Cyt_pHinside) + pow(10, (pKa1-Cyt_pHinside)+(pKa2-
577     Cyt_pHinside)));
578     double ad2cyt_kid = ancyt_kid * pow(10, (pKa1-Cyt_pHinside)+(pKa2-Cyt_pHinside));
579     double ad1cyt_kid = ancyt_kid * pow(10, pKa1-Cyt_pHinside);
580     double D1cyt_kid = ad1cyt_kid/ancyt_kid;
581     double D2cyt_kid = ad2cyt_kid/ancyt_kid;
582     double fncyt_kid  = 1/(Cyt_water/gamman + Kncyt_kid/gamman + D2cyt_kid*Cyt_water/gammad2 +
583     D2cyt_kid*Kd2cyt_kid/gammad2 + D1cyt_kid*Cyt_water/gammad1 +
584     D1cyt_kid*Kd1cyt_kid/gammad1);
585     double fd1cyt_kid =  fncyt_kid * D1cyt_kid;
586     double fd2cyt_kid =  fncyt_kid * D2cyt_kid;
587     double Nd1cyt_kid = valency1*Cyt_E*Faraday/(R*Temp);
588     double Nd2cyt_kid = valency2*Cyt_E*Faraday/(R*Temp);
589     double Joutcytkid = fnout*Pn + fd2out*Pd2*Nd2cyt_kid/(exp(Nd2cyt_kid)-1) +
590     fd1out*Pd1*Nd1cyt_kid/(exp(Nd1cyt_kid)-1);
591     double Jcytoutkid = fncyt_kid*Pn + fd1cyt_kid*Pd1*exp(Nd1cyt_kid)*Nd1cyt_kid/(exp(Nd1cyt_kid)-1)
592     + fd2cyt_kid*Pd2*exp(Nd2cyt_kid)*Nd2cyt_kid/(exp(Nd2cyt_kid)-1);
593     double Knlys_kid  = 1.22 * Lys_lipid * Kow;
594     double Kd1lys_kid = 1.22 * Lys_lipid * Kowdiss;
595     double Kd2lys_kid = 1.22 * Lys_lipid * Kowdiss2;
596     double anlys_kid  = 1/(1+ pow(10, pKa1-Lys_pHinside_Akidlys) + pow(10, (pKa1-
597     Lys_pHinside_Akidlys)+(pKa2-Lys_pHinside_Akidlys)));
598     double ad2lys_kid = anlys_kid * pow(10, (pKa1-Lys_pHinside_Akidlys)+(pKa2-
599     Lys_pHinside_Akidlys));
600     double ad1lys_kid = anlys_kid * pow(10, pKa1-Lys_pHinside_Akidlys);
601     double D1lys_kid = ad1lys_kid/anlys_kid;
602     double D2lys_kid = ad2lys_kid/anlys_kid;
603     double fnlys_kid  = 1/(Lys_water/gamman + Knlys_kid/gamman + D2lys_kid*Lys_water/gammad2 +
604     D2lys_kid*Kd2lys_kid/gammad2 + D1lys_kid*Lys_water/gammad1 +
605     D1lys_kid*Kd1lys_kid/gammad1);
606     double fd1lys_kid =  fnlys_kid * D1lys_kid;
607     double fd2lys_kid =  fnlys_kid * D2lys_kid;
608     double Nd1lys_kid = valency1*Lys_E*Faraday/(R*Temp);
609     double Nd2lys_kid = valency2*Lys_E*Faraday/(R*Temp);
610     double Jkidlys = fncyt_kid*Pn + fd2cyt_kid*Pd2*Nd2lys_kid/(exp(Nd2lys_kid)-1) +
611     fd1cyt_kid*Pd1*Nd1lys_kid/(exp(Nd1lys_kid)-1);
612     double Jlyskid = fnlys_kid*Pn + fd1lys_kid*Pd1*exp(Nd1lys_kid)*Nd1lys_kid/(exp(Nd1lys_kid)-1) +
613     fd2lys_kid*Pd2*exp(Nd2lys_kid)*Nd2lys_kid/(exp(Nd2lys_kid)-1);
614     // Liver
615     double Kncyt_liver  = 1.22 * Cyt_lipid * Kow;
616     double Kd1cyt_liver = 1.22 * Cyt_lipid * Kowdiss;
617     double Kd2cyt_liver = 1.22 * Cyt_lipid * Kowdiss2;
618     double ancyt_liver  = 1/(1+ pow(10, pKa1-Cyt_pHinside) + pow(10, (pKa1-Cyt_pHinside)+(pKa2-
619     Cyt_pHinside)));
620     double ad2cyt_liver = ancyt_liver * pow(10, (pKa1-Cyt_pHinside)+(pKa2-Cyt_pHinside));
621     double ad1cyt_liver = ancyt_liver * pow(10, pKa1-Cyt_pHinside);
622     double D1cyt_liver = ad1cyt_liver/ancyt_liver;
623     double D2cyt_liver = ad2cyt_liver/ancyt_liver;
624     double fncyt_liver  = 1/(Cyt_water/gamman + Kncyt_liver/gamman + D2cyt_liver*Cyt_water/gammad2
625     + D2cyt_liver*Kd2cyt_liver/gammad2 + D1cyt_liver*Cyt_water/gammad1 +
626     D1cyt_liver*Kd1cyt_liver/gammad1);
```

```
627     double fd1cyt_liver =  fncyt_liver * D1cyt_liver;
628     double fd2cyt_liver =  fncyt_liver * D2cyt_liver;
629     double Nd1cyt_liver = valency1*Cyt_E*Faraday/(R*Temp);
630     double Nd2cyt_liver = valency2*Cyt_E*Faraday/(R*Temp);
631     double Joutcytliver = fnout*Pn + fd2out*Pd2*Nd2cyt_liver/(exp(Nd2cyt_liver)-1) +
632     fd1out*Pd1*Nd1cyt_liver/(exp(Nd1cyt_liver)-1);
633     double Jcytoutliver = fncyt_liver*Pn +
634     fd1cyt_liver*Pd1*exp(Nd1cyt_liver)*Nd1cyt_liver/(exp(Nd1cyt_liver)-1) +
635     fd2cyt_liver*Pd2*exp(Nd2cyt_liver)*Nd2cyt_liver/(exp(Nd2cyt_liver)-1);
636     double Knlys_liver  = 1.22 * Lys_lipid * Kow;
637     double Kd1lys_liver = 1.22 * Lys_lipid * Kowdiss;
638     double Kd2lys_liver = 1.22 * Lys_lipid * Kowdiss2;
639     double anlys_liver  = 1/(1+ pow(10, pKa1-Lys_pHinside_Aliverlys) + pow(10, (pKa1-
640     Lys_pHinside_Aliverlys)+(pKa2-Lys_pHinside_Aliverlys)));
641     double ad2lys_liver = anlys_liver * pow(10, (pKa1-Lys_pHinside_Aliverlys)+(pKa2-
642     Lys_pHinside_Aliverlys));
643     double ad1lys_liver = anlys_liver * pow(10, pKa1-Lys_pHinside_Aliverlys);
644     double D1lys_liver = ad1lys_liver/anlys_liver;
645     double D2lys_liver = ad2lys_liver/anlys_liver;
646     double fnlys_liver  = 1/(Lys_water/gamman + Knlys_liver/gamman + D2lys_liver*Lys_water/gammad2 +
647     D2lys_liver*Kd2lys_liver/gammad2 + D1lys_liver*Lys_water/gammad1 +
648     D1lys_liver*Kd1lys_liver/gammad1);
649     double fd1lys_liver =  fnlys_liver * D1lys_liver;
650     double fd2lys_liver =  fnlys_liver * D2lys_liver;
651     double Nd1lys_liver = valency1*Lys_E*Faraday/(R*Temp);
652     double Nd2lys_liver = valency2*Lys_E*Faraday/(R*Temp);
653     double Jliverlys = fncyt_liver*Pn + fd2cyt_liver*Pd2*Nd2lys_liver/(exp(Nd2lys_liver)-1) +
654     fd1cyt_liver*Pd1*Nd1lys_liver/(exp(Nd1lys_liver)-1);
655     double Jlysliver = fnlys_liver*Pn +
656     fd1lys_liver*Pd1*exp(Nd1lys_liver)*Nd1lys_liver/(exp(Nd1lys_liver)-1) +
657     fd2lys_liver*Pd2*exp(Nd2lys_liver)*Nd2lys_liver/(exp(Nd2lys_liver)-1);
658     // GI
659     double Kncyt_gi  = 1.22 * Cyt_lipid * Kow;
660     double Kd1cyt_gi = 1.22 * Cyt_lipid * Kowdiss;
661     double Kd2cyt_gi = 1.22 * Cyt_lipid * Kowdiss2;
662     double ancyt_gi  = 1/(1+ pow(10, pKa1-Cyt_pHinside) + pow(10, (pKa1-Cyt_pHinside)+(pKa2-
663     Cyt_pHinside)));
664     double ad2cyt_gi = ancyt_gi * pow(10, (pKa1-Cyt_pHinside)+(pKa2-Cyt_pHinside));
665     double ad1cyt_gi = ancyt_gi * pow(10, pKa1-Cyt_pHinside);
666     double D1cyt_gi = ad1cyt_gi/ancyt_gi;
667     double D2cyt_gi = ad2cyt_gi/ancyt_gi;
668     double fncyt_gi  = 1/(Cyt_water/gamman + Kncyt_gi/gamman + D2cyt_gi*Cyt_water/gammad2 +
669     D2cyt_gi*Kd2cyt_gi/gammad2 + D1cyt_gi*Cyt_water/gammad1 + D1cyt_gi*Kd1cyt_gi/gammad1);
670     double fd1cyt_gi =  fncyt_gi * D1cyt_gi;
671     double fd2cyt_gi =  fncyt_gi * D2cyt_gi;
672     double Nd1cyt_gi = valency1*Cyt_E*Faraday/(R*Temp);
673     double Nd2cyt_gi = valency2*Cyt_E*Faraday/(R*Temp);
674     double Joutcytgi = fnout*Pn + fd2out*Pd2*Nd2cyt_gi/(exp(Nd2cyt_gi)-1) +
675     fd1out*Pd1*Nd1cyt_gi/(exp(Nd1cyt_gi)-1);
676     double Jcytoutgi = fncyt_gi*Pn + fd1cyt_gi*Pd1*exp(Nd1cyt_gi)*Nd1cyt_gi/(exp(Nd1cyt_gi)-1) +
677     fd2cyt_gi*Pd2*exp(Nd2cyt_gi)*Nd2cyt_gi/(exp(Nd2cyt_gi)-1);
```

```
678     double Knlys_gi  = 1.22 * Lys_lipid * Kow;
679     double Kd1lys_gi = 1.22 * Lys_lipid * Kowdiss;
680     double Kd2lys_gi = 1.22 * Lys_lipid * Kowdiss2;
681     double anlys_gi  = 1/(1+ pow(10, pKa1-Lys_pHinside_Agilys) + pow(10, (pKa1-
682     Lys_pHinside_Agilys)+(pKa2-Lys_pHinside_Agilys)));
683     double ad2lys_gi = anlys_gi * pow(10, (pKa1-Lys_pHinside_Agilys)+(pKa2-Lys_pHinside_Agilys));
684     double ad1lys_gi = anlys_gi * pow(10, pKa1-Lys_pHinside_Agilys);
685     double D1lys_gi = ad1lys_gi/anlys_gi;
686     double D2lys_gi = ad2lys_gi/anlys_gi;
687     double fnlys_gi  = 1/(Lys_water/gamman + Knlys_gi/gamman + D2lys_gi*Lys_water/gammad2 +
688     D2lys_gi*Kd2lys_gi/gammad2 + D1lys_gi*Lys_water/gammad1 + D1lys_gi*Kd1lys_gi/gammad1);
689     double fd1lys_gi =  fnlys_gi * D1lys_gi;
690     double fd2lys_gi =  fnlys_gi * D2lys_gi;
691     double Nd1lys_gi = valency1*Lys_E*Faraday/(R*Temp);
692     double Nd2lys_gi = valency2*Lys_E*Faraday/(R*Temp);
693     double Jgilys = fncyt_gi*Pn + fd2cyt_gi*Pd2*Nd2lys_gi/(exp(Nd2lys_gi)-1) +
694     fd1cyt_gi*Pd1*Nd1lys_gi/(exp(Nd1lys_gi)-1);
695     double Jlysgi = fnlys_gi*Pn + fd1lys_gi*Pd1*exp(Nd1lys_gi)*Nd1lys_gi/(exp(Nd1lys_gi)-1) +
696     fd2lys_gi*Pd2*exp(Nd2lys_gi)*Nd2lys_gi/(exp(Nd2lys_gi)-1);
697     // Spleen
698     double Kncyt_spleen  = 1.22 * Cyt_lipid * Kow;
699     double Kd1cyt_spleen = 1.22 * Cyt_lipid * Kowdiss;
700     double Kd2cyt_spleen = 1.22 * Cyt_lipid * Kowdiss2;
701     double ancyt_spleen  = 1/(1+ pow(10, pKa1-Cyt_pHinside) + pow(10, (pKa1-Cyt_pHinside)+(pKa2-
702     Cyt_pHinside)));
703     double ad2cyt_spleen = ancyt_spleen * pow(10, (pKa1-Cyt_pHinside)+(pKa2-Cyt_pHinside));
704     double ad1cyt_spleen = ancyt_spleen * pow(10, pKa1-Cyt_pHinside);
705     double D1cyt_spleen = ad1cyt_spleen/ancyt_spleen;
706     double D2cyt_spleen = ad2cyt_spleen/ancyt_spleen;
707     double fncyt_spleen  = 1/(Cyt_water/gamman + Kncyt_spleen/gamman +
708     D2cyt_spleen*Cyt_water/gammad2 + D2cyt_spleen*Kd2cyt_spleen/gammad2 +
709     D1cyt_spleen*Cyt_water/gammad1 + D1cyt_spleen*Kd1cyt_spleen/gammad1);
710     double fd1cyt_spleen =  fncyt_spleen * D1cyt_spleen;
711     double fd2cyt_spleen =  fncyt_spleen * D2cyt_spleen;
712     double Nd1cyt_spleen = valency1*Cyt_E*Faraday/(R*Temp);
713     double Nd2cyt_spleen = valency2*Cyt_E*Faraday/(R*Temp);
714     double Joutcytspleen = fnout*Pn + fd2out*Pd2*Nd2cyt_spleen/(exp(Nd2cyt_spleen)-1) +
715     fd1out*Pd1*Nd1cyt_spleen/(exp(Nd1cyt_spleen)-1);
716     double Jcytoutspleen = fncyt_spleen*Pn +
717     fd1cyt_spleen*Pd1*exp(Nd1cyt_spleen)*Nd1cyt_spleen/(exp(Nd1cyt_spleen)-1) +
718     fd2cyt_spleen*Pd2*exp(Nd2cyt_spleen)*Nd2cyt_spleen/(exp(Nd2cyt_spleen)-1);
719     double Knlys_spleen  = 1.22 * Lys_lipid * Kow;
720     double Kd1lys_spleen = 1.22 * Lys_lipid * Kowdiss;
721     double Kd2lys_spleen = 1.22 * Lys_lipid * Kowdiss2;
722     double anlys_spleen  = 1/(1+ pow(10, pKa1-Lys_pHinside_Aspleenlys) + pow(10, (pKa1-
723     Lys_pHinside_Aspleenlys)+(pKa2-Lys_pHinside_Aspleenlys)));
724     double ad2lys_spleen = anlys_spleen * pow(10, (pKa1-Lys_pHinside_Aspleenlys)+(pKa2-
725     Lys_pHinside_Aspleenlys));
726     double ad1lys_spleen = anlys_spleen * pow(10, pKa1-Lys_pHinside_Aspleenlys);
727     double D1lys_spleen = ad1lys_spleen/anlys_spleen;
728     double D2lys_spleen = ad2lys_spleen/anlys_spleen;
```

```
729 double fnlys_spleen  = 1/(Lys_water/gamman + Knlys_spleen/gamman +
730 D2lys_spleen*Lys_water/gammad2 + D2lys_spleen*Kd2lys_spleen/gammad2 +
731 D1lys_spleen*Lys_water/gammad1 + D1lys_spleen*Kd1lys_spleen/gammad1);
732 double fd1lys_spleen =  fnlys_spleen * D1lys_spleen;
733 double fd2lys_spleen =  fnlys_spleen * D2lys_spleen;
734 double Nd1lys_spleen = valency1*Lys_E*Faraday/(R*Temp);
735 double Nd2lys_spleen = valency2*Lys_E*Faraday/(R*Temp);
736 double Jspleenlys = fncyt_spleen*Pn + fd2cyt_spleen*Pd2*Nd2lys_spleen/(exp(Nd2lys_spleen)-1) +
737 fd1cyt_spleen*Pd1*Nd1lys_spleen/(exp(Nd1lys_spleen)-1);
738 double Jlysspleen = fnlys_spleen*Pn +
739 fd1lys_spleen*Pd1*exp(Nd1lys_spleen)*Nd1lys_spleen/(exp(Nd1lys_spleen)-1) +
740 fd2lys_spleen*Pd2*exp(Nd2lys_spleen)*Nd2lys_spleen/(exp(Nd2lys_spleen)-1);
741 // UA Lung
742 double Kncyt_UAlung  = 1.22 * Cyt_lipid * Kow;
743 double Kd1cyt_UAlung = 1.22 * Cyt_lipid * Kowdiss;
744 double Kd2cyt_UAlung = 1.22 * Cyt_lipid * Kowdiss2;
745 double ancyt_UAlung  = 1/(1+ pow(10, pKa1- Cyt_pHinside_AUATissue ) + pow(10, (pKa1-
746 Cyt_pHinside_AUATissue )+(pKa2- Cyt_pHinside_AUATissue )));
747 double ad2cyt_UAlung = ancyt_UAlung * pow(10, (pKa1- Cyt_pHinside_AUATissue )+(pKa2-
748 Cyt_pHinside_AUATissue ));
749 double ad1cyt_UAlung = ancyt_UAlung * pow(10, pKa1- Cyt_pHinside_AUATissue );
750 double D1cyt_UAlung = ad1cyt_UAlung/ancyt_UAlung;
751 double D2cyt_UAlung = ad2cyt_UAlung/ancyt_UAlung;
752 double fncyt_UAlung  = 1/(Cyt_water/gamman + Kncyt_UAlung/gamman +
753 D2cyt_UAlung*Cyt_water/gammad2 + D2cyt_UAlung*Kd2cyt_UAlung/gammad2 +
754 D1cyt_UAlung*Cyt_water/gammad1 + D1cyt_UAlung*Kd1cyt_UAlung/gammad1);
755 double fd1cyt_UAlung =  fncyt_UAlung * D1cyt_UAlung;
756 double fd2cyt_UAlung =  fncyt_UAlung * D2cyt_UAlung;
757 double Nd1cyt_UAlung = valency1*Cyt_E*Faraday/(R*Temp);
758 double Nd2cyt_UAlung = valency2*Cyt_E*Faraday/(R*Temp);
759 double JoutcytUAlung = fninter*Pn + fd2inter*Pd2*Nd2cyt_UAlung/(exp(Nd2cyt_UAlung)-1) +
760 fd1inter*Pd1*Nd1cyt_UAlung/(exp(Nd1cyt_UAlung)-1);
761 double JcytoutUAlung = fncyt_UAlung*Pn +
762 fd1cyt_UAlung*Pd1*exp(Nd1cyt_UAlung)*Nd1cyt_UAlung/(exp(Nd1cyt_UAlung)-1) +
763 fd2cyt_UAlung*Pd2*exp(Nd2cyt_UAlung)*Nd2cyt_UAlung/(exp(Nd2cyt_UAlung)-1);
764 double JmuccytUAlung = fnmucus*Pn + fd2mucus*Pd2*Nd2cyt_UAlung/(exp(Nd2cyt_UAlung)-1) +
765 fd1mucus*Pd1*Nd1cyt_UAlung/(exp(Nd1cyt_UAlung)-1);
766 double JcytmucUAlung = fncyt_UAlung*Pn +
767 fd1cyt_UAlung*Pd1*exp(Nd1cyt_UAlung)*Nd1cyt_UAlung/(exp(Nd1cyt_UAlung)-1) +
768 fd2cyt_UAlung*Pd2*exp(Nd2cyt_UAlung)*Nd2cyt_UAlung/(exp(Nd2cyt_UAlung)-1);
769 double Knlys_UAlung  = 1.22 * Lys_lipid * Kow;
770 double Kd1lys_UAlung = 1.22 * Lys_lipid * Kowdiss;
771 double Kd2lys_UAlung = 1.22 * Lys_lipid * Kowdiss2;
772 double anlys_UAlung  = 1/(1+ pow(10, pKa1-Lys_pHinside_AUATissuelys) + pow(10, (pKa1-
773 Lys_pHinside_AUATissuelys)+(pKa2-Lys_pHinside_AUATissuelys)));
774 double ad2lys_UAlung = anlys_UAlung * pow(10, (pKa1-Lys_pHinside_AUATissuelys)+(pKa2-
775 Lys_pHinside_AUATissuelys));
776 double ad1lys_UAlung = anlys_UAlung * pow(10, pKa1-Lys_pHinside_AUATissuelys);
777 double D1lys_UAlung = ad1lys_UAlung/anlys_UAlung;
778 double D2lys_UAlung = ad2lys_UAlung/anlys_UAlung;
```

```
779 double fnlys_UAlung  = 1/(Lys_water/gamman + Knlys_UAlung/gamman +
780 D2lys_UAlung*Lys_water/gammad2 + D2lys_UAlung*Kd2lys_UAlung/gammad2 +
781 D1lys_UAlung*Lys_water/gammad1 + D1lys_UAlung*Kd1lys_UAlung/gammad1);
782 double fd1lys_UAlung =  fnlys_UAlung * D1lys_UAlung;
783 double fd2lys_UAlung =  fnlys_UAlung * D2lys_UAlung;
784 double Nd1lys_UAlung = valency1*Lys_E*Faraday/(R*Temp);
785 double Nd2lys_UAlung = valency2*Lys_E*Faraday/(R*Temp);
786 double JUAlunglys = fncyt_UAlung*Pn + fd2cyt_UAlung*Pd2*Nd2lys_UAlung/(exp(Nd2lys_UAlung)-
787 1) + fd1cyt_UAlung*Pd1*Nd1lys_UAlung/(exp(Nd1lys_UAlung)-1);
788 double JlysUAlung = fnlys_UAlung*Pn + fd1lys_UAlung*Pd1*exp(Nd1lys_UAlung) * Nd1lys_UAlung
789 /(exp(Nd1lys_UAlung)-1) + fd2lys_UAlung*Pd2*exp(Nd2lys_UAlung)* Nd2lys_UAlung/ (exp
790 (Nd2lys_UAlung)-1);
791 // CA Lung
792 double Kncyt_CAlung  = 1.22 * Cyt_lipid * Kow;
793 double Kd1cyt_CAlung = 1.22 * Cyt_lipid * Kowdiss;
794 double Kd2cyt_CAlung = 1.22 * Cyt_lipid * Kowdiss2;
795 double ancyt_CAlung  = 1/(1+ pow(10, pKa1- Cyt_pHinside_ACATissue ) + pow(10, (pKa1-
796 Cyt_pHinside_ACATissue )+(pKa2- Cyt_pHinside_ACATissue )));
797 double ad2cyt_CAlung = ancyt_CAlung * pow(10, (pKa1- Cyt_pHinside_ACATissue )+(pKa2-
798 Cyt_pHinside_ACATissue ));
799 double ad1cyt_CAlung = ancyt_CAlung * pow(10, pKa1- Cyt_pHinside_ACATissue );
800 double D1cyt_CAlung = ad1cyt_CAlung/ancyt_CAlung;
801 double D2cyt_CAlung = ad2cyt_CAlung/ancyt_CAlung;
802 double fncyt_CAlung  = 1/(Cyt_water/gamman + Kncyt_CAlung/gamman +
803 D2cyt_CAlung*Cyt_water/gammad2 + D2cyt_CAlung*Kd2cyt_CAlung/gammad2 +
804 D1cyt_CAlung*Cyt_water/gammad1 + D1cyt_CAlung*Kd1cyt_CAlung/gammad1);
805 double fd1cyt_CAlung =  fncyt_CAlung * D1cyt_CAlung;
806 double fd2cyt_CAlung =  fncyt_CAlung * D2cyt_CAlung;
807 double Nd1cyt_CAlung = valency1*Cyt_E*Faraday/(R*Temp);
808 double Nd2cyt_CAlung = valency2*Cyt_E*Faraday/(R*Temp);
809 double JoutcytCAlung = fninter*Pn + fd2inter*Pd2*Nd2cyt_CAlung/(exp(Nd2cyt_CAlung)-1) +
810 fd1inter*Pd1*Nd1cyt_CAlung/(exp(Nd1cyt_CAlung)-1);
811 double JcytoutCAlung = fncyt_CAlung*Pn +
812 fd1cyt_CAlung*Pd1*exp(Nd1cyt_CAlung)*Nd1cyt_CAlung/(exp(Nd1cyt_CAlung)-1) +
813 fd2cyt_CAlung*Pd2*exp(Nd2cyt_CAlung)*Nd2cyt_CAlung/(exp(Nd2cyt_CAlung)-1);
814 double JmuccytCAlung = fnmucus*Pn + fd2mucus*Pd2*Nd2cyt_CAlung/(exp(Nd2cyt_CAlung)-1) +
815 fd1mucus*Pd1*Nd1cyt_CAlung/(exp(Nd1cyt_CAlung)-1);
816 double JcytmucCAlung = fncyt_CAlung*Pn +
817 fd1cyt_CAlung*Pd1*exp(Nd1cyt_CAlung)*Nd1cyt_CAlung/(exp(Nd1cyt_CAlung)-1) +
818 fd2cyt_CAlung*Pd2*exp(Nd2cyt_CAlung)*Nd2cyt_CAlung/(exp(Nd2cyt_CAlung)-1);
819 double Knlys_CAlung  = 1.22 * Lys_lipid * Kow;
820 double Kd1lys_CAlung = 1.22 * Lys_lipid * Kowdiss;
821 double Kd2lys_CAlung = 1.22 * Lys_lipid * Kowdiss2;
822 double anlys_CAlung  = 1/(1+ pow(10, pKa1-Lys_pHinside_ACATissuelys) + pow(10, (pKa1-
823 Lys_pHinside_ACATissuelys)+(pKa2-Lys_pHinside_ACATissuelys)));
824 double ad2lys_CAlung = anlys_CAlung * pow(10, (pKa1-Lys_pHinside_ACATissuelys)+(pKa2-
825 Lys_pHinside_ACATissuelys));
826 double ad1lys_CAlung = anlys_CAlung * pow(10, pKa1-Lys_pHinside_ACATissuelys);
827 double D1lys_CAlung = ad1lys_CAlung/anlys_CAlung;
828 double D2lys_CAlung = ad2lys_CAlung/anlys_CAlung;
```

```
829     double fnlys_CAlung  = 1/(Lys_water/gamman + Knlys_CAlung/gamman +
830     D2lys_CAlung*Lys_water/gammad2 + D2lys_CAlung*Kd2lys_CAlung/gammad2 +
831     D1lys_CAlung*Lys_water/gammad1 + D1lys_CAlung*Kd1lys_CAlung/gammad1);
832     double fd1lys_CAlung =  fnlys_CAlung * D1lys_CAlung;
833     double fd2lys_CAlung =  fnlys_CAlung * D2lys_CAlung;
834     double Nd1lys_CAlung = valency1*Lys_E*Faraday/(R*Temp);
835     double Nd2lys_CAlung = valency2*Lys_E*Faraday/(R*Temp);
836     double JCAlunglys = fncyt_CAlung*Pn + fd2cyt_CAlung*Pd2*Nd2lys_CAlung/(exp(Nd2lys_CAlung)-
837     1) + fd1cyt_CAlung*Pd1*Nd1lys_CAlung/(exp(Nd1lys_CAlung)-1);
838     double JlysCAlung = fnlys_CAlung*Pn +
839     fd1lys_CAlung*Pd1*exp(Nd1lys_CAlung)*Nd1lys_CAlung/(exp(Nd1lys_CAlung)-1) +
840     fd2lys_CAlung*Pd2*exp(Nd2lys_CAlung)*Nd2lys_CAlung/(exp(Nd2lys_CAlung)-1);
841     // TA Lung
842     double Kncyt_TAlung  = 1.22 * Cyt_lipid * Kow;
843     double Kd1cyt_TAlung = 1.22 * Cyt_lipid * Kowdiss;
844     double Kd2cyt_TAlung = 1.22 * Cyt_lipid * Kowdiss2;
845     double ancyt_TAlung  = 1/(1+ pow(10, pKa1- Cyt_pHinside_ATATissue ) + pow(10, (pKa1-
846     Cyt_pHinside_ATATissue )+(pKa2- Cyt_pHinside_ATATissue )));
847     double ad2cyt_TAlung = ancyt_TAlung * pow(10, (pKa1- Cyt_pHinside_ATATissue )+(pKa2-
848     Cyt_pHinside_ATATissue ));
849     double ad1cyt_TAlung = ancyt_TAlung * pow(10, pKa1- Cyt_pHinside_ATATissue );
850     double D1cyt_TAlung = ad1cyt_TAlung/ancyt_TAlung;
851     double D2cyt_TAlung = ad2cyt_TAlung/ancyt_TAlung;
852     double fncyt_TAlung  = 1/(Cyt_water/gamman + Kncyt_TAlung/gamman +
853     D2cyt_TAlung*Cyt_water/gammad2 + D2cyt_TAlung*Kd2cyt_TAlung/gammad2 +
854     D1cyt_TAlung*Cyt_water/gammad1 + D1cyt_TAlung*Kd1cyt_TAlung/gammad1);
855     double fd1cyt_TAlung =  fncyt_TAlung * D1cyt_TAlung;
856     double fd2cyt_TAlung =  fncyt_TAlung * D2cyt_TAlung;
857     double Nd1cyt_TAlung = valency1*Cyt_E*Faraday/(R*Temp);
858     double Nd2cyt_TAlung = valency2*Cyt_E*Faraday/(R*Temp);
859     double JoutcytTAlung = fninter*Pn + fd2inter*Pd2*Nd2cyt_TAlung/(exp(Nd2cyt_TAlung)-1) +
860     fd1inter*Pd1*Nd1cyt_TAlung/(exp(Nd1cyt_TAlung)-1);
861     double JcytoutTAlung = fncyt_TAlung*Pn +
862     fd1cyt_TAlung*Pd1*exp(Nd1cyt_TAlung)*Nd1cyt_TAlung/(exp(Nd1cyt_TAlung)-1) +
863     fd2cyt_TAlung*Pd2*exp(Nd2cyt_TAlung)*Nd2cyt_TAlung/(exp(Nd2cyt_TAlung)-1);
864     double JmuccytTAlung = fnmucus*Pn + fd2mucus*Pd2*Nd2cyt_TAlung/(exp(Nd2cyt_TAlung)-1) +
865     fd1mucus*Pd1*Nd1cyt_TAlung/(exp(Nd1cyt_TAlung)-1);
866     double JcytmucTAlung = fncyt_TAlung*Pn +
867     fd1cyt_TAlung*Pd1*exp(Nd1cyt_TAlung)*Nd1cyt_TAlung/(exp(Nd1cyt_TAlung)-1) +
868     fd2cyt_TAlung*Pd2*exp(Nd2cyt_TAlung)*Nd2cyt_TAlung/(exp(Nd2cyt_TAlung)-1);
869     double Knlys_TAlung  = 1.22 * Lys_lipid * Kow;
870     double Kd1lys_TAlung = 1.22 * Lys_lipid * Kowdiss;
871     double Kd2lys_TAlung = 1.22 * Lys_lipid * Kowdiss2;
872     double anlys_TAlung  = 1/(1+ pow(10, pKa1-Lys_pHinside_ATATissuelys) + pow(10, (pKa1-
873     Lys_pHinside_ATATissuelys)+(pKa2-Lys_pHinside_ATATissuelys)));
874     double ad2lys_TAlung = anlys_TAlung * pow(10, (pKa1-Lys_pHinside_ATATissuelys)+(pKa2-
875     Lys_pHinside_ATATissuelys));
876     double ad1lys_TAlung = anlys_TAlung * pow(10, pKa1-Lys_pHinside_ATATissuelys);
877     double D1lys_TAlung = ad1lys_TAlung/anlys_TAlung;
878     double D2lys_TAlung = ad2lys_TAlung/anlys_TAlung;
```

```
879 double fnlys_TAlung  = 1/(Lys_water/gamman + Knlys_TAlung/gamman +
880 D2lys_TAlung*Lys_water/gammad2 + D2lys_TAlung*Kd2lys_TAlung/gammad2 +
881 D1lys_TAlung*Lys_water/gammad1 + D1lys_TAlung*Kd1lys_TAlung/gammad1);
882 double fd1lys_TAlung =  fnlys_TAlung * D1lys_TAlung;
883 double fd2lys_TAlung =  fnlys_TAlung * D2lys_TAlung;
884 double Nd1lys_TAlung = valency1*Lys_E*Faraday/(R*Temp);
885 double Nd2lys_TAlung = valency2*Lys_E*Faraday/(R*Temp);
886 double JTAlunglys = fncyt_TAlung*Pn + fd2cyt_TAlung*Pd2*Nd2lys_TAlung/(exp(Nd2lys_TAlung)-1)
887 + fd1cyt_TAlung*Pd1*Nd1lys_TAlung/(exp(Nd1lys_TAlung)-1);
888 double JlysTAlung = fnlys_TAlung*Pn +
889 fd1lys_TAlung*Pd1*exp(Nd1lys_TAlung)*Nd1lys_TAlung/(exp(Nd1lys_TAlung)-1) +
890 fd2lys_TAlung*Pd2*exp(Nd2lys_TAlung)*Nd2lys_TAlung/(exp(Nd2lys_TAlung)-1);
891 // PA Lung
892 double Kncyt_PAlung  = 1.22 * Cyt_lipid * Kow;
893 double Kd1cyt_PAlung = 1.22 * Cyt_lipid * Kowdiss;
894 double Kd2cyt_PAlung = 1.22 * Cyt_lipid * Kowdiss2;
895 double ancyt_PAlung  = 1/(1+ pow(10, pKa1- Cyt_pHinside_APATissue ) + pow(10, (pKa1-
896 Cyt_pHinside_APATissue )+(pKa2- Cyt_pHinside_APATissue )));
897 double ad2cyt_PAlung = ancyt_PAlung * pow(10, (pKa1- Cyt_pHinside_APATissue )+(pKa2-
898 Cyt_pHinside_APATissue ));
899 double ad1cyt_PAlung = ancyt_PAlung * pow(10, pKa1- Cyt_pHinside_APATissue );
900 double D1cyt_PAlung = ad1cyt_PAlung/ancyt_PAlung;
901 double D2cyt_PAlung = ad2cyt_PAlung/ancyt_PAlung;
902 double fncyt_PAlung  = 1/(Cyt_water/gamman + Kncyt_PAlung/gamman +
903 D2cyt_PAlung*Cyt_water/gammad2 + D2cyt_PAlung*Kd2cyt_PAlung/gammad2 +
904 D1cyt_PAlung*Cyt_water/gammad1 + D1cyt_PAlung*Kd1cyt_PAlung/gammad1);
905 double fd1cyt_PAlung =  fncyt_PAlung * D1cyt_PAlung;
906 double fd2cyt_PAlung =  fncyt_PAlung * D2cyt_PAlung;
907 double Nd1cyt_PAlung = valency1*Cyt_E*Faraday/(R*Temp);
908 double Nd2cyt_PAlung = valency2*Cyt_E*Faraday/(R*Temp);
909 double JoutcytPAlung = fninter*Pn + fd2inter*Pd2*Nd2cyt_PAlung/(exp(Nd2cyt_PAlung)-1) +
910 fd1inter*Pd1*Nd1cyt_PAlung/(exp(Nd1cyt_PAlung)-1);
911 double JcytoutPAlung = fncyt_PAlung*Pn +
912 fd1cyt_PAlung*Pd1*exp(Nd1cyt_PAlung)*Nd1cyt_PAlung/(exp(Nd1cyt_PAlung)-1) +
913 fd2cyt_PAlung*Pd2*exp(Nd2cyt_PAlung)*Nd2cyt_PAlung/(exp(Nd2cyt_PAlung)-1);
914 double JmuccytPAlung = fnmucus*Pn + fd2mucus*Pd2*Nd2cyt_PAlung/(exp(Nd2cyt_PAlung)-1) +
915 fd1mucus*Pd1*Nd1cyt_PAlung/(exp(Nd1cyt_PAlung)-1);
916 double JcytmucPAlung = fncyt_PAlung*Pn +
917 fd1cyt_PAlung*Pd1*exp(Nd1cyt_PAlung)*Nd1cyt_PAlung/(exp(Nd1cyt_PAlung)-1) +
918 fd2cyt_PAlung*Pd2*exp(Nd2cyt_PAlung)*Nd2cyt_PAlung/(exp(Nd2cyt_PAlung)-1);
919 double Knlys_PAlung  = 1.22 * Lys_lipid * Kow;
920 double Kd1lys_PAlung = 1.22 * Lys_lipid * Kowdiss;
921 double Kd2lys_PAlung = 1.22 * Lys_lipid * Kowdiss2;
922 double anlys_PAlung  = 1/(1+ pow(10, pKa1-Lys_pHinside_APATissuelys) + pow(10, (pKa1-
923 Lys_pHinside_APATissuelys)+(pKa2-Lys_pHinside_APATissuelys)));
924 double ad2lys_PAlung = anlys_PAlung * pow(10, (pKa1-Lys_pHinside_APATissuelys)+(pKa2-
925 Lys_pHinside_APATissuelys));
926 double ad1lys_PAlung = anlys_PAlung * pow(10, pKa1-Lys_pHinside_APATissuelys);
927 double D1lys_PAlung = ad1lys_PAlung/anlys_PAlung;
928 double D2lys_PAlung = ad2lys_PAlung/anlys_PAlung;
```

```
929 double fnlys_PAlung  = 1/(Lys_water/gamman + Knlys_PAlung/gamman +
930 D2lys_PAlung*Lys_water/gammad2 + D2lys_PAlung*Kd2lys_PAlung/gammad2 +
931 D1lys_PAlung*Lys_water/gammad1 + D1lys_PAlung*Kd1lys_PAlung/gammad1);
932 double fd1lys_PAlung =  fnlys_PAlung * D1lys_PAlung;
933 double fd2lys_PAlung =  fnlys_PAlung * D2lys_PAlung;
934 double Nd1lys_PAlung = valency1*Lys_E*Faraday/(R*Temp);
935 double Nd2lys_PAlung = valency2*Lys_E*Faraday/(R*Temp);
936 double JPAlunglys = fncyt_PAlung*Pn + fd2cyt_PAlung*Pd2*Nd2lys_PAlung/(exp(Nd2lys_PAlung)-1)
937 + fd1cyt_PAlung*Pd1*Nd1lys_PAlung/(exp(Nd1lys_PAlung)-1);
938 double JlysPAlung = fnlys_PAlung*Pn +
939 fd1lys_PAlung*Pd1*exp(Nd1lys_PAlung)*Nd1lys_PAlung/(exp(Nd1lys_PAlung)-1) +
940 fd2lys_PAlung*Pd2*exp(Nd2lys_PAlung)*Nd2lys_PAlung/(exp(Nd2lys_PAlung)-1);
941 // muscle
942 double Kncyt_muscle  = 1.22 * (Cyt_lipid) * Kow;
943 double Kd1cyt_muscle = 1.22 * (Cyt_lipid) * Kowdiss;
944 double Kd2cyt_muscle = 1.22 * (Cyt_lipid) * Kowdiss2;
945 double ancyt_muscle  = 1/(1+ pow(10, pKa1-Cyt_pHinside) + pow(10, (pKa1-Cyt_pHinside)+(pKa2-
946 Cyt_pHinside)));
947 double ad2cyt_muscle = ancyt_muscle * pow(10, (pKa1-Cyt_pHinside)+(pKa2-Cyt_pHinside));
948 double ad1cyt_muscle = ancyt_muscle * pow(10, pKa1-Cyt_pHinside);
949 double D1cyt_muscle = ad1cyt_muscle/ancyt_muscle;
950 double D2cyt_muscle = ad2cyt_muscle/ancyt_muscle;
951 double fncyt_muscle  = 1/((Cyt_water)/gamman + Kncyt_muscle/gamman +
952 D2cyt_muscle*(Cyt_water)/gammad2 + D2cyt_muscle*Kd2cyt_muscle/gammad2 +
953 D1cyt_muscle*(Cyt_water)/gammad1 + D1cyt_muscle*Kd1cyt_muscle/gammad1);
954 double fd1cyt_muscle =  fncyt_muscle * D1cyt_muscle;
955 double fd2cyt_muscle =  fncyt_muscle * D2cyt_muscle;
956 double Nd1cyt_muscle = valency1*Cyt_E *Faraday/(R*Temp);
957 double Nd2cyt_muscle = valency2*Cyt_E*Faraday/(R*Temp);
958 double Joutcytmuscle = fnout*Pn + fd2out*Pd2*Nd2cyt_muscle/(exp(Nd2cyt_muscle)-1) +
959 fd1out*Pd1*Nd1cyt_muscle/(exp(Nd1cyt_muscle)-1);
960 double Jcytoutmuscle = fncyt_muscle*Pn +
961 fd1cyt_muscle*Pd1*exp(Nd1cyt_muscle)*Nd1cyt_muscle/(exp(Nd1cyt_muscle)-1) +
962 fd2cyt_muscle*Pd2*exp(Nd2cyt_muscle)*Nd2cyt_muscle/(exp(Nd2cyt_muscle)-1);
963 double Knlys_muscle  = 1.22 * Lys_lipid * Kow;
964 double Kd1lys_muscle = 1.22 * Lys_lipid * Kowdiss;
965 double Kd2lys_muscle = 1.22 * Lys_lipid * Kowdiss2;
966 double anlys_muscle = 1/(1+ pow(10, pKa1-Lys_pHinside_Amusclelys) + pow(10, (pKa1-
967 Lys_pHinside_Amusclelys)+(pKa2-Lys_pHinside_Amusclelys)));
968 double ad2lys_muscle = anlys_muscle * pow(10, (pKa1-Lys_pHinside_Amusclelys)+(pKa2-
969 Lys_pHinside_Amusclelys));
970 double ad1lys_muscle = anlys_muscle * pow(10, pKa1-Lys_pHinside_Amusclelys);
971 double D1lys_muscle = ad1lys_muscle/anlys_muscle;
972 double D2lys_muscle = ad2lys_muscle/anlys_muscle;
973 double fnlys_muscle = 1/(Lys_water/gamman + Knlys_muscle/gamman +
974 D2lys_muscle*Lys_water/gammad2 + D2lys_muscle*Kd2lys_muscle/gammad2 +
975 D1lys_muscle*Lys_water/gammad1 + D1lys_muscle*Kd1lys_muscle/gammad1);
976 double fd1lys_muscle = fnlys_muscle * D1lys_muscle;
977 double fd2lys_muscle = fnlys_muscle * D2lys_muscle;
978 double Nd1lys_muscle = valency1*Lys_E*Faraday/(R*Temp);
979 double Nd2lys_muscle = valency2*Lys_E*Faraday/(R*Temp);
```

```
980   double Jmusclelys = fncyt_muscle*Pn + fd2cyt_muscle*Pd2*Nd2cyt_muscle/(exp(Nd2cyt_muscle)-1) +
981   fd1cyt_muscle*Pd1*Nd1cyt_muscle/(exp(Nd1cyt_muscle)-1);
982   double Jlysmuscle = fnlys_muscle*Pn +
983   fd1lys_muscle*Pd1*exp(Nd1lys_muscle)*Nd1lys_muscle/(exp(Nd1lys_muscle)-1) +
984   fd2lys_muscle*Pd2*exp(Nd2lys_muscle)*Nd2lys_muscle/(exp(Nd2lys_muscle)-1);
985   // skin
986   double Kncyt_skin  = 1.22 * Cyt_lipid * Kow;
987   double Kd1cyt_skin = 1.22 * Cyt_lipid * Kowdiss;
988   double Kd2cyt_skin = 1.22 * Cyt_lipid * Kowdiss2;
989   double ancyt_skin  = 1/(1+ pow(10, pKa1-Cyt_pHinside) + pow(10, (pKa1-Cyt_pHinside)+(pKa2-
990   Cyt_pHinside)));
991   double ad2cyt_skin = ancyt_skin * pow(10, (pKa1-Cyt_pHinside)+(pKa2-Cyt_pHinside));
992   double ad1cyt_skin = ancyt_skin * pow(10, pKa1-Cyt_pHinside);
993   double D1cyt_skin = ad1cyt_skin/ancyt_skin;
994   double D2cyt_skin = ad2cyt_skin/ancyt_skin;
995   double fncyt_skin  = 1/(Cyt_water/gamman + Kncyt_skin/gamman + D2cyt_skin*Cyt_water/gammad2 +
996   D2cyt_skin*Kd2cyt_skin/gammad2 + D1cyt_skin*Cyt_water/gammad1 +
997   D1cyt_skin*Kd1cyt_skin/gammad1);
998   double fd1cyt_skin =  fncyt_skin * D1cyt_skin;
999   double fd2cyt_skin =  fncyt_skin * D2cyt_skin;
1000  double Nd1cyt_skin = valency1*Cyt_E*Faraday/(R*Temp);
1001  double Nd2cyt_skin = valency2*Cyt_E*Faraday/(R*Temp);
1002  double Joutcytskin = fnout*Pn + fd2out*Pd2*Nd2cyt_skin/(exp(Nd2cyt_skin)-1) +
1003  fd1out*Pd1*Nd1cyt_skin/(exp(Nd1cyt_skin)-1);
1004  double Jcytoutskin = fncyt_skin*Pn +
1005  fd1cyt_skin*Pd1*exp(Nd1cyt_skin)*Nd1cyt_skin/(exp(Nd1cyt_skin)-1) +
1006  fd2cyt_skin*Pd2*exp(Nd2cyt_skin)*Nd2cyt_skin/(exp(Nd2cyt_skin)-1);
1007  double Knlys_skin  = 1.22 * Lys_lipid * Kow;
1008  double Kd1lys_skin = 1.22 * Lys_lipid * Kowdiss;
1009  double Kd2lys_skin = 1.22 * Lys_lipid * Kowdiss2;
1010  double anlys_skin  = 1/(1+ pow(10, pKa1-Lys_pHinside_Askinlys) + pow(10, (pKa1-
1011  Lys_pHinside_Askinlys)+(pKa2-Lys_pHinside_Askinlys)));
1012  double ad2lys_skin = anlys_skin * pow(10, (pKa1-Lys_pHinside_Askinlys)+(pKa2-
1013  Lys_pHinside_Askinlys));
1014  double ad1lys_skin = anlys_skin * pow(10, pKa1-Lys_pHinside_Askinlys);
1015  double D1lys_skin = ad1lys_skin/anlys_skin;
1016  double D2lys_skin = ad2lys_skin/anlys_skin;
1017  double fnlys_skin  = 1/(Lys_water/gamman + Knlys_skin/gamman + D2lys_skin*Lys_water/gammad2 +
1018  D2lys_skin*Kd2lys_skin/gammad2 + D1lys_skin*Lys_water/gammad1 +
1019  D1lys_skin*Kd1lys_skin/gammad1);
1020  double fd1lys_skin =  fnlys_skin * D1lys_skin;
1021  double fd2lys_skin =  fnlys_skin * D2lys_skin;
1022  double Nd1lys_skin = valency1*Lys_E*Faraday/(R*Temp);
1023  double Nd2lys_skin = valency2*Lys_E*Faraday/(R*Temp);
1024  double Jskinlys = fncyt_skin*Pn + fd2cyt_skin*Pd2*Nd2lys_skin/(exp(Nd2lys_skin)-1) +
1025  fd1cyt_skin*Pd1*Nd1lys_skin/(exp(Nd1lys_skin)-1);
1026  double Jlysskin = fnlys_skin*Pn + fd1lys_skin*Pd1*exp(Nd1lys_skin)*Nd1lys_skin/(exp(Nd1lys_skin)-
1027  1) + fd2lys_skin*Pd2*exp(Nd2lys_skin)*Nd2lys_skin/(exp(Nd2lys_skin)-1);
1028  // slow
1029  double Kncyt_slow  = 1.22 * Cyt_lipid * Kow;
1030  double Kd1cyt_slow = 1.22 * Cyt_lipid * Kowdiss;
```

```
1031    double Kd2cyt_slow = 1.22 * Cyt_lipid * Kowdiss2;
1032    double ancyt_slow  = 1/(1+ pow(10, pKa1-Cyt_pHinside) + pow(10, (pKa1-Cyt_pHinside)+(pKa2-
1033    Cyt_pHinside)));
1034    double ad2cyt_slow = ancyt_slow * pow(10, (pKa1-Cyt_pHinside)+(pKa2-Cyt_pHinside));
1035    double ad1cyt_slow = ancyt_slow * pow(10, pKa1-Cyt_pHinside);
1036    double D1cyt_slow = ad1cyt_slow/ancyt_slow;
1037    double D2cyt_slow = ad2cyt_slow/ancyt_slow;
1038    double fncyt_slow  = 1/(Cyt_water/gamman + Kncyt_slow/gamman + D2cyt_slow*Cyt_water/gammad2
1039    + D2cyt_slow*Kd2cyt_slow/gammad2 + D1cyt_slow*Cyt_water/gammad1 +
1040    D1cyt_slow*Kd1cyt_slow/gammad1);
1041    double fd1cyt_slow =  fncyt_slow * D1cyt_slow;
1042    double fd2cyt_slow =  fncyt_slow * D2cyt_slow;
1043    double Nd1cyt_slow = valency1*Cyt_E*Faraday/(R*Temp);
1044    double Nd2cyt_slow = valency2*Cyt_E*Faraday/(R*Temp);
1045    double Joutcytslow = fnout*Pn + fd2out*Pd2*Nd2cyt_slow/(exp(Nd2cyt_slow)-1) +
1046    fd1out*Pd1*Nd1cyt_slow/(exp(Nd1cyt_slow)-1);
1047    double Jcytoutslow = fncyt_slow*Pn +
1048    fd1cyt_slow*Pd1*exp(Nd1cyt_slow)*Nd1cyt_slow/(exp(Nd1cyt_slow)-1) +
1049    fd2cyt_slow*Pd2*exp(Nd2cyt_slow)*Nd2cyt_slow/(exp(Nd2cyt_slow)-1);
1050    double Knlys_slow  = 1.22 * Lys_lipid * Kow;
1051    double Kd1lys_slow = 1.22 * Lys_lipid * Kowdiss;
1052    double Kd2lys_slow = 1.22 * Lys_lipid * Kowdiss2;
1053    double anlys_slow  = 1/(1+ pow(10, pKa1-Lys_pHinside_Aslowlys) + pow(10, (pKa1-
1054    Lys_pHinside_Aslowlys)+(pKa2-Lys_pHinside_Aslowlys)));
1055    double ad2lys_slow = anlys_slow * pow(10, (pKa1-Lys_pHinside_Aslowlys)+(pKa2-
1056    Lys_pHinside_Aslowlys));
1057    double ad1lys_slow = anlys_slow * pow(10, pKa1-Lys_pHinside_Aslowlys);
1058    double D1lys_slow = ad1lys_slow/anlys_slow;
1059    double D2lys_slow = ad2lys_slow/anlys_slow;
1060    double fnlys_slow  = 1/(Lys_water/gamman + Knlys_slow/gamman + D2lys_slow*Lys_water/gammad2
1061    + D2lys_slow*Kd2lys_slow/gammad2 + D1lys_slow*Lys_water/gammad1 +
1062    D1lys_slow*Kd1lys_slow/gammad1);
1063    double fd1lys_slow =  fnlys_slow * D1lys_slow;
1064    double fd2lys_slow =  fnlys_slow * D2lys_slow;
1065    double Nd1lys_slow = valency1*Lys_E*Faraday/(R*Temp);
1066    double Nd2lys_slow = valency2*Lys_E*Faraday/(R*Temp);
1067    double Jslowlys = fncyt_slow*Pn + fd2cyt_slow*Pd2*Nd2lys_slow/(exp(Nd2lys_slow)-1) +
1068    fd1cyt_slow*Pd1*Nd1lys_slow/(exp(Nd1lys_slow)-1);
1069    double Jlysslow = fnlys_slow*Pn +
1070    fd1lys_slow*Pd1*exp(Nd1lys_slow)*Nd1lys_slow/(exp(Nd1lys_slow)-1) +
1071    fd2lys_slow*Pd2*exp(Nd2lys_slow)*Nd2lys_slow/(exp(Nd2lys_slow)-1);
1072    // other
1073    double Kncyt_other  = 1.22 * Cyt_lipid * Kow;
1074    double Kd1cyt_other = 1.22 * Cyt_lipid * Kowdiss;
1075    double Kd2cyt_other = 1.22 * Cyt_lipid * Kowdiss2;
1076    double ancyt_other  = 1/(1+ pow(10, pKa1-Cyt_pHinside) + pow(10, (pKa1-Cyt_pHinside)+(pKa2-
1077    Cyt_pHinside)));
1078    double ad2cyt_other = ancyt_other * pow(10, (pKa1-Cyt_pHinside)+(pKa2-Cyt_pHinside));
1079    double ad1cyt_other = ancyt_other * pow(10, pKa1-Cyt_pHinside);
1080    double D1cyt_other = ad1cyt_other/ancyt_other;
1081    double D2cyt_other = ad2cyt_other/ancyt_other;
```

```
1082    double fncyt_other  = 1/(Cyt_water/gamman + Kncyt_other/gamman + D2cyt_other*Cyt_water/gammad2
1083    + D2cyt_other*Kd2cyt_other/gammad2 + D1cyt_other*Cyt_water/gammad1 +
1084    D1cyt_other*Kd1cyt_other/gammad1);
1085    double fd1cyt_other =  fncyt_other * D1cyt_other;
1086    double fd2cyt_other =  fncyt_other * D2cyt_other;
1087    double Nd1cyt_other = valency1*Cyt_E*Faraday/(R*Temp);
1088    double Nd2cyt_other = valency2*Cyt_E*Faraday/(R*Temp);
1089    double Joutcytother = fnout*Pn + fd2out*Pd2*Nd2cyt_other/(exp(Nd2cyt_other)-1) +
1090    fd1out*Pd1*Nd1cyt_other/(exp(Nd1cyt_other)-1);
1091    double Jcytoutother = fncyt_other*Pn +
1092    fd1cyt_other*Pd1*exp(Nd1cyt_other)*Nd1cyt_other/(exp(Nd1cyt_other)-1) +
1093    fd2cyt_other*Pd2*exp(Nd2cyt_other)*Nd2cyt_other/(exp(Nd2cyt_other)-1);
1094    double Knlys_other  = 1.22 * Lys_lipid * Kow;
1095    double Kd1lys_other = 1.22 * Lys_lipid * Kowdiss;
1096    double Kd2lys_other = 1.22 * Lys_lipid * Kowdiss2;
1097    double anlys_other  = 1/(1+ pow(10, pKa1-Lys_pHinside_Aotherlys) + pow(10, (pKa1-
1098    Lys_pHinside_Aotherlys)+(pKa2-Lys_pHinside_Aotherlys)));
1099    double ad2lys_other = anlys_other * pow(10, (pKa1-Lys_pHinside_Aotherlys)+(pKa2-
1100    Lys_pHinside_Aotherlys));
1101    double ad1lys_other = anlys_other * pow(10, pKa1-Lys_pHinside_Aotherlys);
1102    double D1lys_other = ad1lys_other/anlys_other;
1103    double D2lys_other = ad2lys_other/anlys_other;
1104    double fnlys_other  = 1/(Lys_water/gamman + Knlys_other/gamman + D2lys_other*Lys_water/gammad2
1105    + D2lys_other*Kd2lys_other/gammad2 + D1lys_other*Lys_water/gammad1 +
1106    D1lys_other*Kd1lys_other/gammad1);
1107    double fd1lys_other =  fnlys_other * D1lys_other;
1108    double fd2lys_other =  fnlys_other * D2lys_other;
1109    double Nd1lys_other = valency1*Lys_E*Faraday/(R*Temp);
1110    double Nd2lys_other = valency2*Lys_E*Faraday/(R*Temp);
1111    double Jotherlys = fncyt_other*Pn + fd2cyt_other*Pd2*Nd2lys_other/(exp(Nd2lys_other)-1) +
1112    fd1cyt_other*Pd1*Nd1lys_other/(exp(Nd1lys_other)-1);
1113    double Jlysother = fnlys_other*Pn +
1114    fd1lys_other*Pd1*exp(Nd1lys_other)*Nd1lys_other/(exp(Nd1lys_other)-1) +
1115    fd2lys_other*Pd2*exp(Nd2lys_other)*Nd2lys_other/(exp(Nd2lys_other)-1);
1116
1117    // UA
1118    dxdt_AUAmucus = 1/VUA_mucus * (-DC/ TUA_mucus *SA_UA*(AUAmucus - AUAasl)) - KmucUA *
1119    AUAmucus + 1/VUA_mucus * (KmucCA * ACAmucus * VCA_mucus);
1120    dxdt_AUAasl = 1/VUA_asl * (DC/ TUA_mucus *SA_UA*(AUAmucus - AUAasl) +
1121    SA_UA*(JcytmucUAlung*AUATissue - JmuccytUAlung*AUAasl) + SA_UA* Vmaxpgp
1122    *Fu*AUATissue*fncyt_UAlung/(Kmpgp + Fu*AUATissue*fncyt_UAlung) + SA_UA*Vmaxpgp1*
1123    Fu*AUATissue*fncyt_UAlung/(Kmpgp1 + Fu*AUATissue*fncyt_UAlung)) - KmucUA * AUAasl +
1124    1/VUA_asl * (KmucCA * ACAasl * VCA_asl);
1125    dxdt_AUATissue = 1/(VUAtiss-VUAtiss_EW-VUAtisslys-VUAtiss_Vas) * (-
1126    SA_UA*(JcytmucUAlung*AUATissue - JmuccytUAlung*AUAasl) + SA_UA *(JoutcytUAlung*
1127    Fu*AUATissueinter - JcytoutUAlung*AUATissue) - SAlysUAtiss * (JUAlunglys * AUATissue -
1128    JlysUAlung * AUATissuelys) - SA_UA*Vmaxpgp*Fu*AUATissue*fncyt_UAlung/(Kmpgp +
1129    Fu*AUATissue*fncyt_UAlung) - SA_UA*Vmaxpgp1 *Fu*AUATissue*fncyt_UAlung/(Kmpgp1 +
1130    Fu*AUATissue*fncyt_UAlung));
1131    dxdt_AUATissueinter = 1/VUAtiss_EW * (Qua*(AUATissuevascular/BP - AUATissueinter) -
1132    SA_UA*(JoutcytUAlung*Fu*AUATissueinter - JcytoutUAlung*AUATissue));
```

```
dxdt_AUATissuelys = 1/(VUAtisslys) * SAlysUAtiss * (JUAlunglys * AUATissue - JlysUAlung *
AUATissuelys);
dxdt_AUATissuevascular = 1/VUAtiss_Vas *(Qua*(Aarterial - AUATissuevascular) -Qua
*(AUATissuevascular/BP - AUATissueinter));

// CA
dxdt_ACAmucus = 1/VCA_mucus * (-DC/ TCA_mucus *SA_CA*(ACAmucus - ACAasl)) - KmucCA *
ACAmucus + 1/VCA_mucus * (KmucTA * ATAmucus * VTA_mucus);
dxdt_ACAasl = 1/VCA_asl * (DC/ TCA_mucus *SA_CA*(ACAmucus - ACAasl) +
SA_CA*(JcytmucCAlung*ACATissue - JmuccytCAlung*ACAasl) + SA_CA*Vmaxpgp
*Fu*ACATissue*fncyt_CAlung/(Kmpgp + Fu*ACATissue*fncyt_CAlung) + SA_CA*Vmaxpgp1
*Fu*ACATissue*fncyt_CAlung/(Kmpgp1 + Fu*ACATissue*fncyt_CAlung)) - KmucCA * ACAasl +
1/VCA_asl * (KmucTA * ATAasl * VTA_asl);
dxdt_ACATissue = 1/(VCAtiss-VCAtiss_EW-VCAtisslys-VCAtiss_Vas) * (-
SA_CA*(JcytmucCAlung*ACATissue - JmuccytCAlung*ACAasl) + SA_CA*(JoutcytCAlung*
Fu*ACATissueinter - JcytoutCAlung*ACATissue) - SAlysCAtiss*(JCAlunglys * ACATissue -
JlysCAlung * ACATissuelys) - SA_CA*Vmaxpgp* Fu*ACATissue*fncyt_CAlung/(Kmpgp +
Fu*ACATissue*fncyt_CAlung) - SA_CA*Vmaxpgp1*Fu*ACATissue*fncyt_CAlung/(Kmpgp1 +
Fu*ACATissue*fncyt_CAlung));
dxdt_ACATissueinter = 1/VCAtiss_EW * (Qca*(ACATissuevascular/BP - ACATissueinter) -
SA_CA*(JoutcytCAlung* Fu*ACATissueinter - JcytoutCAlung*ACATissue));
dxdt_ACATissuelys = 1/(VCAtisslys) * SAlysCAtiss * (JCAlunglys * ACATissue - JlysCAlung *
ACATissuelys);
dxdt_ACATissuevascular = 1/VCAtiss_Vas *(Qca * (Aarterial - ACATissuevascular) -Qca
*(ACATissuevascular/BP - ACATissueinter));

// TA
dxdt_ATAmucus = 1/VTA_mucus * (-DC/ TTA_mucus *SA_TA*(ATAmucus-ATAasl))-
KmucTA*ATAmucus;
dxdt_ATAasl = 1/VTA_asl * (DC/ TTA_mucus *SA_TA*(ATAmucus-ATAasl) +
SA_TA*(JcytmucTAlung*ATATissue - JmuccytTAlung*ATAasl) +
SA_TA*Vmaxpgp*Fu*ATATissue*fncyt_TAlung/(Kmpgp + Fu*ATATissue*fncyt_TAlung) +
SA_TA*Vmaxpgp1*Fu*ATATissue*fncyt_TAlung/(Kmpgp1 + Fu*ATATissue*fncyt_TAlung)) -
KmucTA * ATAasl;
dxdt_ATATissue = 1/(VTAtiss-VTAtiss_EW-VTAtisslys-VTAtiss_Vas) * (-
SA_TA*(JcytmucTAlung*ATATissue - JmuccytTAlung*ATAasl) + SA_TA*(JoutcytTAlung*
Fu*ATATissueinter - JcytoutTAlung*ATATissue) - SAlysTAtiss * (JTAlunglys * ATATissue -
JlysTAlung * ATATissuelys) - SA_TA*Vmaxpgp*Fu*ATATissue*fncyt_TAlung/(Kmpgp +
Fu*ATATissue*fncyt_TAlung) - SA_TA*Vmaxpgp1*Fu*ATATissue*fncyt_TAlung/(Kmpgp1 +
Fu*ATATissue*fncyt_TAlung));
dxdt_ATATissueinter = 1/VTAtiss_EW * (Qta *(ATATissuevascular/BP - ATATissueinter) -
SA_TA*(JoutcytTAlung* Fu*ATATissueinter - JcytoutTAlung*ATATissue));
dxdt_ATATissuelys = 1/(VTAtisslys) * SAlysTAtiss*(JTAlunglys*ATATissue -
JlysTAlung*ATATissuelys);
dxdt_ATATissuevascular = 1/VTAtiss_Vas *(Qta*(Aarterial - ATATissuevascular) -
Qta*(ATATissuevascular/BP - ATATissueinter));

// PA
dxdt_APAmucus = 1/VPA_mucus * (SA_PA*(JcytmucPAlung*APATissue-JmuccytPAlung*APAmucus)
+  SA_PA*Vmaxpgp*Fu*APATissue*fncyt_PAlung/(Kmpgp + Fu*APATissue*fncyt_PAlung) +
SA_PA*Vmaxpgp1*Fu*APATissue*fncyt_PAlung/(Kmpgp1 + Fu*APATissue*fncyt_PAlung));
```

```
dxdt_APATissue = 1/(VPAtiss-VPAtiss_EW-VPAtisslys-VPAtiss_Vas) * (-
SA_PA*(JcytmucPAlung*APATissue - JmuccytPAlung*APAmucus)  +  SA_PA*(JoutcytPAlung*
Fu*APATissueinter - JcytoutPAlung*APATissue) - SAlysPAtiss * (JPAlunglys * APATissue -
JlysPAlung * APATissuelys) -  SA_PA*Vmaxpgp*Fu*APATissue*fncyt_PAlung/(Kmpgp +
Fu*APATissue*fncyt_PAlung) - SA_PA*Vmaxpgp1*Fu*APATissue*fncyt_PAlung/(Kmpgp1 +
Fu*APATissue*fncyt_PAlung) );
dxdt_APATissueinter = 1/VPAtiss_EW * (Qc *(APATissuevascular/BP - APATissueinter) -
SA_PA*(JoutcytPAlung* Fu*APATissueinter - JcytoutPAlung*APATissue));
dxdt_APATissuelys = 1/(VPAtisslys) * SAlysPAtiss * (JPAlunglys * APATissue - JlysPAlung *
APATissuelys);
dxdt_APATissuevascular = 1/VPAtiss_Vas *(Qc*(Avenous - APATissuevascular) - Qc*
(APATissuevascular/BP - APATissueinter));

// Arterial
dxdt_Aarterial = 1/Varterial * (Qc *(APATissuevascular - Aarterial) - GFR*Fu/BP*Aarterial);

// Venous
dxdt_Avenous = 1/Vvenous * (Qbrain*BP_Fu*Abrain/PNbrain + Qheart*BP_Fu*Aheart/PNheart  +
Qkid*BP_Fu*Akid/PNkid + Qua*AUATissuevascular + Qca*ACATissuevascular +
Qta*ATATissuevascular + Qmuscle*BP_Fu*Amuscle/PNmuscle  + Qskin*BP_Fu*Askin/PNskin  +
Qslow*BP_Fu*Aslow/PNslow  + Qother*BP_Fu*Aother/PNother +
(Qliver+Qgi+Qspleen)*BP_Fu*Aliver/PNliv - Qc*Avenous);

// Brain
dxdt_Abrain = 1/(Vbrain-Vbrainlys) * (Qbrain*(Aarterial - BP_Fu*Abrain/PNbrain) - SAlysbrain *
(Jbrainlys * Abrain - Jlysbrain * Abrainlys));
dxdt_Abrainlys = 1/Vbrainlys * SAlysbrain * (Jbrainlys * Abrain - Jlysbrain * Abrainlys);

// Heart
dxdt_Aheart = 1/(Vheart-Vheartlys) * (Qheart*(Aarterial - BP_Fu*Aheart/PNheart) - SAlysheart *
(Jheartlys * Aheart - Jlysheart * Aheartlys));
dxdt_Aheartlys  = 1/Vheartlys * SAlysheart * (Jheartlys * Aheart - Jlysheart * Aheartlys);

// Kidney
dxdt_Akid = 1/(Vkid-Vkidlys) * (Qkid*Aarterial - Qkid*BP_Fu*Akid/PNkid - SAlyskid * (Jkidlys*Akid
- Jlyskid * Akidlys) - ((Vkid-Vkidlys)*Vmaxrenal*Fu*Akid)/(Kmrenal+Fu*Akid));
dxdt_Akidlys = 1/Vkidlys * SAlyskid * (Jkidlys * Akid - Jlyskid * Akidlys);

// Liver
dxdt_Aliver = 1/(Vliver-Vliverlys) * (Qliver*Aarterial + Qgi*BP_Fu*Agi/PNgut +
Qspleen*BP_Fu*Aspleen/PNspleen - (Qliver+Qgi+Qspleen)*BP_Fu*Aliver/PNliv - SAlysliver *
(Jliverlys * Aliver - Jlysliver * Aliverlys) - CL_Liv*Fu*Aliver);
dxdt_Aliverlys  = 1/Vliverlys * (SAlysliver*(Jliverlys*Aliver - Jlysliver*Aliverlys));

// GI
dxdt_Agi = 1/(Vgi-Vgilys) * (Qgi*(Aarterial - BP_Fu*Agi/PNgut) - SAlysgi * (Jgilys * Agi - Jlysgi *
Agilys)  + KA*GUT);
dxdt_Agilys = 1/Vgilys * SAlysgi * (Jgilys * Agi - Jlysgi * Agilys);
dxdt_GUT = - KA*GUT + KmucUA * AUAmucus*VUA_mucus + KmucUA * AUAasl *VUA_asl;

// Spleen
```

```
dxdt_Aspleen    = 1/(Vspleen-Vspleenlys) * (Qspleen*(Aarterial - BP_Fu*Aspleen/PNspleen) -
SAlysspleen * (Jspleenlys * Aspleen - Jlysspleen * Aspleenlys));
dxdt_Aspleenlys = 1/Vspleenlys * SAlysspleen * (Jspleenlys * Aspleen - Jlysspleen * Aspleenlys);

// Muscle
dxdt_Amuscle   = 1/(Vmuscle-Vmusclelys) * (Qmuscle*(Aarterial - BP_Fu*Amuscle/PNmuscle) -
SAlysmuscle * (Jmusclelys * Amuscle - Jlysmuscle * Amusclelys));
dxdt_Amusclelys = 1/Vmusclelys * SAlysmuscle * (Jmusclelys * Amuscle - Jlysmuscle * Amusclelys);

// Skin
dxdt_Askin = 1/(Vskin-Vskinlys) * (Qskin*(Aarterial - BP_Fu*Askin/PNskin) - SAlysskin * (Jskinlys *
Askin - Jlysskin * Askinlys));
dxdt_Askinlys = 1/Vskinlys * SAlysskin * (Jskinlys * Askin - Jlysskin * Askinlys);

// Slow
dxdt_Aslow = 1/(Vslow-Vslowlys) * (Qslow*(Aarterial - BP_Fu*Aslow/PNslow) - SAlysslow *
(Jslowlys * Aslow - Jlysslow * Aslowlys));
dxdt_Aslowlys  = 1/Vslowlys * SAlysslow * (Jslowlys * Aslow - Jlysslow * Aslowlys);

// Remaining
dxdt_Aother = 1/(Vother-Votherlys) * (Qother*(Aarterial - BP_Fu*Aother/PNother) - SAlysother *
(Jotherlys * Aother - Jlysother * Aotherlys));
dxdt_Aotherlys = 1/Votherlys * SAlysother * (Jotherlys * Aother - Jlysother * Aotherlys);
'
#=============================================================================#
                                          # END #
#=============================================================================#
```
